# ALF: a strategy for identification of unauthorized GMOs in complex mixtures by a GW-NGS method and dedicated bioinformatics analysis

**DOI:** 10.1038/s41598-017-14469-8

**Published:** 2017-10-26

**Authors:** Alexandra Bogožalec Košir, Alfred J. Arulandhu, Marleen M. Voorhuijzen, Hongmei Xiao, Rico Hagelaar, Martijn Staats, Adalberto Costessi, Jana Žel, Esther J. Kok, Jeroen P. van Dijk

**Affiliations:** 1 Department of Biotechnology and Systems Biology, National Institute of Biology, Večna pot 111, SI-1000 Ljubljana, Slovenia; 2 Jožef Stefan International Postgraduate School, Jamova 39, SI-1000 Ljubljana, Slovenia; 3 RIKILT Wageningen UR, P.O. Box 230, 6700 AE Wageningen, The Netherlands; 4 Food Quality and Design Group, Wageningen University and Research, P.O. Box 8129, 6700 EV Wageningen, The Netherlands; 5 College of Food Science and Technology, Nanjing Agricultural University, Jiangsu, 210095 P. R. China; 6 BaseClear, Einsteinweg 5, 2333 CC Leiden, The Netherlands

## Abstract

The majority of feed products in industrialised countries contains materials derived from genetically modified organisms (GMOs). In parallel, the number of reports of unauthorised GMOs (UGMOs) is gradually increasing. There is a lack of specific detection methods for UGMOs, due to the absence of detailed sequence information and reference materials. In this research, an adapted genome walking approach was developed, called ALF: Amplification of Linearly-enriched Fragments. Coupling of ALF to NGS aims for simultaneous detection and identification of all GMOs, including UGMOs, in one sample, in a single analysis. The ALF approach was assessed on a mixture made of DNA extracts from four reference materials, in an uneven distribution, mimicking a real life situation. The complete insert and genomic flanking regions were known for three of the included GMO events, while for MON15985 only partial sequence information was available. Combined with a known organisation of elements, this GMO served as a model for a UGMO. We successfully identified sequences matching with this organisation of elements serving as proof of principle for ALF as new UGMO detection strategy. Additionally, this study provides a first outline of an automated, web-based analysis pipeline for identification of UGMOs containing known GM elements.

## Introduction

Nowadays, the vast majority of feed products in industrialised countries contain materials that are derived from genetically modified organisms (GMOs). For food products the situation is still very different, primarily due to the lack of public acceptance in some countries and amongst specific groups of consumers, but here also a slow trend can be observed of increased use of products derived from GMOs. From the Food and Agriculture Organization of the United Nations (FAO) report of 2014^1^, it can furthermore be seen that the number of incidents with unauthorised GMOs (UGMOs) is gradually increasing. The reported incidents related primarily to identified UGMOs, that had received market approval in other countries. However, in a growing number of cases the incidents related to unknown UGMOs where the mere combination of the crop at hand and the detected GMO elements were deemed sufficient to take action. It should be stressed that so far no genetically modified (GM) crops have been identified to have adverse effects on humans, animals or the environment^2,3^. Nevertheless, in the light of the rapidly expanding diversity of GMOs in experimental settings, it seems prudent to have the methodologies in place to detect and identify UGMOs, including the as yet unknown ones. There is a general lack of specific detection methods for UGMOs, usually due to the absence of detailed sequence information and reference materials.

In recent years, a number of strategies have been developed that focus on the detection of UGMOs. The strategy generally used is based on the screening of samples for a range of GMO elements. Subsequently, the presence of the observed elements is compared with the confirmed presence of authorised GMOs or known UGMOs in the same sample. A mismatch between observed elements and observed GMOs indicates the potential presence of a UGMO. Clearly, only GMOs for which adequate methods for identification are available^4–15^ can be taken into account here. Additional analyses are necessary to determine whether the identified GMO element is indeed linked to an unknown, unauthorised GMO, or rather to, for instance, the native organism of the element. Therefore, in these cases, additional experiments will be required, that are currently usually based on variants of gene walking (GW) strategies where the unexplained elements can be used as a starting point for GW to obtain adjacent sequence information. This sequence information could lead to the identification of a specific UGMO, especially when the sequence information stretches into the flanking genomic region of the GMO insert. This information can ultimately, if deemed necessary, be used to develop a specific method for the identified UGMO.

In the last two decades several GW approaches have been developed and modified for application in the GMO field. Examples are Long template-Rapid Amplification of Genomic DNA Ends (LT-RADE)^16,17^, SiteFinding-PCR^18,19^, APAgene GOLD Genome Walking Kit^20–22^, A-T linker adapter PCR^23^, Randomly broken fragment PCR (RBF-PCR)^24^, Locus-finding PCR (LF PCR)^25^ and Loop-linker PCR^26^ recently reviewed in Arulandhu *et al*.^27^. Most of the approaches have been initially evaluated in a pure GMO sample. The APAgene GOLD Genome Walking Kit^20–22^ was also tested in low GM percentages in combination with fragment cloning and Sanger sequencing^20,22^. Only very recently this method was also succesfully applied in combination with NGS and more complex GMO mixtures^21^.

Any enrichment approach applied to a mixed sample may yield a mixture of sequences, because even a single common element may have several adjacent sequences, from different GMOs in the mixture. Next Generation Sequencing (NGS) is ideally suited for sequencing all amplified fragments in a mixture. In the development of a new GW approach we chose the PacBio RSII as NGS platform, as long reads of single molecules minimize the chance of artefacts, frequently referred to as chimeric sequences, due to incorrect assembly of short sequence reads^19^. Target molecules can be sequenced multiple times or ‘passes’, as hairpin adaptors are ligated to both ends, creating a circular DNA molecule, serving as a polymerase template without an end. The initial output is a polymerase read with multiple copies of the target sequence interspaced by adaptor sequences. When such a subread is present at least four times, a consensus sequence is made after removing the adaptor sequences. This is the circular consensus sequence (CCS) output of the PacBio RS II^28^ (Supplementary Fig. S1). With 80–85% accuracy^29^, subreads have a relatively high error rate^29–32^. As the errors are random, with the error pattern of 10% insertions and 5% deletions^31^, the accuracy of the CCS read is higher than that of the subread, and it increases with the number of subreads. SiteFinding-PCR^18,19^, and APAgene GOLD Genome Walking Kit^21^, are the only enrichment approaches that have been published in combination with NGS analysis to detect and identify GMOs/UGMOs. However, given the random start and two nested PCRs of SiteFinding-PCR, and two semi nested PCRs and the use of DRT primers in APAgene GOLD Genome Walking Kit, these approaches might be sensitive to contamination. Therefore, we adapted the LT-RADE method with a biotinylation primer mediated clean-up prior to PCR and only one round of PCR. Coupling this approach to NGS should allow for simultaneous detection and identification of all GMOs in a sample, including UGMOs, in a single analysis.

In this research, an adapted GW approach was developed, hence referred to as Amplification of Linearly-enriched Fragments (ALF), encompassing the advantages of the available methods, particularly the rapid amplification of cDNA ends (RACE)^33^ approach and the use of biotinylated primers. The efficacy of the ALF approach was assessed on a complex mixture consisting of four GMOs: maize MON810, MON89034, MON88017 and cotton MON15985, using the GMO elements p35S promoter and tNOS terminator as starting-points for the elongation. After amplification of the elongated fragments, all obtained fragments were sequenced using the PacBio RSII platform. The complete insert and genomic flanking regions were known for the first three GM maize events, while for the MON15985 only partial sequence information was available; combined with a known organisation of elements, this GMO served as a model for a UGMO in this set-up. In the present study, the ALF approach is presented and applied on the mixture of all four GMOs. The first outline of an automated, web-based analysis pipeline for identification of UGMOs containing known GM elements was set, and the results of the new strategy are evaluated in the light of the necessity to have adequate methodology in place to identify GMOs for which limited information is available.

## Results

### Amplification of Linearly-enriched Fragments (ALF)

**Figure 1:**
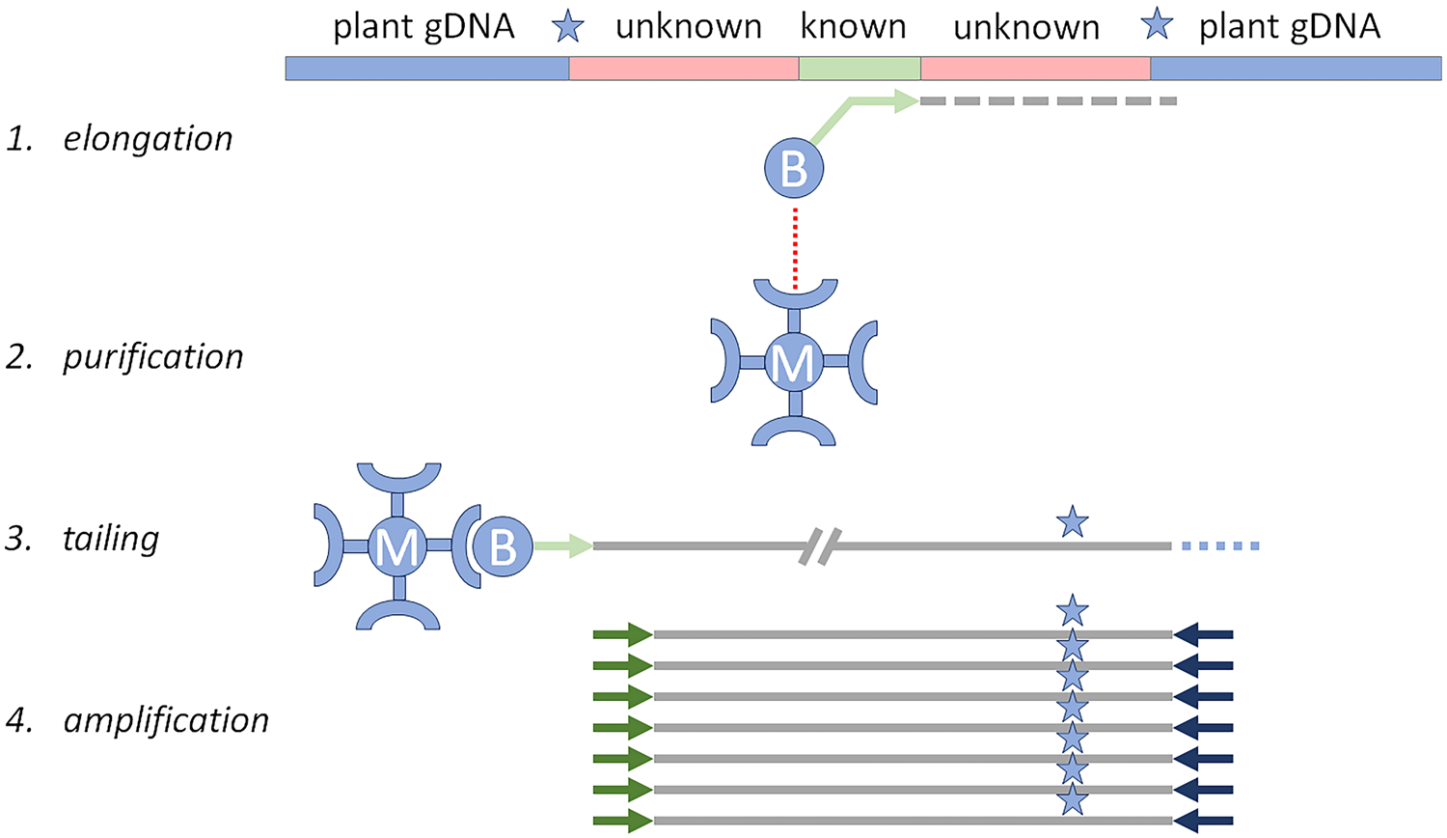
Schematic overview of the ALF procedure. The procedure yields dsDNA molecules after initial primer elongation, of various length, the longest of which will pass a construct-genome boundary (star), leading to GMO identification.

**Figure 2:**
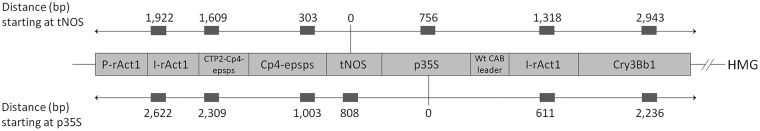
Distances of several qPCR targets from the enrichment starting points in the MON88107 GMO, used for evaluation of the ALF protocol. The light grey boxes indicate the different elements in the GMO. The upper and lower line indicate the enrichment for tNOS (upper) and p35S (lower), 0 indicates the starting points of enrichment and arrows indicate the direction of enrichment (upstream or downstream). The dark grey boxes indicate qPCR targets used for evaluation of the ALF protocol and distance of the qPCR targets from the starting points.

**Figure 3:**
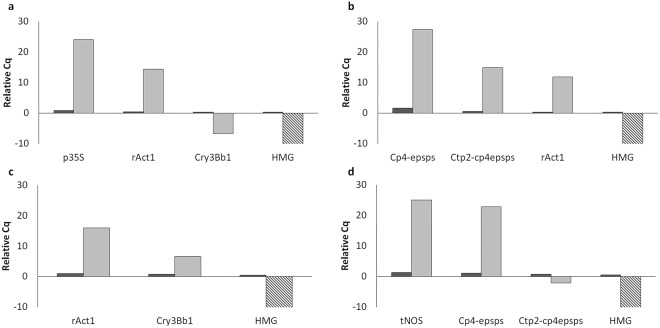
Length-dependent increase of specific targets and loss of genomic background shown with qPCR. In all cases the amplicon closest to the element targeted for linear enrichment showed the highest increase in signal, expressed as the relative Cq value and calculated by subtracting the Cq after LE (dark bars) or snPCR (light bars) from the Cq of the starting material. This means the relative Cq value for each starting point is zero. Panel a: Enrichment for tNOS downstream. (**b**) Enrichment for tNOS upstream. (**c**) Enrichment for p35S downstream. (**d**) Enrichment for p35S upstream. In all panels, the dashed bars for HMG indicate a reduction beyond detection.

A protocol was developed for the identification of unknown GMO-related sequences starting from known GMO elements, called ALF: Amplification of Linearly-enriched Fragments (Fig. 1). The success of the ALF protocol was determined both in terms of quantity and length of the molecules of interest. In the reference materials used, upstream and downstream GMO elements were known. By performing qPCR analysis prior to, and at different steps of the procedure, relative increase in target fragments was estimated. Targets for the qPCR analysis were chosen at various distances from the start of the LE (Fig. 2). To increase the reliability of the quantification outcomes, Joint Research Centre (JRC)-validated qPCR methods were used in combination with certified reference material for >99.05% MON88107. qPCRs were performed before the procedure in the starting material (SM), after LE, and after snPCR. Apart from the qPCRs targeting the GM elements of interest, also the abundance of the maize endogenous high mobility group (*hmg*) gene was evaluated with qPCR, to monitor the removal of genomic DNA (Fig. 3). After the LE step, a slight increase was observed for targets of interest, more prominently for targets closer to the starting point of LE. As expected, the genomic DNA amount was very similar before and after LE. After snPCR, a large increase in targets of interest was observed, again more prominent for target sequences close to the LE start, indicating a size-dependent enrichment. Loss of gDNA beyond detection was observed for all targets after the snPCR step (Fig. 3).

The relative amount of the different targets was expressed as the relative Cq value and calculated by subtracting the Cq after LE or snPCR from the Cq of the starting material. The largest increase in relative Cq was observed for GW starting from tNOS in the upstream direction, for the element closest to the LE start, Cp4-epsps (Fig. 3, panel B). The fold change for the different targets upstream of tNOS was estimated using a ΔCq approach, assuming a qPCR efficiency of 2. The Cq for the Cp4-epsps element, 303 nt upstream of the tNOS LE primer, was 30.3 in the starting material. During development of the method, we found it to be necessary to dilute the snPCR material 100-fold prior to qPCR evaluation, in order to obtain Cq values higher than five. Even in the diluted snPCR material the Cq value for the Cp4-epsps element was as low as 9.6. This was converted to the theoretical value of 2.96, by subtracting the 6.64 cycles from this Cq value, corresponding to a 100-fold dilution. The ALF related ΔCq for the Cp4-epsps element was therefore calculated to be 27.3 cycles, indicating an estimated increase of 165 million-fold of fragments at least 303 nt long. A fraction of these molecules was at least 1609 nt long, evidenced by the increase in relative Cq value for the Ctp2-cp4epsps element, located 1609 nt upstream of the tNOS LE primer. This fraction was estimated to be enriched 30 thousand fold. Likewise, a subfraction of these fragments was at least 1922 nt long, based on relative rAct1 Cq increase. This fraction was estimated to be enriched 3.5 thousand fold.

### PacBio sequencing of a GMO mixture after ALF

A DNA-based mixture was made using DNA extracts from four reference materials, in an uneven distribution, mimicking a real life situation, where one or several GMOs may be present in a much lower concentration than others in the same mixed sample. DNA of MON810 from a co-existence field trial^34^ was used as the most abundant GMO and was present in 97%. Of the other three GMO DNA isolations, 1% relative weight was added for each (Table 1). Two maize lines were used, MON89034 and MON88017, and one cotton line, MON15985. This mixture was subjected to the ALF protocol in four separate reactions for the different elements and directions: the p35S promoter and the tNOS terminator, both in upstream and downstream direction. The reactions were pooled and sequenced on the PacBio RSII platform. The results of sequencing were 411 CCS reads (length distribution is shown in Supplementary Fig. S2).

**Table 1:** Details of reference materials^49^.

GMO	Content in mixture (%)	Estimated copy number	Supplier	Code	Description	Element order*	Donor organism
MON810	97	~40.000	Field trial^34^	In-house	50% MON810, ground corn	P-e35S	Cauliflower mosaic virus
						I-hsp70	*Zea mays*
						CS-cry1Ab	*Bacillus thuringiensis*
MON89034	1	~400	AOCS	0906-E	>99.42% MON89034, ground corn	V-LB	*Agrobacterium tumefaciens*
						P-e35S	Cauliflower mosaic virus
						L-cab	*Triticum aestivum*
						I-1_act1	*Oryza sativa*
						CS-cry1A_105	Synthetic
						T-hsp17_3	*Triticum aestivum*
						P-FMV	Figwort mosaic virus
						I-hsp70	*Zea mays*
						I-1_rbcS	*Zea mays*
						CS-cry2Ab2	*Bacillus thuringiensis ssp*. *Kurstaki*
						T-nos	*Agrobacterium tumefaciens*
						V-RB	*Agrobacterium tumefaciens*
MON88017	1	~400	AOCS	0406-D	>99.05% MON88017, ground corn	P-act1	*Oryza sativa*
						I-1_act1	*Oryza sativa*
						TP-ctp	*Arabidopsis thaliana*
						CS-CP4epsps	*Agrobacterium tumefaciens ssp*. *CP4*
						T-nos	*Agrobacterium tumefaciens*
						P-e35S	Cauliflower mosaic virus
						L-cab	*Triticum aestivum*
						I-1_act1	*Oryza sativa*
						CS-cry3Bb1	*Bacillus thuringiensis ssp*. *Kumamotoensis*
						T-hsp17_3	*Triticum aestivum*
MON15985	1	~400	AOCS	0804-D	>98.45% Bollgard II cotton, ground cotton seed	P-e35S	Cauliflower mosaic virus
						L-hsp70	Petunia hybrida
						TP-ctp	*Arabidopsis thaliana*
						CS-cry2Ab2	*Bacillus thuringiensis ssp*. *Kurstaki*
						T-nos	*Agrobacterium tumefaciens*
						P-e35S	Cauliflower mosaic virus
						CS-cry1Ac	*Bacillus thuringiensis ssp*. *Kurstaki*
						T-7Salpha	*Glycine max*
						P-35S	*Cauliflower mosaic virus*
						CS-nptII	*Escherichia coli*
						T-nos	*Agrobacterium tumefaciens*
						P-e35S	Cauliflower mosaic virus
						CS-uidA	*Escherichia coli*
						T-nos	*Agrobacterium tumefaciens*
						CS-aadA	*Escherichia coli*

*Initial capital characters: P = promoter, I = intron, CS = coding sequence, V = vector, LB = left border, L = leader, T = terminator, RB = right border, TP = transit peptide.

### Sequence analysis: database construction

To analyse the CCS reads gained after PacBio RSII sequencing three databases were constructed: an event, a Constructs-And-Flanks (CAF) and an element database. The event database (Supplementary Data S1) consisted of the most likely amplicon sequences of the event specific qPCR methods used for quantification of events (MON810, MON88017, MON89034 and MON15985) in the sample. Sequences of primers and probes, together with the number of unknown nucleotides between them, were taken from the EU Database of Reference Methods for GMO Analysis (GMOMETHOD) (http://gmo-crl.jrc.ec.europa.eu/gmomethods/) and from the method validation reports^35–38^. These sequences were queried against the NCBI patent database (Table 2). After inspection, the top hit for each of them was added to the event database.

**Table 2:** Event database sequences and corresponding NCBI accession numbers.

GM event	Event sequences	Best hit accession number
MON810	TCGAAGGACGAAGGACTCTAACGT***TT***AACATCCTTTGCCATTGCCCAGC***TATCTGTCACTTTATTGTG***AAGATAGTGGAAAAGGAAGGTGGC*	AR490568
MON89034	TTCTCCATATTGACCATCATACTCATT***GC***ATCCCCGGAAATTATGTT***TT***TTTAAAAACCACGGTATTATAGATACCG	FV532179
MON88017	GAGCAGGACCTGCAGAAGCT***AGCTTGATGGGGATCAGATTGTCGTT***TCCCGCCTTCAGTTTAAACAGAGTCGGGT***T***TGGATGGTCAACTCCGGCA	DJ058152/DJ058151
MON15985	GTTACTAGATCGGGGATATCC***CCGGGGCGG***CCGCTCTAGAACTAGTGGATCTGCACTGAA***A***TCCCATCCATTTAGCAACCTT	EA135634

*Nucleotides in italics denote the string of unknown nucleotides between primer and probe sequences in the search template.

The CAF database consisted of the available construct sequences and flanking plant genomic regions of individual events in the sample (Supplementary Data S2). In case of MON15985 only partial 3′ and 5′ insert sequences with corresponding flanking genomic sequences were available. For MON810 two reference sequences were available: sequence JQ406879, covering the 5′ flank and the p35S promoter, and AY326434, covering the insert from the p35S promoter onwards to the 3′ flank. These sequences showed a 75-nucleotide overlap. In the initial workflow both sequences were in the CAF database, but the merged MON810 sequence of JQ406879 and AY326434, named RIKILT20151130, was used in the final workflow (Supplementary Data S2).

The third, element database, consisted of element sequences present in the experimental mixture. All element sequences from individual lines were gathered. For MON810, MON89034 and MON88017 complete reference sequences were already known and annotated. These reference sequences were divided in elements based on their annotation, for which a General Feature Format (GFF) file was constructed (Supplementary Data S3). For MON15985, for which only the order of elements was known, generic sequences for these elements were taken from the NCBI patent database. Initially, this database contained all element sequences (Supplementary Data S4). To reduce redundancy, the final version contained only the longest sequence of elements with several entries in the first version (Supplementary Data S5). All designed databases were transformed into BLAST+ databases^39^ and imported into the open, web-based analysis platform Galaxy^40^.

### Building the workflow for GMO sequence identification

**Figure 4:**
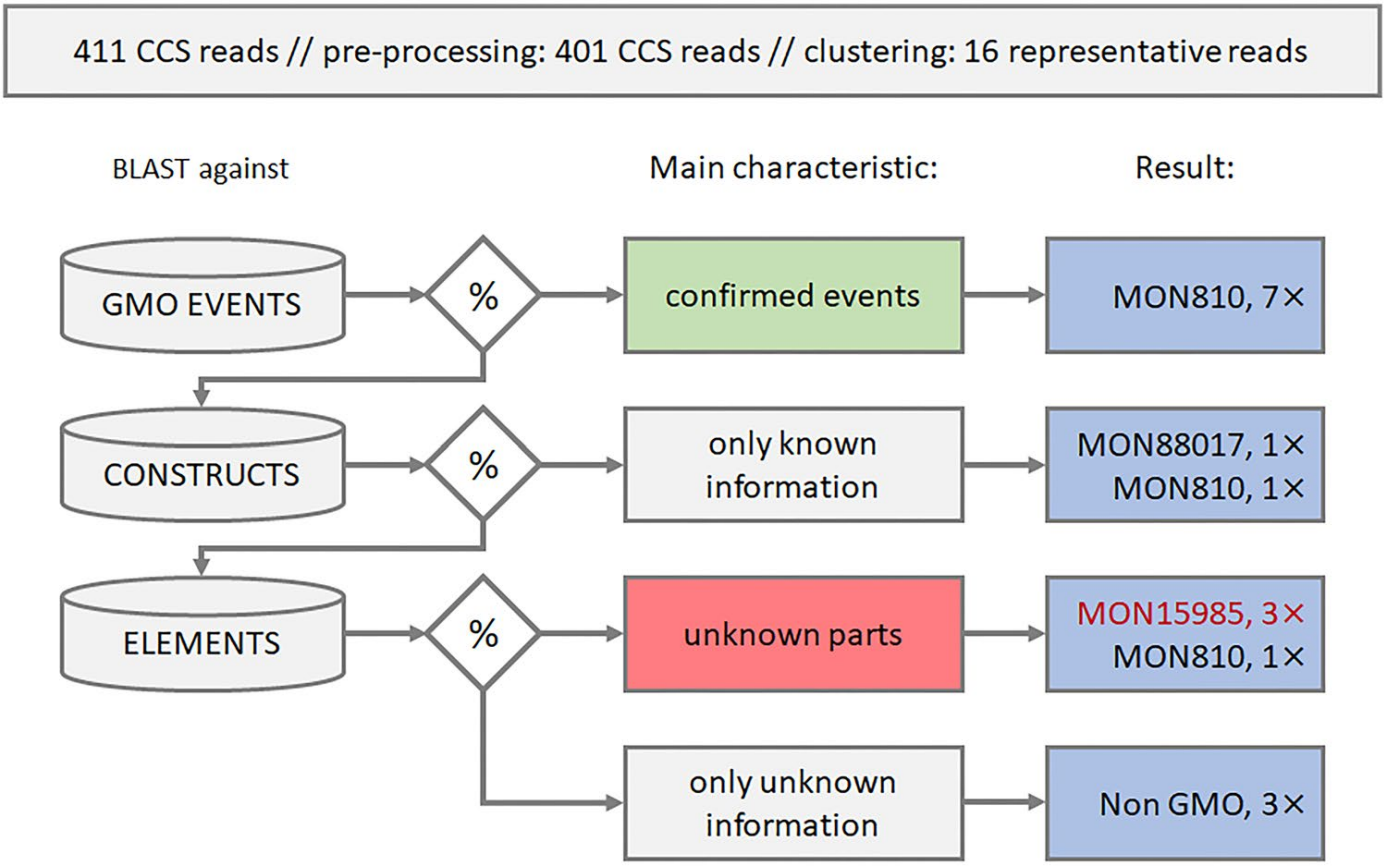
Schematic overview of the data analysis workflow. CCS reads are first processed to reduce noise and redundancy. Representative reads are then grouped in relevant bins based on homology with sequences in different databases, using blast. In the results, reads likely to be related to MON15985 are marked in red as this authorised GMO with incomplete sequence information served as a model UGMO in this study.

The workflow for GMO sequence identification in the samples was constructed in Galaxy to answer the following questions: (1) is there any potential evidence for UGMOs, (2) can known GMOs be identified and (3) can a list of potential GMOs be prepared. Considering these questions, a workflow with six main steps was constructed (Fig. 4 and Supplementary Fig. S3).

Step 1 consisted of a trimming and length selection; removing artificial sequences from the CCS reads and subsequently discarding short reads. The poly-dC tail and AAP adaptor sequences were removed and a length filtering was applied at >50 nt. All 411 CCS reads were used as the workflow input, a total of 401 reads proceeded to step 2.

Step 2 was selection of reads containing an enrichment primer, i.e. the nested tNOS and p35S LE primers, to make sure only specific sequences were kept. A total of 383 sequences passed the LE primer filtering; 368 were enriched for the p35S and 15 for the tNOS.

Step 3 was a clustering, using UPARSE^41^, of CCS reads to further reduce the number of sequences. A three-step clustering approach was used. In this approach, sequences were dereplicated, sorted by abundance and clustered. Due to the nature of PacBio sequencing a CCS read either starts or ends with the enrichment target element, in this case the tNOS or p35S. An additional step of reverse complementing was applied to all the reads containing the enrichment target element on the 3′ end before dereplication, since the clustering algorithm aligned sequences only in plus/plus manner. With a minimum cluster identity, also termed cluster radius, of 0.97, 383 CCS reads were clustered in 16 clusters. For each cluster, the longest CCS read was used as the representative sequence, and termed cluster representative CCS read (crCCS read).

Step 4 was a Megablast of the crCCS read against the event database. Seven of the 16 crCCS reads showed a match to a sequence in the database with an identity of 95% or higher and a minimum coverage of 97%. The main characteristic of these reads was that they included a *confirmed event*, and were not analysed further. The nine remaining crCCS reads were further processed in step 5.

Step 5 was a Megablast of the remaining crCCS reads against the CAF database. Two out of nine crCCS reads aligned completely to a CAF database sequence, i.e. full query coverage, and were annotated using the intersect interval tool (part of BEDtools^42^). The main characteristic of these annotated crCCS reads was the establishment that they contained *only known information*.

Step 6 was a Megablast of the remaining seven crCCS reads against the element database, aiming at identification of reads that contain both known and unknown sequence, as is expected for UGMOs. The crCCS reads were divided into two bins, also based on their main characteristic, either containing *unknown parts*, or containing *only unknown information*. The reads containing unknown parts also contained homologies to known elements, by the definition of this workflow. The Blast output of these reads against the element database was sorted in a way that the top hit was the one with the longest sequence alignment. The alignments with different elements were ordered according to their position in the CCS read, from 5′ to 3′. All four output bins, *confirmed events*, *only known information*, *unknown parts*, and *only unknown information* were imported in an excel template, constructed to give a user-friendly output (Supplementary spreadsheet 1).

### Connecting sequences with the experimental set-up

**Figure 5:**
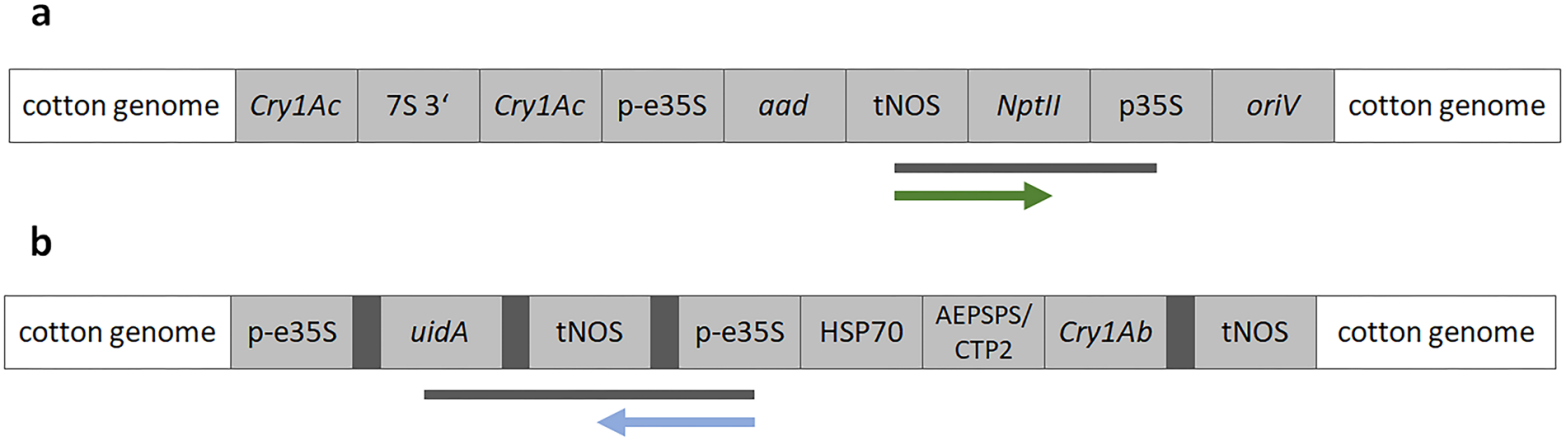
Two partial sequences of the MON531 and MON15985 inserts. Panel a shows the alignment of crCCS reads 133951 and 45207 to the insert and flanking region of MON531, with the position of enrichment primer (downstream NOS terminator). Panel b shows the alignment of and crCCS read 156962 to the insert and flanking region of MON15985, and the enrichment primer (upstream 35S promotor). Both sequences cover a previously unknown insert sequence.

Relating to the three main question (1) is there any potential evidence for UGMOs, (2) can known GMOs be identified and (3) can a list of potential GMOs be prepared, the crCCS reads in the *unknown parts* bin were the most informative for the first question (Fig. 4 and Supplementary Fig. S3). The four crCCS reads in that bin showed three different element orders. One was *tNOS* – *nnn* - *NPT II* – *nnn* - *p35S*, present in two crCCS reads in both orientations, where *nnn* denotes an unknown sequence. This element order was consistent with that of the MON15985 parental line MON531. The second order was *p35S* – *nnn* – *tNOS* – *nnn* – *UidA*, consistent with the order in the retransformation construct of MON15985 (Fig. 5 and Supplementary Data S6). Sequences with these two element orders were previously not linked to any MON15985 designated sequence in a public database. The third element order was *p35S* – *nnn - hsp70 - cry1ab*, corresponding to MON810. The gap between the p35S and HSP70 was 16 nucleotides, and was the result of an incomplete alignment with the p35S element reference sequence.

In addition to the four crCCS reads in the *unknown parts* bin, three crCCS reads (*120091*, *43434*, and 106520) ended up in the *only unknown information* bin (Fig. 4). To find the origin of these reads an NCBI Blastn, against nucleotide database, and a Megablast and a Blastn against NCBI patent database were run. For read 120091 a 99% identity and a 98% CCS read coverage against *maize genotype CMS-S mitochondrion* (DQ490951) was observed. For read 106520 top hits were *nucleotide sequences and polypeptides encoded thereby useful for modifying plant characteristics* (GP689587) and *long intergenic noncoding RNAs in maize* (JC761506). A total of 76% of the read was covered. For read 43434 the top hits were described as a *composition and method for therapy and diagnosis of ovarian cancer* (DL059073), with 90% identity and query coverage of 60% (Supplementary Table S4).

The *confirmed events* bin, as the result of step 4, provided the answer to question (2) on identification of known GMOs. In order for a crCCS read to be acknowledged as containing a confirmed event, a 97% minimal coverage of the event sequence in the database was required, with at least 95% identity (Table 3). All seven reads that fulfilled the criteria of step 4 originated from MON810 (Fig. 4). In one of these, the read aligned to the same database event sequence twice, both with a 100% identity match. After closer investigation, the CCS read turned out to contain a complete repetition of one sequence; an AAP primer with a poly G tail prior to a 5′ MON810 flanking region followed by a p35S promoter sequence. The sequence was kept in the *identified GMOs*.

**Table 3:** Results of NCBI BLAST+ against the event database.

crCCS read	Identified GMO	% of identical match	Alignment length	Length of db sequence	Alignment length in %
33879	MON810	100.00	92	92	100
40876	MON810	95.65	92	92	100
78816	MON810	98.92	93	92	101
119658	MON810	96.81	94	92	102
128024	MON810	100.00	92	92	100
132414	MON810	96.74	92	92	100
158734*	MON810	100.00	92	92	100
158734*	MON810	100.00	92	92	100

^*^CCS read 158734 showed two perfect alignments with the database sequence.

In step 5, a bin was made for the fully annotated CCS reads, containing *only known information*. In this step the answers to the question (3) on the list of potential GMOs were found. An indication of MON88017 and MON810 was observed (Fig. 4). Two crCCS reads completely aligned with a database sequence, the first CCS read had the element order of *p35S - hsp70 - cry1ab*, present only in MON88017 and the second *cp4epsps* – *tNOS* – *35S* present only in MON810, considering the GMOs used in this experiment.

### The merged MON810 reference sequence

The merged MON810 read RIKILT20151130 (deposited at The European GMO reference database - Euginious http://www.euginius.eu/euginius/pages/sequence_string.jsf?sequence = 4165286415439512999) that comprised of sequences JQ406879 and AY326434 was compared to the CCS reads. Bowtie2^43^ mapping with default settings of the CCS reads was performed using very sensitive local alignment (Supplementary Fig. S4). The CCS reads were first filtered by quality, only those that had a quality score of 33 or more over at least 90% of the sequence were accepted. Out of the initial 411 CCS reads, 165 filtered sequences aligned to this reference. A total of 133 CCS reads covered the overlap between sequences JQ406879 and AY326434, confirming the trueness of the new, merged MON810 reference sequence.

## Discussion

The main goal of this study was to develop a protocol for identification of UGMOs in a complex mixture. The new approach was successful in its aim, and a proof of principle was found by identifying previously unknown sequences corresponding to the element order in a GMO.

The adapted GW approach, ALF, aimed to enrich the adjacent regions of the target prior to sequencing. Enrichment decreased the required sequencing depth and cost, as well as the data analysis time and cost. A new combination of GW steps was conceived and tested. Two common approaches were combined: gDNA reduction and the RACE/LT-RADE principle of tailing and semi-universal PCR. Biotinylation of the enrichment primer was used for background reduction. Since genomic DNA background was already reduced beyond detection, we decided against the use of a nested PCR approach. The semi-nested PCR in this paper is actually a first round of PCR. It is called semi-nested because one of the primers is nested, i.e. slightly downstream of the primer used in the LE step. PCR in general, and nested PCR in particular, is prone to contamination. For most diagnostic laboratories, the use of nested PCR is something to be avoided, especially in case of many repetitive tests. A contamination of a GMO testing laboratory with especially common GM elements would severely compromise an efficient GMO screening using the matrix approach. Target-specific LE primes as well as the nested primers of the snPCR were designed on the basis of the available qPCR primer-probe sequences for the targeted GMO elements, in our case p35S promoter and tNOS terminator. Primer and probe sequences of an unexplained GMO element test might be the only basis for finding related sequences, in case of looking for unknown GMOs as the result of a GMO matrix approach outcome. Therefore, the primer and probe sequences are a logical starting point for enrichment.

The high quality of the CCS reads warranted the omission of an extra quality check at the beginning of the analysis pipeline. This is important, as the library preparation requires a high amount of DNA, with low CCS read counts resulting from a lower DNA input. In this experiment, 411 CCS reads were generated despite a high amplification of relevant molecules in the ALF protocol. A clustering step was added to the workflow to further reduce the number of redundant CCS reads. To make sure no information is lost during clustering, a workflow omitting this step was tested, and it showed no extra information. The largest decrease in redundancy was observed in the *confirmed event* bin, where the decrease of sequences was more than 97% (295 *vs* 7 sequences). However, the main difference influencing the time of further analysis was observed in the *only known information* and *unknown parts* bins, where the number of sequences was decreased more than 92%; 28 *vs* 2 sequences after clustering in the *only known information* bin and 53 *vs* 3 sequences in the *unknown parts* bin. To reduce the time for clustering, a length filtering set at 50 nucleotides was applied in the very first step of the workflow. Reads of this length are not informative, as the shortest transgenic element in our element database is 60 base pairs.

The experimental sample was composed of known GMOs in different quantities: MON810 maize– 97%, MON89034 maize– 1%, MON88017 maize– 1%, and MON15985 cotton– 1%, or expressed in estimated copy numbers: the MON810: ~40.000 estimated copies, and the other three: ~400 copy numbers. This approach is somewhat different from the approach taken by Fraiture *et al*.^21^. They used lower copy numbers (20 in their lowest mixtures), but in equal amounts, for three GMOs. Both situations may actually occur in real samples that may be contaminated by UGMOs. For three GMOs the complete insertion sequence including the corresponding flanking genomic sequence was known. For one GMO, MON15985 cotton, only the element order and partial 5′ and 3′ insert sequences with corresponding flanking genomic sequence were available. Furthermore, MON15985 is a retransformation of another GMO cotton, MON531. The MON531 sequence was also unknown, with the exception of the element order. Therefore, MON15895 mimicked a UGMO in this experimental set-up.

The results can be looked at from two different perspectives. The first is whether or not the workflow enabled identification of UGMOs. The second is whether or not all the input sequences were detected. The transgenic cotton mimicked a UGMO, as explained above. Element orders corresponding to MON15985, to both constructs, were found in the *unknown parts* bin. By finding these two sequences, it was shown that a UGMO containing a known element can be identified. The sequences as such were not before specifically annotated to be part of the MON19585 and MON531 constructs. Event-specific sequences for either event were not found. The MON15985 event-specific test is at 3′ end of the insert. We therefore expected MON15985 in the *confirmed events* bin, through reads starting from tNOS in downstream direction. However, the tNOS enrichment was less successful than p35S enrichment. Out of 383 sequences, 368 were enriched starting from p35S and only 15 from tNOS, and upstream enrichment was in both cases seven times more successful than downstream. This is indeed a striking difference, and seemingly in conflict with the observation in the qPCR evaluation. Potential explanations would be variability in the procedure and or the samples, as the NGS sample (mixture of GMOs) was different from the qPCR evaluated sample (single GMO), or, some kind of bias in the library preparation/sequencing run. Noteworthy, Fraiture *et al*. also found a large variation in numbers of reads related to the different starting points and directions they used for GW^21^. Of the 16 CCS reads, two were tNOS enriched, one upstream and one downstream. The one downstream was indicative of the MON15985 GMO, via the tNOS-nnn-NTPII-p35S sequence present in the MON531 construct of MON15985 (Fig. 5).

From the other perspective, finding back the input transgenes was partially successful. Out of the 16 crCCS reads, 13 could be unequivocally linked to one of the four input sequences, MON810, through reads starting from p35S in upstream direction (Fig. 4). We could not find back event-specific sequences for MON89034 and MON19585 since the event-specific tests are at 3′ end of insert, and tNOS reads were much less abundant than p35S ones. Still, there was sequence evidence linked to the presence of MON88017 and MON15985, the latter one is discussed above. For MON88017, a partial insert sequence covering cp4epsps, the tNOS and the p35S was found. This clearly indicated the presence of MON88017, as no other transgenic line contained this sequence in this experiment. The MON88017 event-specific sequence was actually not expected to be found in the dataset, as both p35S and tNOS are present in the middle of the inserted sequence and in order to reach the point of insertion the upstream or downstream enrichments should cover ~3.5 kb. The longest CCS read was approximately 2.5 kb. Without detection of the point of insertion, the presence of MON88017 and MON15985 cannot be asserted with the same certainty as the presence of MON810. Some other transgenic lines containing the same element order could have been present in the reference material used for sample preparation. As a matter of fact, the *nptII* cassette with the CDS flanked by a p35S and tNOS is found in several GMOs, e.g. MON87460 and MON863 maize, and MON757 and MON1698 cotton.

Finding back specific sequences depends on the databases used. Elements are the building blocks of a transgenic sequences. Elements with the same function, the p35S for instance, do not always have the same sequence length in all transgenic lines. This makes building of an element database more difficult and the database larger than desired. For this reason, a local database was built containing only the longest sequence for each element. The drawback of such a database is that although element sequences are very similar they are not identical. This needs to be considered when interpreting the results. A 75% coverage of a p35S does not necessarily mean that there is an incompletely explained read with a gap between elements: it could be that this certain promoter sequence is not completely identical to the one in the transgenic line. Such was the case for the crCCS read with the event sequence *p35S* – *nnn - hsp70 - cry1ab*, where a 16 nucleotide gap was found between 35S promoter and HSP70. This sequence was placed in the *unknown parts* bin, where potential UGMOs are expected, although further investigation of this read showed that it belonged to MON810. Such a bias might be resolved by addition of all versions of a particular sequence to the database and considering the bit score of the hits after BLAST analysis. Implementing the ‘filter and sort’ tool in Galaxy would allow this, and this addition is recommended for future use of this pipeline. Another database challenge lies in element sequences not always being annotated in the same manner in all database entries. For instance, there is a possibility that a part of a plasmid used in the process of transformation can be included into the element annotation in some database entries and not in others.

The identification of three crCCS reads without any known GMO homology was not expected. All three reads contained at least partial enrichment primer sequences at 5′ or 3′ end, and therefore passed through the enrichment primer filter, pointing to an unspecific binding of enrichment primers to a sequence other than p35S or tNOS. In case of crCCS reads 120091 and 43434 the sequences can be explained as maize mitochondrion and a non-plant sequence, respectively. For crCCS read 106520, however, NCBI patent database Blast identified this sequence as part of patents US756989 and WO2014036048A1, sequences accession number GP69587 and JC61506, respectively, with the field of invention for both patents described as *related to methods altering gene expression* (Supplementary Table S4). While the crCCS read contained only the p35S enrichment primer and not the p35S promoter sequence, there is still a possibility that this sequences could be left over from the genomic transformation. In the current version of the element database we only included the known elements of the GMOs in the study, and actually left out the elements known to be present in the mimic-UGMO, such as nptII. Obviously, *nptII* should be added to the database in a real life situation. Likewise, any homology leading to identification of previously unknown elements should be added to this database, when verified. Within the current three hits, besides the titles, there were no real solid clues for a GMO related sequence. In Table S4, the query and subject starts and ends are given, plus the description of this region in the subject. None of the three reads showed any known GMO related sequence besides the presence of the primer. Therefore, the identification of a previously unknown GMO element, harbouring the 35S primer sequence, is a possible explanation. Another, perhaps more likely explanation would be off-target hybridisation of the 35S primer. In either case, if proven repeatable, this sequence might perhaps be added to one of the dedicated databases in the pipeline, probably the element database, with an annotation such as: previously found potential GMO-related sequence.

A new MON810 reference sequence was combined from previously known sequences JQ406879 and AY326434. There is overlap between sequences JQ406879 and AY326434 in the p35S promoter region. With 133 CCS reads mapping to this overlap, sufficient evidence was given for merging these two reference sequences in one reference sequence named RIKILT20151130. RIKILT20151130 replaced the JQ406879 and AY326434 sequence in the final workflow (Supplementary Data S2).

The main objective of the analysis pipeline was to guide the end-user as quickly as possible to those sequences that require further investigation. For identification of UGMOs, known authorized GMO events are merely nice to know, and reads completely covered by a known construct sequence are of even lower priority. Artefacts should be recognized and filtered, independent of where in the procedure they might occur. For this reason, the reads were set apart that contained only known primer sequences and not any further GM element homology. The placement of reads in separate bins instead of removing reads from the output, enables the end-user to further detail the analysis at any point in the procedure. The 18 CCS reads without a primer were not investigated further. The reasoning behind this was that all sequences should be related to the experiment that was performed. If none of the primers used in the experiment could be found, the link of such a sequence to the experiment itself is weaker. On top of that, if some of these did actually contain a primer, but with too many errors, the quality of the rest of the sequence would also be less reliable, increasing the risk of having to manually explore false positives. The contents of all other bins were analysed and a likely artefact was found in the *confirmed events* bin. One read showed homology to two event sequences instead of one. The raw CCS read, before trimming and primer filtering, turned out to consist of an AAP primer with a poly G tail, 5′ MON810 flanking region followed by p35S sequence, with this motive being repeated. The repetition in itself is already rather strange, and less likely to be a true molecule because of the simple reason that if so, chances are that it was published already, given the well sequenced nature of the MON810 GMO. The presence of primers used in the experiment at the very borders of the repeated sequence makes it near impossible that this sequence is not an experimental artefact. A very likely explanation is a ligation artefact, i.e. the same molecule was ligated twice in a row between smartbell adapters. In the potential UGMO output, an element order consistent with MON810 was identified, besides the relevant sequences for the partially unknown GMO. This read should have been placed in the *only known information* bin, but was not, due to threshold settings. We did choose to keep the threshold as it was. A lowering might easily solve the problem in this dataset, yet it might cause false negative discoveries in others. In this setting, it serves to illustrate the point that UGMO discovery may never be free of false positives. Therefore, all these potential discoveries should be confirmed independently, preferably by design of a novel PCR, based on the newly found sequence. Within the current dataset 2372 polymerase reads containing 11.538 subreads were present. Of those, 411 polymerase reads contained more than four subreads of high enough quality to be merged into a CCS read. Those 411 could be further clustered, based on sequence homology, into 16 distinct sequences. Only four of those needed hands-on evaluation, of which three proved ‘unknown’. All this was automated, meaning that out of a potential 11.538 reads, only 4 had to be manually checked, and three of those rightly so. In case this is a true new, potential UGMO sequence, this sequence should then be confirmed through Sanger sequencing of the PCR amplicon, from an independent DNA isolation of the suspect sample.

In summary, we conceived and tested an integral approach for a lab-based target enrichment based on a suspect GM element, followed by NGS and data analysis by design. We successfully showed the identification of partial sequences of a model UGMO. Future experiments will be aimed at further testing the approach in more complex mixtures, and the current settings of the pipeline design. This study provides a first outline of an automated, web-based analysis pipeline for identification of UGMOs containing known GM elements.

## Methods

### Description of certified reference materials

Certified reference materials (CRMs) of MON89034, MON88017, and MON15985 and the well-characterized reference material MON810 from a field trial^34^ were used for the preparation of complex mixtures. For detailed information on the reference materials see Table 1.

### DNA isolation and preparation of the mixture

Per CRM DNA was isolated using a CTAB extraction followed by the Qiagen DNeasy plant mini kit (Qiagen) according to Scholtens *et al*.^5^. 100 ± 10 mg of dry material was weighed and extraction was performed by adding 700 µl of CTAB buffer (20 g/L CTAB, 1.4 M NaCl, 0.1 M Tris, 20 mM Na2EDTA), 200 µl of nuclease-free water (Life Technologies) and 5 µl of Rnase A (Qiagen,100 mg/µl) and incubated for 15 min at 65 °C in a thermo shaker at 250 rpm. Subsequently, 20 µl of proteinase K solution (Fermentas; 20 ng/µl) was added and the mixture was incubated in the thermo shaker for another 30 min at 65 °C, 250 rpm. To precipitate detergent, proteins, and polysaccharides 200 µl of Buffer P3 (Qiagen, DNeasy plant mini kit) was added to the lysate, the mixture was mixed and cooled on ice for 5 min. After cooling on ice, the manufacturer’s protocol (Qiagen, DNeasy Plant Handbook 10/2012) was followed starting from step 10. The quantity and purity of the isolated DNA was assessed from Nanodrop absorbance measurements (Nanodrop 1000 instrument, Thermo Fisher Scientific). The mixture was prepared by combining 2.68 µl of MON15985 (18.68 ng/µl), 0.91 µl of MON88017 (54.92 ng/µl), 1.09 µl of MON89034 (45.79 ng/µl), 38.4 µl of MON810 (126.4 ng/µl) and 57 µl of nuclease-free water (Life Technologies).

### Linear enrichNment

Linear enrichment (LE) was performed in four separate reactions: p35S up, p35S down, tNOS up and tNOS down. Each 20 µl reaction contained: 1× Buffer 1 (17.5 mM MgCl2, Expand Long Template PCR System, Roche), 200 µM dNTPs (10 mM dNTP mix each, Invitrogen), 3.75 U polymerase blend Taq + Tgo (Expand Long Template PCR System, Roche), 125 nM biotinylated enrichment primer and 200 ng genomic DNA. Copy numbers were estimated to be the ~40.000 for MON810 and ~400 for the other three GMOs. A 1 C value was used of 2.725 for maize and 2.33 for cotton^44^ and a convension factor for 50% for hetero/hemizygous GMOs as recommended by the JRC^45^ to calculate in the 200 ng mixture: 35596 MON810 copies, 367 MON88017 and 367 MON89034 copies, and 429 MON15985 copies, which we rounded to 1 significant number. The following program was performed in a thermal cycler (iCycler, Bio-Rad): 2 min at 95 °C, 20 cycles of 1 min at 95 °C, 5 sec at 60 °C, ramp to 72 °C, over 1 min and 5 min at 72 °C.

### Column and bead purification

Column purification was performed (Qiaquick PCR Purification Kit, Qiagen) according to the manufacturer’s instructions, for removal of surplus primers and primer dimers. The linearly enriched fragments were eluted using 30 µl elution buffer provided with the kit. Streptavidin coated magnetic beads were used to select for the biotinylated enriched fragments from the genomic background. 15 µl Dynabeads MyOne Streptavidin C1 (Invitrogen) per sample were washed according to the manufacturer’s instructions and resuspended in 30 µl 2× B&W buffer (10 mM Tris-HCl (pH 7.5), 1 mM EDTA, 2 M NaCl). To immobilize the fragments, 30 µl column purified biotinylated enriched fragments were added to 30 µl washed beads and incubated for 30 min at 20 °C using a thermoshaker at 600 rpm. After immobilization, the DNA-bead complexes were washed for three times with 1× B&W buffer. Finally, samples were resuspended in 10 µl 10 mM Tris.

### Tailing

The tailing reaction consisted of 5× tailing buffer (5′ RACE System for Rapid Amplification of cDNA Ends, Invitrogen), 200 µM dCTPs and 10 µl purified sample. Water was added to a final volume of 24 µl. After mixing well, the mixture was incubated for 3 min at 94 °C and chilled for 1 min on ice. 1 μl of TdT (15 U/µl, Life Technologies) was added to each sample and incubated at 37 °C for 10 min. To heat inactivate the enzyme a last step of 10 min at 65 °C was performed then the samples were put on ice.

### Semi nested PCR

Expand Long Template PCR Buffer 1 (1×), dNTPs (200 µM; each, Invitrogen), semi-nested primer (0.4 µM) and Abridged Anchor Primer (0.4 µM, 5′ RACE System for Rapid Amplification of cDNA Ends, Invitrogen) (Supplementary Table S1) and *Taq* + *Tgo* polymerase blend (2.5 U, Expand Long Template PCR System, Roche) were combined with 10 µl of tailed product in a total volume of 50 µl. Amplification was achieved using the following cycling program: 94 °C for 2 min, 45 cycles of 95 °C for 10 sec and 59 °C for 6 min and a final extension of 72 °C for 5 min.

### Real-time polymerase chain reaction (qPCR)

Prior to q-PCR, reactions were diluted based on the amount of DNA put in the LE reaction. The following concentrations were used: 0.8 ng/µl for starting material (SM) and, 0.008 ng/µl for LE. qPCR reactions consisted of Diagenode (GMO-MM2X-A300), primers and probe (Supplementary Table S2) and 5 µl template in a total volume of 50 µl. Amplification was performed as follows: 2 min at 50 °C for Uracil N-Glycosylase (UNG) decontamination, denaturation for 10 min at 95 °C, 45 cycles of 15 s at 95 °C and 1 min at 60 °C using a MyiQ or CFX real-time PCR machine (Bio-Rad). Data-analysis was performed using CFX Manager Software Version 3.1 (Bio-Rad).

### PacBio analysis

A sequencing library for the PacBio platform was generated and sequenced at BaseClear BV (Leiden, The Netherlands). The ALF reactions were pooled, concentrated using 1.8 volume AMPureXP beads (Beckman Coulter Life Sciences) and subject to size-selection on agarose gel. The DNA fraction larger than 1 kb was isolated from gel, with a yield of 9.3 ng according to a measurement with the dsDNA high sensitivity Qubit assay (Life Technologies). The standard library preparation kits and protocols of the manufacturer (Pacific Biosciences) were used for library preparation, without DNA fragmentation, and despite the low input. The final library had a very low concentration of 0.1 ng/µl and was sequenced on one PacBio RSII SMRT cell. The run generated 2372 polymerase reads with an average subread length of 1031 bp. PacBio SMRT Portal’s CCS workflow was used to generate 411 CCS reads (deposited at NCBI’s Biosample database; accession number SAMN06461360).

### NGS data processing

CCS reads gained by PacBio RS II NGS sequencing were processed with Galaxy platform^46–48^. A pipeline, i.e. workflow (Supplementary pipeline online, https://github.com/RIKILT/ALF), was built using tools available in Galaxy (Supplementary Table S3). Three databases were built: event, CAF (Construct And Flanks) and element database (Supplementary Data S1, S2, S4 and S5). CAF database comprises of annotated reference sequences of MON810 (RIKILT20151130 joined JQ406879 and AY326434), MON89034 (DI362404), MON88017 (HV702026) and two partial MON15985 (EA135634 3′ flank, EA135633 5′ flank). Annotations were written in a General Feature Format (GFF) file (Supplementary Data S3).

## Electronic supplementary material

Supplementary Information

Supplementary pipeline

Supplementary spreadsheet
