# Author Correction: ALF: a strategy for identification of unauthorized GMOs in complex mixtures by a GW-NGS method and dedicated bioinformatics analysis

**DOI:** 10.1038/s41598-018-35950-y

**Published:** 2018-11-30

**Authors:** Alexandra Bogožalec Košir, Alfred J. Arulandhu, Marleen M. Voorhuijzen, Hongmei Xiao, Rico Hagelaar, Martijn Staats, Adalberto Costessi, Jana Žel, Esther J. Kok, Jeroen P. van Dijk

**Affiliations:** 1 Department of Biotechnology and Systems Biology, National Institute of Biology, Večna pot 111, SI-1000 Ljubljana, Slovenia; 2 Jožef Stefan International Postgraduate School, Jamova 39, SI-1000 Ljubljana, Slovenia; 3 RIKILT Wageningen UR, P.O. Box 230, 6700 AE Wageningen, The Netherlands; 4 Food Quality and Design Group, Wageningen University and Research, P.O. Box 8129, 6700 EV Wageningen, The Netherlands; 5 College of Food Science and Technology, Nanjing Agricultural University, Jiangsu, 210095 P. R. China; 6 BaseClear, Einsteinweg 5, 2333 CC Leiden, The Netherlands

Correction to: *Scientific Reports* 10.1038/s41598-017-14469-8, published online 26 October 2017

The supplementary Information, supplementary spreadsheet and supplementary pipeline files were omitted from the original version of this Article. This has been corrected in the HTML and PDF versions of the Article.

