# Supplementary material for: ALF: a strategy for identification of unauthorized GMOs in complex mixtures by a GW-NGS method and dedicated bioinformatics analysis
Source: Sci Rep. 2017 Oct 26;7:14155. doi: 10.1038/s41598-017-14469-8 (PMC5658351; doi:10.1038/s41598-017-14469-8)
Supplement: Supplementary file 1 — Supplementary Information [file 41598_2017_14469_MOESM1_ESM.pdf]

ALF: a strategy for identification of unauthorized GMOs in complex mixtures by a GW-NGS method and dedicated bioinformatics analysis

Alexandra Bogožalec Košir[1,2, ‡,]*, Alfred J. Arulandhu[3,4, ‡], Marleen M. Voorhuijzen[3], Hongmei Xiao[,5], Rico Hagelaar[3], Martijn Staats[3], Adalberto Costessi[6], Jana Žel[1], Esther J. Kok[3], Jeroen P. van Dijk[3,] *

[1]Department of Biotechnology and Systems Biology, National Institute of Biology, Večna pot 111, SI-1000 Ljubljana, Slovenia

[2]Jožef Stefan International Postgraduate School, Jamova 39, SI-1000 Ljubljana, Slovenia

[3]RIKILT Wageningen UR, P.O. Box 230, 6700 AE Wageningen, The Netherlands

[4]Food Quality and Design Group, Wageningen University and Research, P.O. Box 8129, 6700 EV Wageningen, The Netherlands.

[5]College of Food Science and Technology, Nanjing Agricultural University, Jiangsu, 210095, PR China

[6]BaseClear, Einsteinweg 5, 2333 CC Leiden, The Netherlands

‡ These authors contributed equally to this study

***Correspondence should be addressed to: Jeroen P. van Dijk**

RIKILT Wageningen UR,

P.O. Box 230,

6700 AE Wageningen,

The Netherlands

Tel: +31-(0)6-23918347

Email: jeroen.vandijk@wur.nl

Table of content

Table S1. Function and sequence of the primers used. 3

Table S2. Primer sequences and final concentrations used in the qPCR reaction used to determine size-dependent increase of specific target and loss of genomic background. 4

Table S3. Tools used in building of the Galaxy workflow for UGMO detection. 5

Table S4. BLAST output (first 5 hits) of the crCCS reads in the 'only unknown information bin'. 6

Data S1. Event database sequences. 8

Data S2. Reference sequences, CAF database. 9

Data S3. Annotations for reference sequences in CAF database. 17

Data S4. Element database - redundant, containing element sequences from all input GMOs. 19

Data S5. Element database , an un-redundant element database, containing only the longest of the element sequences. 33

Data S6. CCS reads with element orders tNOS – gap - *Npt II* – gap - p35S (133951, 45207), and p35S – gap – tNOS – gap – *uidA* (156962). p35S promoter is coloured yellow, tNOS terminator green *Npt II* gene red and *uidA* gene purple. 43

Figure S1. Formation of the circular consensus sequence (CCS) read. 45

Figure S2. Length distribution of the 411 CCS reads as generated by FastQC. 46

Figure S3. Building the workflow: in depth description. 47

Figure S4. Bowtie2 mapping of CCS reads to RIKILT20151130 sequence. 50

References 51

Table S1. Function and sequence of the primers used.

Oligo name	Oligo Sequence (5'-3')	Oligo function
**p35S down_biotin**	ATTGATGTGATATCTCCACTGACGT	Biotinylated primer used for linear enrichment
**p35S up_biotin**	CCTCTCCAAATGAAATGAACTTCCT	Biotinylated primer used for linear enrichment
**tNOS down_biotin**	GTCTTGCGATGATTATCATATAATTTCTG	Biotinylated primer used for linear enrichment
**tNOS up_biotin**	CGCTATATTTTGTTTTCTATCGCGT	Biotinylated primer used for linear enrichment
**p35S down**	AGGAAGTTCATTTCATTTGGAGAGG	Semi-nested primer p35S down
**p35S up**	GGTCTTGCGAAGGATAGTGGG	Semi-nested primer p35S up
**tNOS down**	AGATGGGTTTTTATGATTAGAG	Semi-nested primer tNOS down
**tNOS up**	CTCTAATCATAAAAACCCATCT	Semi-nested primer tNOS up
**AAP**	GGCCACGCGTCGACTAGTACGGGIIGGGIIGGGIIG	Universal primer complementary to poly dC tail

Table S2. Primer sequences and final concentrations used in the qPCR reaction used to determine size-dependent increase of specific target and loss of genomic background.

**Target**	**F/ R/P****	**Sequence**	**Final conc. in PCR [nM]**
HMG*	F	TTGGACTAGAAATCTCGTGCTGA	400
	R	GCTACATAGGGAGCCTTGTCCT	400
	P	5'-FAM-CAATCCACACAAACGCACGCGTA-TAMRA-3'	200
cp4-epsps	F	GCCTCGTGTCGGAAAACCCT	400
	R	TTCGTATCGGAGAGTTCGATCTTC	400
	P	5'-FAM-TGCCACGATGATCGCCACGAGCTTCC-TAMRA-3'	200
I-rAct1	F	TCGTCAGGCTTAGATGTGCTAGA	400
	R	CTGCATTTGTCACAAATCATGAA	400
	P	5'-FAM-TTTGTGGGTAGAATTTGAATCCCTCAGC-TAMRA-3'	200
Cry3Bb1	F	CCGCCCAGGACTCCATCG	400
	R	GAGGCACCCGAGGACAGG	400
	P	5'-FAM-CTGCCGCCTGAGACCACTGACGAGC-TAMRA-3'	200
tNOS	F	GTCTTGCGATGATTATCATATAATTTCTG	400
	R	CGCTATATTTTGTTTTCTATCGCGT	400
	P	5'-FAM-AGATGGGTTTTTATGATTAGAGTCCCGCAA-TAMRA-3'	200
Ctp2-cp4epsps	F	GGGATGACGTTAATTGGCTCTG	375
	R	GGCTGCTTGCACCGTGAAG	375
	P	5'-FAM-CACGCCGTGGAAACAGAAGACATGACC-TAMRA-3'	150
P35S	F	ATTGATGTGATATCTCCACTGACGT	400
	R	CCTCTCCAAATGAAATGAACTTCCT	400
	P	5'-FAM-CCCACTATCCTTCGCAAGACCCTTCCT-TAMRA-3'	200

* maize endogenous gene

** forward / reverse / probe

Table S3. Tools used in building of the Galaxy workflow for UGMO detection.

Tool	Description and Tool Shed URL
**Concatenate multiple datasets***	Combines multiple datasets tail-to-head, dataset collections can be merged to a single output file.<br>(https://toolshed.g2.bx.psu.edu/repository?repository_id=12213e550a6c4a72&changeset_revision=1e06fa2771f9)
**Convert characters**[3–5]	All delimiters of the selected type are converted into TAB characters.<br>(https://toolshed.g2.bx.psu.edu/repository/view_repository?changeset_revision=8a53d7f02ce4&id=84b1cc8e19e2e34c)
**Sharplabtool***<br>**Count**<br>**Filter**<br>**Reverse complement**	Tools count occurrences of each record, filter data on any column using simple expression and reverse complement of DNA/RNA sequences, respectively.<br>(https://toolshed.g2.bx.psu.edu/repository?repository_id=ecc93bc8f0382e9e&changeset_revision=c2a356708570)
**Cut columns**[3–5]	Allows selection of specific columns from the input dataset.<br>(https://toolshed.g2.bx.psu.edu/repository?repository_id=f11cf06cdcc13cfd&changeset_revision=842fab69c940)
**Cutadapt**[6] **1.8**	Finds and removes end sequences from high-throughput sequencing reads.<br>(https://toolshed.g2.bx.psu.edu/view/iuc/package_cutadapt_1_8/980a47047f5 https://pypi.python.org/pypi/cutadapt/1.8)
**FASTA-to-tabular**[3–5]	FASTA formatted sequences are converted to TAB-delimited format.<br>(https://toolshed.g2.bx.psu.edu/repository?repository_id=6fe870a3c7729070&changeset_revision=7e801ab2b70e)
**FASTX-Toolkit***<br>**FASTQ to FASTA**	Transforms FASTQ file format to a FASTA format.<br>(https://toolshed.g2.bx.psu.edu/repository?repository_id=190c5b4338681335&changeset_revision=186b8d913e6c)
**Filter sequences by ID***	Separates FASTA, FASTQ or SFF file on the basis of a specific ID present in a tabular file.<br>(https://toolshed.g2.bx.psu.edu/repository?repository_id=4963efc937542d6d)
**Bedtools**[7]	A group of flexible tools for genome arithmetic and NGS analysis.<br>(https://toolshed.g2.bx.psu.edu/repository?repository_id=1ec48b84b33d36d8&changeset_revision=41bba3e648d1)
**Standalone NCBI BLAST+ tools**[89]	Allows performing of BLAST on a local as well as public database.<br>(https://toolshed.g2.bx.psu.edu/repository?repository_id=1d92ebdf7e8d466c&changeset_revision=7f3c448e119b)
**Tabular-to-FASTA**[3–5]	TAB-delimited format file is converted to FASTA formatted sequence.<br>(https://toolshed.g2.bx.psu.edu/repository?repository_id=0b58502f66340f5d&changeset_revision=0b4e36026794)
**Text processing**<br>**Text reformatting - awk**<br>**Text transformation - sed**	Text processing tools using awk and sed programming language, respectively.<br>(https://toolshed.g2.bx.psu.edu/repository?repository_id=2593fd36ae8011aa&changeset_revision=616efa22d193)
**UPARSE**[10]	Cluster OTUs - OTU clustering using the UPARSE-OTU algorithm.<br>(https://toolshed.g2.bx.psu.edu/repository?repository_id=7edb1c97d9eb2336&changeset_revision=5d05b34a5fdf)
**USEARCH**[11]	Usearche_dereplication – eliminates replicate sequences.<br>(https://toolshed.g2.bx.psu.edu/repository?repository_id=7666a94ede912d3f&changeset_revision=88fc52f1c5db<br>USEARCH – search and clustering algorithms for sequence analysis.<br>(https://toolshed.g2.bx.psu.edu/repository?repository_id=13df57e58dc7e075&changeset_revision=6f967c3a3f7b)

* Available in the Galaxy[3–5] ToolShed.

Table S4. BLAST output (first 5 hits) of the crCCS reads in the 'only unknown information bin'.

crCCS/crCCS length	Description	Query start-finish	Subject start-finish	Most specific subject annotation including the region of homology	Query cover [%]	E value	Ident [%]	Accession
**120091/895**	Zea mays subsp. mays genotype CMS-S mitochondrion, complete genome	11-895	92301-91416	1..557162/organism="Zea mays subsp. mays"/organelle="mitochondrion"/mol_type="genomic DNA"	98	0.0	99	DQ490951.2
	Zea mays subsp. parviglumis mitochondrion, complete genome	11-895	396277-397162	387242..442430/note="first copy of 55 kb R55"/rpt_type=inverted	98	0.0	99	DQ645539.1
	Zea mays subsp. mays genotype CMS-C mitochondrion, complete genome	11-895	134589-135474	125546..230101/note="first copy of 105 kb R105"/rpt_type=inverted	98	0.0	99	DQ645536.1
	Zea mays subsp. mays genotype CMS-T mitochondrion, complete genome	11-895	83514-84399	1..535825/organism="Zea mays subsp. mays"/organelle="mitochondrion"/mol_type="genomic DNA"	98	0.0	99	DQ490953.1
	Zea mays subsp. mays genotype male-fertile NA mitochondrion, complete genome	11-895	182577-181692	72199..191613/note="first copy of 120 kb R120"/rpt_type=direct	98	0.0	99	DQ490952.1
**106520/971**	Sequence 55213 from Patent WO2014036048	66-549	484-1	1..484/organism="Zea mays"/mol_type="unassigned DNA"	49	0.0	99	JC804838.1
	Sequence 58344 from Patent WO2014036048	436-715	436-1	1..653/organism="Zea mays"/mol_type="unassigned DNA"	45	6e-177	93	JC807969.1
	Sequence 11881 from Patent WO2014036048	164-354	228-39	1..577/organism="Zea mays"/mol_type="unassigned DNA"	19	2e-68	92	JC761506.1
	Sequence 52740 from patent US 7569389	870-960	177-267	1..689/organism="unknown"/mol_type="genomic DNA"	9	2e-28	93	GP689587.1
	Sequence 62396 from patent US 7569389	870-960	272-362	1..912/organism="unknown"/mol_type="genomic DNA"	9	4e-25	91	GP694415.1
**43434/120**	Compositions and methods for the therapy and diagnosis of ovarian cancer	27-99	233-161	1..256/organism="Homo sapiens"/mol_type="unassigned DNA"	60	7e-19	90	DL059073.1
	Compositions and methods for the therapy and diagnosis of ovarian cancer	27-99	5-77	1..246/organism="Homo sapiens"/mol_type="unassigned DNA"	60	7e-19	90	DL058560.1
	Sequence 9347 from Patent WO0192581	27-99	233-161	1..256/organism="Homo sapiens"/mol_type="unassigned DNA"	60	7e-19	90	CQ466569.1
	Sequence 8834 from Patent WO0192581	27-99	5-77	1..246/organism="Homo sapiens"/mol_type="unassigned DNA"	60	7e-19	90	CQ466056.1
	Compositions and methods for the therapy and diagnosis of ovarian cancer	38-99	56-117	1..298/organism="Homo sapiens"/mol_type="unassigned DNA"	51	3e-18	95	DL058704.1

Data S1. Event database sequences.

```
>MON810
TCGAAGGACGAAGGACTCTAACGTTTAACATCCTTTGCCATTGCCCAGCTATCTGTCACTTTATTGTGAAGATAG
TGGAAAAGGAAGGTGGC

>MON89034
TTCTCCATATTGACCATCATACTCATTGCATCCCCGGAAATTATGTTTTTTTAAAAACCACGGTATTATAGATAC
CG

>MON88017
GAGCAGGACCTGCAGAAGCTAGCTTGATGGGGATCAGATTGTCGTTTCCCGCCTTCAGTTTAAACAGAGTCGGGT
TTGGATGGTCAACTCCGGCA

>MON15985
GTTACTAGATCGGGGATATCCCCGGGGCGGCCGCTCTAGAACTAGTGGATCTGCACTGAAATCCCATCCATTTAG
CAACCTT
```

Data S2. Reference sequences, CAF database.

```
>RIKILT20151130 MON810_joined_JQ406879_and_AY326434
TGAGACATCTTCGGAAATGCTGACAAAAGTGCTCTCAAAGCCGAAGCTTAAAAAATCTAAAAAGCCAAGC
AAATGTTGGTGCGCAAGAGCTGAAAATTGAGTAAGAAAGAGCAGATGGCAAGAAAGCGTGCCAAATGAGC
TCGTGGTGCGCTCTTATTTATACGCCTAGTGCGCTGAAAACTGGAAGGGCCCGCTTGTCAGTGACTGTTG
CTATTCTAGCAAAGGAAAGGTGTTTTTTCGGACCTTCGGCTTAGGGCCTTCGTCCATATCGCAATCTAAA
TTTATCATTCTAACAAATTAATATTACGAGGGGCTACTGTTGGTGGCCTTCGGCTTCTGAAGGTCCTCAA
AAACATGATTTAACAAAGTTTCTGGAGTATGATGCATGAACAGGTATCTTCGGACTTGAGTTAAAACCAC
AGTGTGAAGAAGCACAAAAGGAATACGAAGGATGTCGCGGAGCCGAAGCTGTGCGCAGAAGAGCTTCGAG
ATAATAGCAGAAAAGGAAACCGACTTAAAGATGAAAAGGCTATTTAGACCTCGACGGATTACTATAGAGT
TATTAGCAAATGTAGAGGGCATGGGTGTAATTTCATATGGGCTGCGTCTCGTGCCTATAAATAGATGAAC
AGTGTTTCCGTACTGTTCGCGCTGACTTGGCATTTGCTTTTGCGCCACGCTTATACTTTTACCTTCTTTC
AAGCCGAAGGTACATCTGTAATTTGATATCATTTCTATTCTTCCATGATAATAAAATAGAAATAAGTTGA
TTATAATATATAATTGTTTATGTTATCTCTTATACTTCATATGATTCCTTCTTCATTATTATATCTTGTG
CTGATGAAGGTATGTCCTTCATAACCTTCGCCCGAAAATCATTATATCCCAAGGGAAATAATGCTTCGAA
GGACGAAGGACTCTAACGTTTAACATCCTTTGCCATTGCCCAGCTATCTGTCACTTTATTGTGAAGATAG
TGGAAAAGGAAGGTGGCTCCTACAAATGCCATCATTGCGATAAAGGAAAGGCCATCGTTGAAGATGCCTC
TGCCGACAGTGGTCCCAAAGATGGACCCCCACCCACGAGGAGCATCGTGGAAAAAGAAGACGTTCCAACC
ACGTCTTCAAAGCAAGTGGATTGATGTGATATCTCCACTGACGTAAGGGATGACGCACAATCCCACTATC
CTTCGCAAGACCCTTCCTCTATATAAGGAAGTTCATTTCATTTGGAGAGGACACGCTGACAAGCTGACTC
TAGCAGATCTACCGTCTTCGGTACGCGCTCACTCCGCCCTCTGCCTTTGTTACTGCCACGTTTCTCTGAA
TGCTCTCTTGTGTGGTGATTGCTGAGAGTGGTTTAGCTGGATCTAGAATTACACTCTGAAATCGTGTTCT
GCCTGTGCTGATTACTTGCCGTCCTTTGTAGCAGCAAAATATAGGGACATGGTAGTACGAAACGAAGATA
GAACCTACACAGCAATACGAGAAATGTGTAATTTGGTGCTTAGCGGTATTTATTTAAGCACATGTTGGTG
TTATAGGGCACTTGGATTCAGAAGTTTGCTGTTAATTTAGGCACAGGCTTCATACTACATGGGTCAATAG
TATAGGGATTCATATTATAGGCGATACTATAATAATTTGTTCGTCTGCAGAGCTTATTATTTGCCAAAAT
TAGATATTCCTATTCTGTTTTTGTTTGTGTGCTGTTAAATTGTTAACGCCTGAAGGAATAAATATAAATG
ACGAAATTTTGATGTTTATCTCTGCTCCTTTATTGTGACCATAAGTCAAGATCAGATGCACTTGTTTTAA
ATATTGTTGTCTGAAGAAATAAGTACTGACAGTATTTTGATGCATTGATCTGCTTGTTTGTTGTAACAAA
ATTTAAAAATAAAGAGTTTCCTTTTTGTTGCTCTCCTTACCTCCTGATGGTATCTAGTATCTACCAACTG
ACACTATATTGCTTCTCTTTACATACGTATCTTGCTCGATGCCTTCTCCCTAGTGTTGACCAGTGTTACT
CACATAGTCTTTGCTCATTTCATTGTAATGCAGATACCAAGCGGCCATGGACAACAACCCAAACATCAAC
GAGTGCATCCCGTACAACTGCCTCAGCAACCCTGAGGTCGAGGTGCTCGGCGGTGAGCGCATCGAGACCG
GTTACACCCCCATCGACATCTCCCTCTCCCTCACGCAGTTCCTGCTCAGCGAGTTCGTGCCAGGCGCTGG
CTTCGTCCTGGGCCTCGTGGACATCATCTGGGGCATCTTTGGCCCCTCCCAGTGGGACGCCTTCCTGGTG
CAAATCGAGCAGCTCATCAACCAGAGGATCGAGGAGTTCGCCAGGAACCAGGCCATCAGCCGCCTGGAGG
GCCTCAGCAACCTCTACCAAATCTACGCTGAGAGCTTCCGCGAGTGGGAGGCCGACCCCACTAACCCAGC
TCTCCGCGAGGAGATGCGCATCCAGTTCAACGACATGAACAGCGCCCTGACCACCGCCATCCCACTCTTC
GCCGTCCAGAACTACCAAGTCCCGCTCCTGTCCGTGTACGTCCAGGCCGCCAACCTGCACCTCAGCGTGC
TGAGGGACGTCAGCGTGTTTGGCCAGAGGTGGGGCTTCGACGCCGCCACCATCAACAGCCGCTACAACGA
CCTCACCAGGCTGATCGGCAACTACACCGACCACGCTGTCCGCTGGTACAACACTGGCCTGGAGCGCGTC
TGGGGCCCTGATTCTAGAGACTGGATTCGCTACAACCAGTTCAGGCGCGAGCTGACCCTCACCGTCCTGG
ACATTGTGTCCCTCTTCCCGAACTACGACTCCCGCACCTACCCGATCCGCACCGTGTCCCAACTGACCCG
CGAAATCTACACCAACCCCGTCCTGGAGAACTTCGACGGTAGCTTCAGGGGCAGCGCCCAGGGCATCGAG
GGCTCCATCAGGAGCCCACACCTGATGGACATCCTCAACAGCATCACTATCTACACCGATGCCCACCGCG
GCGAGTACTACTGGTCCGGCCACCAGATCATGGCCTCCCCGGTCGGCTTCAGCGGCCCCGAGTTTACCTT
TCCTCTCTACGGCACGATGGGCAACGCCGCTCCACAACAACGCATCGTCGCTCAGCTGGGCCAGGGCGTC
TACCGCACCCTGAGCTCCACCCTGTACCGCAGGCCCTTCAACATCGGTATCAACAACCAGCAGCTGTCCG
TCCTGGATGGCACTGAGTTCGCCTACGGCACCTCCTCCAACCTGCCCTCCGCTGTCTACCGCAAGAGCGG
CACGGTGGATTCCCTGGACGAGATCCCACCACAGAACAACAATGTGCCCCCCAGGCAGGGTTTTTCCCAC
AGGCTCAGCCACGTGTCCATGTTCCGCTCCGGCTTCAGCAACTCGTCCGTGAGCATCATCAGAGCTCCTA
```

TGTTCTCCTGGATTCATCGCAGCGCGGAGTTCAACAATATCATTCCGTCCTCCCAAATCACCCAAATCCC
CCTCACCAAGTCCACCAACCTGGGCAGCGGCACCTCCGTGGTGAAGGGCCCAGGCTTCACGGGCGGCGAC
ATCCTGCGCAGGACCTCCCCGGGCCAGATCAGCACCCTCCGCGTCAACATCACCGCTCCCCTGTCCCAGA
GGTACCGCGTCAGGATTCGCTACGCTAGCACCACCAACCTGCAATTCCACACCTCCATCGACGGCAGGCC
GATCAATCAGGGTAACTTCTCCGCCACCATGTCCAGCGGCAGCAACCTCCAATCCGGCAGCTTCCGCACC
GTGGGTTTCACCACCCCCTTCAACTTCTCCAACGGCTCCAGCGTTTTCACCCTGAGCGCCCACGTGTTCA
ATTCCGGCAATGAGGTGTACATTGACCGCATTGAGTTCGTGCCAGCCGAGGTCACCTTCGAAGCCGAGTA
CGACCTGGAGAGAGCCCAGAAGGCTGTCAATGAGCTCTTCACGTCCAGCAATCAGATCGGCCTGAAGACC
GACGTCACTGACTACCACATCGACCAAGTCTCCAACCTCGTGGAGTGCCTCTCCGATGAGTTCTGCCTCG
ACGAGAAGAAGGAGCTGTCCGAGAAGGTGAAGCATGCCAAGCGTCTCAGCGACGAGAGGAATCTCCTCCA
GGACCCCAATTTCCGCGGCATCAACAGGCAGCTCGACCGCGGCTGGCGCGGCAGCACCGACATCACGATC
CAGGGCGGCGACGATGTGTTCAAGGAGAACTACGTGACTCTCCTGGGCACTTTCGACGAGTGCTACCCTA
CCTACTTGTACCAGAAGATCGATGAGTCCAAGCTCAAGGCTTACACTCGCTACCAGCTCCGCGGCTACAT
CGAAGACAGCCAAGACCTCGAGATTTACCTGATCCGCTACAACGCCAAGCACGAGACCGTCAACGTGCCC
GGTACTGGTTCCCTCTGGCCGCTGAGCGCCCCCAGCCCGATCGGCAAGTGTGCCCACCACAGCCACCACT
TCTCCTTGGACATCGATGTGGGCTGCACCGACCTGAACGAGGACTTTCGGTAGCCTTCTTTCATTTCCGA
ATTTGCTTGCGAGCAGTCAGGTCCTTTTGATTCATCTGAGTTTGGCTTTACAATAGCTTTTCCTTTTCCT
TTGGCAGTACTAGTGCTTTCATCATGAGAATCCTTCTTAGATGTAAGACCACCTGCAGCAGATGACTTTG
ATCTTGTTGTTGGGCGCCGACCAGATTGAGCCATTGCAGCTGTTAATGATGCACCAGCCGTGGTGCCAGG
AACCCCAGATTCAGAATTATTACCAGATGGAATTATAGGCTTCGATGCAACCTCACTGCGTTGAACTCTA
GGCCAAAGGAATTCTTCAACAGATGCAAGACTAGCAAATGGGTCGATAAGCACAATATTTGATGAATAAT
CCCGAAGTGATTTTTCGCCATGAGCTCGGGAAAGACGAAGCTTGAAGGGTTGAGCCAGAGCACTAAGACC
TGAAGTCAGACGAGACCCTCCAATACCAATCCTACTAGACTGGCTGAGCACAACAGGGAAACGTTCCAGC
GAAGACAATGCACTTTGCAGTTTCTGAACCAACAGTGCCATGGGAGTTTCATCTCTCTCGACCAATCGAA
TTTCCGCGGGCC

>DI362404_MON89034
AATATTTAAAAATGGAAGTAATACTATATTAAAATGATTCATGTGGAACTCCTGCGCTTCTTTTTGAAGT
TTCAAAAGGGAGCTTTCAGGGTCGCTTAGAGTTTGTTTGGTTGGAAATACAAGCGAAAAGAGAGCTAATG
AGGGGGACATCCATATTTTCTATGGTGTTTGAATAAGAGTCACGCGGGAATAAGATGAACACCGAAACAA
TTTTTTTGTAGCTACGTGGTTCCAAAAAATCGAGTAGACGGTGTCGCTTCCACCTCATACTACTTCAACC
TCAAACCACACATCCTTACGCGCCCGCCGGTGTCTCCGTCGTCTCAAGTTCTCAACATACCTACACATGT
AACCACTACCGGTCTTTGTCATGTTTCGAAAGAAGATTACAGGTCCTCTAGAAGAGAGGACGCGGGGTGG
CGAGAAAGCTGGGGAAGAAAAAGGCTAGTACATATGATTGGTCTGTGAACCTGTGAGGTGGGTAGGTAGG
TAGGTGGAGATTTTTGTTAACTGGTGTTGTTGACGGACTCGAACGGGGCCGGGCGTGTGGTGTGGCTAGC
TGTGGTGGTTTGCTCGCCAGCCAGCCAGCCACACATCAGCGAGCATGCAGAGCTTAAGCATGTATGTACG
GATCGGCTTGCTTAGCGGTTAAGGGTGTGTTTGGTTTGGCTTTTGGCTTTGGCTTTTGCCCCCTAAAAGC
CAAAAGCCAACCAAGGGTGTATTTGGTTTGACTTTTGGCTTTTGGCTTTTGTCCCCTAAAAGCCAAAAGC
CAAACAAAGGGTTAGATCTAGGAAGCAGCTTTTTCTAAAAGCTGGCTTTCTCACAGTGCAAATCTGAAAG
CACCCCTGAACCTGCTTTTAGTGGCTTTTCGAATGGAACTGTGAAAACATATATCGAAGAACTTTTAACG
ACTTTTAGTGGTTTCCACCAAACAGTTTAGCTTTTTAACGGCTTACAGCCTACAACAGCTTTTTCCACAG
CTCACAGCCCACAGCAACTTTTTTCACAGCCACAGCCCAACCAAACAGACCCCAAAGGGCTGAATCCAGG
AAGCAGCTTTTTCTAAAAGCCGACTTTCTCGTAGTGTAAAACTGAAAACACCCCTGGACCTGCTTTTAGT
GGCTTTTGGATGGAACTGTGAAAACATATATCGAAGAACTTTTAACGACTTTTAGTGGTTTCCACCAAAC
GATTTCTAGCTTTTTAACAGCACACAGCCTACAACAGCTTTTTTCACAGCTCACAGCCCACAACAACTTT
TTCTACAGCCACAACCCAACCAAACGGACCCTAAGGCGGCCGAGCGAGCGCAAAGCGTCGTCAGCTTTGA
TTGCCATGCCATCTCCTGCTCCACTTGTCTCTCTGGCCGTCGTCAGCCACCATCCAACAAGGCCGGTGCT
ACTGGCGGCTCCTAGGTAGACGACGACGACGACGACACCTCCACCGTTCGCCGCCGTCCACTCACCAATC
AACACGGAACGCCCAAAACACACACACACGCACGCTGGGGAGGGAAAAAAAGGCAGAGACATATGCGTGC
GTCGCTGCATTATTGTACGCGATCGAATGGCATCTCTCTCACTCTCTCTCCTCCCTTTATTAATCTGGTA
CTGGCTAGCTGGTCCGGCGACACCGACGTGTCAGCTCCGTCGCGCGCGTCCGTCCGTCCGCTGGAGCGGA
CACGGACCGCGGCGTCTGTCGATCGCCGGCTCGCCGCAGCGCAGCTACCTAGCACGCTCACGCATGCTAC
ACTGCCTACACGCACACGGCCGGCCCAAAAGCGTTCCCTGCCGCCTGCCGGCCGGCTTTTTTATTATTAT

TGGAACATGAGGCTATTTCTCCTCCCACACGGGCTACGACGTGAGCACGAGTACTGGGATCCCCGGATCC
GCCCTCTCTGTCCCTGCTGCTACTCCAGCCACTGAAATGTTGTCAGATGAAACAGCAGAGCCGATCTCCG
CACGGAAACCCATGCACGGCCATTCAAATTCAGGTGCCCACGTACGTCAGGGTGCTGCTGCTACTACTAT
CAAGCCAATAAAAGGATGGTAATGAGTATGATGGATCAGCAATGAGTATGATGGTCAATATGGAGAAAAA
GAAAGAGTAATTACCAATTTTTTTTCAATTCAAAAATGTAGATGTCCGCAGCGTTATTATAAAATGAAAG
TACATTTTGATAAAACGACAAATTACGATCCGTCGTATTTATAGGCGAAAGCAATAAACAAATTATTCTA
ATTCGGAAATCTTTATTTCGACGTGTCTACATTCACGTCCAAATGGGGGCTTAGATGAGAAACTTCACGA
TTTGGCGCGCCAAAGCTTGGTCGAGTGGAAGCTAGCTTTCCGATCCTACCTGTCACTTCATCAAAAGGAC
AGTAGAAAAGGAAGGTGGCTCCTACAAATGCCATCATTGCGATAAAGGAAAGGCCATCGTTGAAGATGCC
TCTGCCGACAGTGGTCCCAAAGATGGACCCCCACCCACGAGGAGCATCGTGGAAAAAGAAGACGTTCCAA
CCACGTCTTCAAAGCAAGTGGATTGATGTGATATCTCCACTGACGTAAGGGATGACGCACAATCCCACTA
TCCTTCGCAAGACCCTTCCTCTATATAAGGAAGTTCATTTCATTTGGAGAGGACACGCTGACAAGCTGAC
TCTAGCAGATCCTCTAGAACCATCTTCCACACACTCAAGCCACACTATTGGAGAACACACAGGGACAACA
CACCATAAGATCCAAGGGAGGCCTCCGCCGCCGCCGGTAACCACCCCGCCCCTCTCCTCTTTCTTTCTCC
GTTTTTTTTTCCGTCTCGGTCTCGATCTTTGGCCTTGGTAGTTTGGGTGGGCGAGAGGCGGCTTCGTGCG
CGCCCAGATCGGTGCGCGGGAGGGGCGGGATCTCGCGGCTGGGGCTCTCGCCGGCGTGGATCCGGCCCGG
ATCTCGCGGGGAATGGGGCTCTCGGATGTAGATCTGCGATCCGCCGTTGTTGGGGGAGATGATGGGGGGT
TTAAAATTTCCGCCGTGCTAAACAAGATCAGGAAGAGGGGAAAAGGGCACTATGGTTTATATTTTTATAT
ATTTCTGCTGCTTCGTCAGGCTTAGATGTGCTAGATCTTTCTTTCTTCTTTTTGTGGGTAGAATTTGAAT
CCCTCAGCATTGTTCATCGGTAGTTTTTCTTTTCATGATTTGTGACAAATGCAGCCTCGTGCGGAGCTTT
TTTGTAGGTAGAAGTGATCAACCATGGACAACAACCCAAACATCAACGAGTGCATCCCGTACAACTGCCT
CAGCAACCCTGAGGTCGAGGTGCTCGGCGGTGAGCGCATCGAGACCGGTTACACCCCCATCGACATCTCC
CTCTCCCTCACGCAGTTCCTGCTCAGCGAGTTCGTGCCAGGCGCTGGCTTCGTCCTGGGCCTCGTGGACA
TCATCTGGGGCATCTTTGGCCCCTCCCAGTGGGACGCCTTCCTGGTGCAAATCGAGCAGCTCATCAACCA
GAGGATCGAGGAGTTCGCCAGGAACCAGGCCATCAGCCGCCTGGAGGGCCTCAGCAACCTCTACCAAATC
TACGCTGAGAGCTTCCGCGAGTGGGAGGCCGACCCCACTAACCCAGCTCTCCGCGAGGAGATGCGCATCC
AGTTCAACGACATGAACAGCGCCCTGACCACCGCCATCCCACTCTTCGCCGTCCAGAACTACCAAGTCCC
GCTCCTGTCCGTGTACGTCCAGGCCGCCAACCTGCACCTCAGCGTGCTGAGGGACGTCAGCGTGTTTGGC
CAGAGGTGGGGCTTCGACGCCGCCACCATCAACAGCCGCTACAACGACCTCACCAGGCTGATCGGCAACT
ACACCGACCACGCTGTCCGCTGGTACAACACTGGCCGTCCTGGACATTGTGTCCCTCTTCCCGAACTACG
ACTCCCGCACCTACCCGATCCGCACCGTGTCCCAACTGACCCGCGAAATCTACACCAACCCCGTCCTGGA
GAACTTCGACGGTAGCTTCAGGGGCAGCGCCCAGGGCATCGAGGGCTCCATCAGGAGCCCACACCTGATG
GACATCCTCAACAGCATCACTATCTACACCGATGCCCACCGCGGCGAGTACTACTGGTCCGGCCACCAGA
TCATGGCCTCCCCGGTCGGCTTCAGCGGCCCCGAGTTTACCTTTCCTCTCTACGGCACGATGGGCAACGC
CGCTCCACAACAACGCATCGTCGCTCAGCTGGGCCAGGGCGTCTACCGCACCCTGAGCTCCACCCTGTAC
CGCAGGCCCTTCAACATCGGTATCAACAACCAGCAGCTGTCCGTCCTGGATGGCACTGAGTTCGCCTACG
GCACCTCCTCCAACCTGCCCTCCGCTGTCTACCGCAAGAGCGGCACGGTGGATTCCCTGGACGAGATCCC
ACCACAGAACAACAATGTGCCCCCCAGGCAGGGTTTTTCCCACAGGCTCAGCCACGTGTCCATGTTCCGC
TCCGGCTTCAGCAACTCGTCCGTGAGCATCATCAGAGCTCCTATGTTCTCTTGGATACACCGTAGTGCTG
AGTTCAACAACATCATTGCATCCGACAGCATTACTCAAATACCCTTGGTGAAAGCACATACACTTCAGTC
AGGTACTACTGTTGTCAGAGGTCCAGGGTTTACAGGAGGAGACATTCTTCGTCGCACAAGTGGAGGACCC
TTTGCTTACACTATTGTTAACATCAATGGCCAATTGCCCCAAAGGTATCGTGCAAGAATCCGCTATGCCT
CTACTACAAATCTCAGGATCTACGTGACTGTTGCAGGTGAAAGGATCTTTGCTGGTCAGTTCAACAAGAC
TATGGATACCGGTGACCCTTTGACATTCCAATCTTTTAGCTACGCAACTATCAACACAGCTTTTACATTC
CCAATGAGCCAGAGTAGCTTCACAGTAGGTGCTGACACTTTCAGCTCAGGGAATGAAGTTTACATCGACA
GGTTTGAATTGATTCCAGTTACTGCAACCCTCGAGGCTGAGTACAACCTTGAGAGAGCCCAGAAGGCTGT
GAACGCCCTCTTTACCTCCACCAATCAGCTTGGCTTGAAAACTAACGTTACTGACTATCACATTGACCAA
GTGTCCAACTTGGTCACCTACCTTAGCGATGAGTTCTGCCTCGACGAGAAGCGTGAACTCTCCGAGAAAG
TTAAACACGCCAAGCGTCTCAGCGACGAGAGGAATCTCTTGCAAGACTCCAACTTCAAAGACATCAACAG
GCAGCCAGAACGTGGTTGGGGTGGAAGCACCGGGATCACCATCCAAGGAGGCGACGATGTGTTCAAGGAG
AACTACGTCACCCTCTCCGGAACTTTCGACGAGTGCTACCCTACCTACTTGTACCAGAAGATCGATGAGT
CCAAACTCAAAGCCTTCACCAGGTATCAACTTAGAGGCTACATCGAAGACAGCCAAGACCTTGAAATCTA
CTCGATCAGGTACAATGCCAAGCACGAGACCGTGAATGTCCCAGGTACTGGTTCCCTCTGGCCACTTTCT
GCCCAATCTCCCATTGGGAAGTGTGGAGAGCCTAACAGATGCGCTCCACACCTTGAGTGGAATCCTGACT
TGGACTGCTCCTGCAGGGATGGCGAGAAGTGTGCCCACCATTCTCATCACTTCTCCTTGGACATCGATGT

GGGATGTACTGACCTGAATGAGGACCTCGGAGTCTGGGTCATCTTCAAGATCAAGACCCAAGACGGACAC
GCAAGACTTGGCAACCTTGAGTTTCTCGAAGAGAAACCATTGGTCGGTGAAGCTCTCGCTCGTGTGAAGA
GAGCAGAGAAGAAGTGGAGGGACAAACGTGAGAAACTCGAATGGGAAACTAACATCGTTTACAAGGAGGC
CAAAGAGTCCGTGGATGCTTTGTTCGTGAACTCCCAATATGATCAGTTGCAAGCCGACACCAACATCGCC
ATGATCCACGCCGCAGACAAACGTGTGCACAGCATTCGTGAGGCTTACTTGCCTGAGTTGTCCGTGATCC
CTGGTGTGAACGCTGCCATCTTCGAGGAACTTGAGGGACGTATCTTTACCGCATTCTCCTTGTACGATGC
CAGAAACGTCATCAAGAACGGTGACTTCAACAATGGCCTCAGCTGCTGGAATGTGAAAGGTCATGTGGAC
GTGGAGGAACAGAACAATCAGCGTTCCGTCCTGGTTGTGCCTGAGTGGGAAGCTGAAGTGTCCCAAGAGG
TTAGAGTCTGTCCAGGTAGAGGCTACATTCTCCGTGTGACCGCTTACAAGGAGGGATACGGTGAGGGTTG
CGTGACCATCCACGAGATCGAGAACAACACCGACGAGCTTAAGTTCTCCAACTGCGTCGAGGAAGAAATC
TATCCCAACAACACCGTTACTTGCAACGACTACACTGTGAATCAGGAAGAGTACGGAGGTGCCTACACTA
GCCGTAACAGAGGTTACAACGAAGCTCCTTCCGTTCCTGCTGACTATGCCTCCGTGTACGAGGAGAAATC
CTACACAGATGGCAGACGTGAGAACCCTTGCGAGTTCAACAGAGGTTACAGGGACTACACACCACTTCCA
GTTGGCTATGTTACCAAGGAGCTTGAGTACTTTCCTGAGACCGACAAAGTGTGGATCGAGATCGGTGAAA
CCGAGGGAACCTTCATCGTGGACAGCGTGGAGCTTCTCTTGATGGAGGAATAATGAGATCTATCGATTCT
AGAAGGCCTGAATTCTGCATGCGTTTGGACGTATGCTCATTCAGGTTGGAGCCAATTTGGTTGATGTGTG
TGCGAGTTCTTGCGAGTCTGATGAGACATCTCTGTATTGTGTTTCTTTCCCCAGTGTTTTCTGTACTTGT
GTAATCGGCTAATCGCCAACAGATTCGGCGATGAATAAATGAGAAATAAATTGTTCTGATTTTGAGTGCA
AAAAAAAAGGAATTAGATCTGTGTGTGTTTTTTGGATCCCCGGGGCGGCCGCGTTAACAAGCTTGAGCTC
AGGATTTAGCAGCATTCCAGATTGGGTTCAATCAACAAGGTACGAGCCATATCACTTTATTCAAATTGGT
ATCGCCAAAACCAAGAAGGAACTCCCATCCTCAAAGGTTTGTAAGGAAGAATTCTCAGTCCAAAGCCTCA
ACAAGGTCAGGGTACAGAGTCTCCAAACCATTAGCCAAAAGCTACAGGAGATCAATGAAGAATCTTCAAT
CAAAGTAAACTACTGTTCCAGCACATGCATCATGGTCAGTAAGTTTCAGAAAAAGACATCCACCGAAGAC
TTAAAGTTAGTGGGCATCTTTGAAAGTAATCTTGTCAACATCGAGCAGCTGGCTTGTGGGGACCAGACAA
AAAAGGAATGGTGCAGAATTGTTAGGCGCACCTACCAAAAGCATCTTTGCCTTTATTGCAAAGATAAAGC
AGATTCCTCTAGTACAAGTGGGGAACAAAATAACGTGGAAAAGAGCTGTCCTGACAGCCCACTCACTAAT
GCGTATGACGAACGCAGTGACGACCACAAAAGAATTCCCTCTATATAAGAAGGCATTCATTCCCATTTGA
AGGATCATCAGATACTCAACCAATCCTTCTAGGATCTACCGTCTTCGGTACGCGCTCACTCCGCCCTCTG
CCTTTGTTACTGCCACGTTTCTCTGAATGCTCTCTTGTGTGGTGATTGCTGAGAGTGGTTTAGCTGGATC
TAGAATTACACTCTGAAATCGTGTTCTGCCTGTGCTGATTACTTGCCGTCCTTTGTAGCAGCAAAATATA
GGGACATGGTAGTACGAAACGAAGATAGAACCTACACAGCAATACGAGAAATGTGTAATTTGGTGCTTAG
CGGTATTTATTTAAGCACATGTTGGTGTTATAGGGCACTTGGATTCAGAAGTTTGCTGTTAATTTAGGCA
CAGGCTTCATACTACATGGGTCAATAGTATAGGGATTCATATTATAGGCGATACTATAATAATTTGTTCG
TCTGCAGAGCTTATTATTTGCCAAAATTAGATATTCCTATTCTGTTTTTGTTTGTGTGCTGTTAAATTGT
TAACGCCTGAAGGAATAAATATAAATGACGAAATTTTGATGTTTATCTCTGCTCCTTTATTGTGACCATA
AGTCAAGATCAGATGCACTTGTTTTAAATATTGTTGTCTGAAGAAATAAGTACTGACAGTATTTTGATGC
ATTGATCTGCTTGTTTGTTGTAACAAAATTTAAAAATAAAGAGTTTCCTTTTTGTTGCTCTCCTTACCTC
CTGATGGTATCTAGTATCTACCAACTGACACTATATTGCTTCTCTTTACATACGTATCTTGCTCGATGCC
TTCTCCCTAGTGTTGACCAGTGTTACTCACATAGTCTTTGCTCATTTCATTGTAATGCAGATACCAAGCG
GCCTCTAGAGGATCAGCATGGCGCCCACCGTGATGATGGCCTCGTCGGCCACCGCCGTCGCTCCGTTCCA
GGGGCTCAAGTCCACCGCCAGCCTCCCCGTCGCCCGCCGCTCCTCCAGAAGCCTCGGCAACGTCAGCAAC
GGCGGAAGGATCCGGTGCATGCAGGTAACAAATGCATCCTAGCTAGTAGTTCTTTGCATTGCAGCAGCTG
CAGCTAGCGAGTTAGTAATAGGAAGGGAACTGATGATCCATGCATGGACTGATGTGTGTTGCCCATCCCA
TCCCATTTCCCAACCCCAAACGAACCAAAACACACGTACTACGTGCAGGTGTGGCCGGCCTACGGCAACA
AGAAGTTCGAGACGCTGTCGTACCTGCCGCCGCTGTCGACCGGCGGGCGCATCCGCTGCATGCAGGCCAT
GGACAACTCCGTCCTGAACTCTGGTCGCACCACCATCTGCGACGCCTACAACGTCGCGGCGCATGATCCA
TTCAGCTTCCAGCACAAGAGCCTCGACACTGTTCAGAAGGAGTGGACGGAGTGGAAGAAGAACAACCACA
GCCTGTACCTGGACCCCATCGTCGGCACGGTGGCCAGCTTCCTTCTCAAGAAGGTCGGCTCTCTCGTCGG
GAAGCGCATCCTCTCGGAACTCCGCAACCTGATCTTTCCATCTGGCTCCACCAACCTCATGCAAGACATC
CTCAGGGAGACCGAGAAGTTTCTCAACCAGCGCCTCAACACTGATACCCTTGCTCGCGTCAACGCTGAGC
TGACGGGTCTGCAAGCAAACGTGGAGGAGTTCAACCGCCAAGTGGACAACTTCCTCAACCCCAACCGCAA
TGCGGTGCCTCTGTCCATCACTTCTTCCGTGAACACCATGCAACAACTGTTCCTCAACCGCTTGCCTCAG
TTCCAGATGCAAGGCTACCAGCTGCTCCTGCTGCCACTCTTTGCTCAGGCTGCCAACCTGCACCTCTCCT
TCATTCGTGACGTGATCCTCAACGCTGACGAGTGGGGCATCTCTGCAGCCACGCTGAGGACCTACCGCGA
CTACCTGAAGAACTACACCAGGGACTACTCCAACTATTGCATCAACACCTACCAGTCGGCCTTCAAGGGC

CTCAATACGAGGCTTCACGACATGCTGGAGTTCAGGACCTACATGTTCCTGAACGTGTTCGAGTACGTCA
GCATCTGGTCGCTCTTCAAGTACCAGAGCCTGCTGGTGTCCAGCGGCGCCAACCTCTACGCCAGCGGCTC
TGGTCCCCAACAAACTCAGAGCTTCACCAGCCAGGACTGGCCATTCCTGTATTCGTTGTTCCAAGTCAAC
TCCAACTACGTCCTCAACGGCTTCTCTGGTGCTCGCCTCTCCAACACCTTCCCCAACATTGTTGGCCTCC
CCGGCTCCACCACAACTCATGCTCTGCTTGCTGCCAGAGTGAACTACTCCGGCGGCATCTCGAGCGGCGA
CATTGGTGCATCGCCGTTCAACCAGAACTTCAACTGCTCCACCTTCCTGCCGCCGCTGCTCACCCCGTTC
GTGAGGTCCTGGCTCGACAGCGGCTCCGACCGCGAGGGCGTGGCCACCGTCACCAACTGGCAAACCGAGT
CCTTCGAGACCACCCTTGGCCTCCGGAGCGGCGCCTTCACGGCGCGTGGAAATTCTAACTACTTCCCCGA
CTACTTCATCAGGAACATCTCTGGTGTTCCTCTCGTCGTCCGCAACGAGGACCTCCGCCGTCCACTGCAC
TACAACGAGATCAGGAACATCGCCTCTCCGTCCGGGACGCCCGGAGGTGCAAGGGCGTACATGGTGAGCG
TCCATAACAGGAAGAACAACATCCACGCTGTGCATGAGAACGGCTCCATGATCCACCTGGCGCCCAATGA
TTACACCGGCTTCACCATCTCTCCAATCCACGCCACCCAAGTGAACAACCAGACACGCACCTTCATCTCC
GAGAAGTTCGGCAACCAGGGCGACTCCCTGAGGTTCGAGCAGAACAACACCACCGCCAGGTACACCCTGC
GCGGCAACGGCAACAGCTACAACCTGTACCTGCGCGTCAGCTCCATTGGCAACTCCACCATCAGGGTCAC
CATCAACGGGAGGGTGTACACAGCCACCAATGTGAACACGACGACCAACAATGATGGCGTCAACGACAAC
GGCGCCCGCTTCAGCGACATCAACATTGGCAACGTGGTGGCCAGCAGCAACTCCGACGTCCCGCTGGACA
TCAACGTGACCCTGAACTCTGGCACCCAGTTCGACCTCATGAACATCATGCTGGTGCCAACTAACATCTC
GCCGCTGTACTGATAGGAGCTCTGATCCCCATGGGAATTCCCGATCGTTCAAACATTTGGCAATAAAGTT
TCTTAAGATTGAATCCTGTTGCCGGTCTTGCGATGATTATCATATAATTTCTGTTGAATTACGTTAAGCA
TGTAATAATTAACATGTAATGCATGACGTTATTTATGAGATGGGTTTTTATGATTAGAGTCCCGCAATTA
TACATTTAATACGCGATAGAAAACAAAATATAGCGCGCAAACTAGGATAAATTATCGCGCGCGGTGTCAT
CTATGTTACTAGATCGGGGATATCCCCGGGGCGGCCGCGGGGAATTCGGTACCAAGCTTTGGCGCGCCAA
ATCGTGAAGTTTCTCATCTAAGCCCCCATTTGGACGTGAATGTAGACACGTCGAAATAAAGATTTCCGAA
TTAGAATAATTTGTTTATTGCTTTCGCCTATAAATACGACGGATCGTAATTTGTCGTTTTATCAAAATGT
ACTTTCATTTTATAATAACGCTGCGGACATCTACATTTTTGAATTGAAAAAAAATTGGTAATTACTCTTT
CTTTTTCTCCATATTGACCATCATACTCATTGCATCCCCGGAAATTATGTTTTTTTAAAAACCACGGTAT
TATAGATACCGTGTTATTTTTTGAGTATTGGAAATTTCATTTCAACCCAAAGTTTCTTCATGGCACATCT
AGCTTTTGCCTAATACCATGTAGGGCTACATCTTAAAAATCTATACTACTATATTAAAGCTGCAGGGGTA
GCCTGTCTCCACCTGGTTCTGCCTCGAGCCAATCTAAACCGTCCATCTATATCCATCAAATCAGCACCGT
CCGGTCCGTGCGCACCTCCTCTCCCGCTATTCAGTTGCATACTTGCAGCAGGTTCTCCCTCCTCACCATT
TCCTCTGCCTCCTCTCTCGCTCACTGGTCAGATTCATCCTGCCTCTCCCGCATGCGCTCCCTCCCCATGC
CCCGTCTCGCACTATCGCCACACCTCACCGCGGGGAGACGAAGACGGTGGACGCATCCTCACCTCCTCCG
CTAGTTGTCGCTCTTCCATCCTCTTCAACAACTTCTACATAGGGAGAGGCGGTTCGGCGTCCCGACGCCG
CCGCTTCTCCCCTCCCCATGGAGGACGAGAACATCGACCTCGGCGGCGGGGGCGATGCCTCCGCTCTGCA
TAGAGGAGGGTTGTAGTGGCAAGCAGCAATGCCAACACCGAGGCGGGCCAAGACTAGGCAACAATAGGAC
GGCACGCCCGGTTGTCAGCGAGGTGGCGGCATCGTGTGCCGCTACCGAACAACATCTCCGGCGCTGGAGT
CGGTGAGTTACTGCGCCACCCGGACGCCCTCAATGCACTGATATCTACCCGGTCTCCATCGCCGCCCTTC
CTCCCTTCCCTCTCCCTGTGCCTCCCTCTCTTGCCCTCTCCCTTCCAACTGCTCCCGCCCCAGCCCTAGC
CCAACCACCTCCCGCGCAGGGTCACCAA

>HV702026_MON88017

TACCCGATCAGAGCGCTAAGCAGCAGAATCGTGTGACAACGCTAGCAGCTCTCCTCCAACACATCATCGA
CAAGCACCTTTTTTGCCGGAGTATGACGGTGACGATATATTCAATTGTAAATGGCTTCATGTCCGGGAAA
TCTACATGGATCAGCAATGAGTATGATGGTCAATATGGAGAAAAAGAAAGAGTAATTACCAATTTTTTTT
CAATTCAAAAATGTAGATGTCCGCAGCGTTATTATAAAATGAAAGTACATTTTGATAAAACGACAAATTA
CGATCCGTCGTATTTATAGGCGAAAGCAATAAACAAATTATTCTAATTCGGAAATCTTTATTTCGACGTG
TCTACATTCACGTCCAAATGGGGGCTTAGATGAGAAACTTCACGATTTGGCGCGCCAAAGCTTACTCGAG
GTCATTCATATGCTTGAGAAGAGAGTCGGGATAGTCCAAAATAAAACAAAGGTAAGATTACCTGGTCAAA
AGTGAAAACATCAGTTAAAAGGTGGTATAAAGTAAAATATCGGTAATAAAAGGTGGCCCAAAGTGAAATT
TACTCTTTTCTACTATTATAAAAATTGAGGATGTTTTTGTCGGTACTTTGATACGTCATTTTTGTATGAA
TTGGTTTTTAAGTTTATTCGCTTTTGGAAATGCATATCTGTATTTGAGTCGGGTTTTAAGTTCGTTTGCT
TTTGTAAATACAGAGGGATTTGTATAAGAAATATCTTTAGAAAAACCCATATGCTAATTTGACATAATTT
TTGAGAAAAATATATATTCAGGCGAATTCTCACAATGAACAATAATAAGATTAAAATAGCTTTCCCCCGT
TGCAGCGCATGGGTATTTTTTCTAGTAAAAATAAAAGATAAACTTAGACTCAAAACATTTACAAAAACAA

CCCCTAAAGTTCCTAAAGCCCAAAGTGCTATCCACGATCCATAGCAAGCCCAGCCCAACCCAACCCAACC
CAACCCACCCCAGTCCAGCCAACTGGACAATAGTCTCCACACCCCCCCACTATCACCGTGAGTTGTCCGC
ACGCACCGCACGTCTCGCAGCCAAAAAAAAAAAGAAAGAAAAAAAAAGAAAAAGAAAAAACAGCAGGTGGG
TCCGGGTCGTGGGGGCCGGAAACGCGAGGAGGATCGCGAGCCAGCGACGAGGCCGGCCCTCCCTCCGCTT
CCAAAGAAACGCCCCCCATCGCCACTATATACATACCCCCCCCTCTCCTCCCATCCCCCCAACCCTACCA
CCACCACCACCACCACCTCCACCTCCTCCCCCCTCGCTGCCGGACGACGAGCTCCTCCCCCCTCCCCCTC
CGCCGCCGCCGCGCCGGTAACCACCCCGCCCCTCTCCTCTTTCTTTCTCCGTTTTTTTTTCCGTCTCGGT
CTCGATCTTTGGCCTTGGTAGTTTGGGTGGGCGAGAGGCGGCTTCGTGCGCGCCCAGATCGGTGCGCGGG
AGGGGCGGGATCTCGCGGCTGGGGCTCTCGCCGGCGTGGATCCGGCCCGGATCTCGCGGGGAATGGGGCT
CTCGGATGTAGATCTGCGATCCGCCGTTGTTGGGGGAGATGATGGGGGGTTTAAAATTTCCGCCGTGCTA
AACAAGATCAGGAAGAGGGGAAAAGGGCACTATGGTTTATATTTTTATATATTTCTGCTGCTTCGTCAGG
CTTAGATGTGCTAGATCTTTCTTTCTTCTTTTTGTGGGTAGAATTTGAATCCCTCAGCATTGTTCATCGG
TAGTTTTTCTTTTCATGATTTGTGACAAATGCAGCCTCGTGCGGAGCTTTTTTGTAGGTAGAAGTGATCA
ACCATGGCGCAAGTTAGCAGAATCTGCAATGGTGTGCAGAACCCATCTCTTATCTCCAATCTCTCGAAAT
CCAGTCAACGCAAATCTCCCTTATCGGTTTCTCTGAAGACGCAGCAGCATCCACGAGCTTATCCGATTTC
GTCGTCGTGGGGATTGAAGAAGAGTGGGATGACGTTAATTGGCTCTGAGCTTCGTCCTCTTAAGGTCATG
TCTTCTGTTTCCACGGCGTGCATGCTTCACGGTGCAAGCAGCCGGCCCGCAACCGCCCGCAAATCCTCTG
GCCTTTCCGGAACCGTCCGCATTCCCGGCGACAAGTCGATCTCCCACCGGTCCTTCATGTTCGGCGGTCT
CGCGAGCGGTGAAACGCGCATCACCGGCCTTCTGGAAGGCGAGGACGTCATCAATACGGGCAAGGCCATG
CAGGCGATGGGCGCCCGCATCCGTAAGGAAGGCGACACCTGGATCATCGATGGCGTCGGCAATGGCGGCC
TCCTGGCGCCTGAGGCGCCGCTCGATTTCGGCAATGCCGCCACGGGCTGCCGCCTGACGATGGGCCTCGT
CGGGGTCTACGATTTCGACAGCACCTTCATCGGCGACGCCTCGCTCACAAAGCGCCCGATGGGCCGCGTG
TTGAACCCGCTGCGCGAAATGGGCGTGCAGGTGAAATCGGAAGACGGTGACCGTCTTCCCGTTACCTTGC
GCGGGCCGAAGACGCCGACGCCGATCACCTACCGCGTGCCGATGGCCTCCGCACAGGTGAAGTCCGCCGT
GCTGCTCGCCGGCCTCAACACGCCCGGCATCACGACGGTCATCGAGCCGATCATGACGCGCGATCATACG
GAAAAGATGCTGCAGGGCTTTGGCGCCAACCTTACCGTCGAGACGGATGCGGACGGCGTGCGCACCATCC
GCCTGGAAGGCCGCGGCAAGCTCACCGGCCAAGTCATCGACGTGCCGGGCGACCCGTCCTCGACGGCCTT
CCCGCTGGTTGCGGCCCTGCTTGTTCCGGGCTCCGACGTCACCATCCTCAACGTGCTGATGAACCCCACC
CGCACCGGCCTCATCCTGACGCTGCAGGAAATGGGCGCCGACATCGAAGTCATCAACCCGCGCCTTGCCG
GCGGCGAAGACGTGGCGGACCTGCGCGTTCGCTCCTCCACGCTGAAGGGCGTCACGGTGCCGGAAGACCG
CGCGCCTTCGATGATCGACGAATATCCGATTCTCGCTGTCGCCGCCGCCTTCGCGGAAGGGGCGACCGTG
ATGAACGGTCTGGAAGAACTCCGCGTCAAGGAAAGCGACCGCCTCTCGGCCGTCGCCAATGGCCTCAAGC
TCAATGGCGTGGATTGCGATGAGGGCGAGACGTCGCTCGTCGTGCGTGGCCGCCCTGACGGCAAGGGGCT
CGGCAACGCCTCGGGCGCCGCCGTCGCCACCCATCTCGATCACCGCATCGCCATGAGCTTCCTCGTCATG
GGCCTCGTGTCGGAAAACCCTGTCACGGTGGACGATGCCACGATGATCGCCACGAGCTTCCCGGAGTTCA
TGGACCTGATGGCCGGGCTGGGCGCGAAGATCGAACTCTCCGATACGAAGGCTGCCTGATGAGCTCGAAT
TCCCGATCGTTCAAACATTTGGCAATAAAGTTTCTTAAGATTGAATCCTGTTGCCGGTCTTGCGATGATT
ATCATATAATTTCTGTTGAATTACGTTAAGCATGTAATAATTAACATGTAATGCATGACGTTATTTATGA
GATGGGTTTTTATGATTAGAGTCCCGCAATTATACATTTAATACGCGATAGAAAACAAAATATAGCGCGC
AAACTAGGATAAATTATCGCGCGCGGTGTCATCTATGTTACTAGATCGGGGATTTGCGGCCGCGTTAACA
AGCTTCTGCAGGTCCGATTGAGACTTTTCAACAAAGGGTAATATCCGGAAACCTCCTCGGATTCCATTGC
CCAGCTATCTGTCACTTTATTGTGAAGATAGTGGAAAAGGAAGGTGGCTCCTACAAATGCCATCATTGCG
ATAAAGGAAAGGCCATCGTTGAAGATGCCTCTGCCGACAGTGGTCCCAAAGATGGACCCCCACCCACGAG
GAGCATCGTGGAAAAAGAAGACGTTCCAACCACGTCTTCAAAGCAAGTGGATTGATGTGATGGTCCGATT
GAGACTTTTCAACAAAGGGTAATATCCGGAAACCTCCTCGGATTCCATTGCCCAGCTATCTGTCACTTTA
TTGTGAAGATAGTGGAAAAGGAAGGTGGCTCCTACAAATGCCATCATTGCGATAAAGGAAAGGCCATCGT
TGAAGATGCCTCTGCCGACAGTGGTCCCAAAGATGGACCCCCACCCACGAGGAGCATCGTGGAAAAAGAA
GACGTTCCAACCACGTCTTCAAAGCAAGTGGATTGATGTGATATCTCCACTGACGTAAGGGATGACGCAC
AATCCCACTATCCTTCGCAAGACCCTTCCTCTATATAAGGAAGTTCATTTCATTTGGAGAGGACACGCTG
ACAAGCTGACTCTAGCAGATCCTCTAGAACCATCTTCCACACACTCAAGCCACACTATTGGAGAACACAC
AGGGACAACACACCATAAGATCCAAGGGAGGCCTCCGCCGCCGCCGGTAACCACCCCGCCCCTCTCCTCT
TTCTTTCTCCGTTTTTTTTTCCGTCTCGGTCTCGATCTTTGGCCTTGGTAGTTTGGGTGGGCGAGAGGCG
GCTTCGTGCGCGCCCAGATCGGTGCGCGGGAGGGGCGGGATCTCGCGGCTGGGGCTCTCGCCGGCGTGGA
TCCGGCCCGGATCTCGCGGGGAATGGGGCTCTCGGATGTAGATCTGCGATCCGCCGTTGTTGGGGGAGAT
GATGGGGGGTTTAAAATTTCCGCCGTGCTAAACAAGATCAGGAAGAGGGGAAAAGGGCACTATGGTTTAT

ATTTTTATATATTTCTGCTGCTTCGTCAGGCTTAGATGTGCTAGATCTTTCTTTCTTCTTTTTGTGGGTA
GAATTTGAATCCCTCAGCATTGTTCATCGGTAGTTTTTCTTTTCATGATTTGTGACAAATGCAGCCTCGT
GCGGAGCTTTTTTGTAGGTAGAAGTGATCAACCATGGCCAACCCCAACAATCGCTCCGAGCACGACACGA
TCAAGGTCACCCCCAACTCCGAGCTCCAGACCAACCACAACCAGTACCCGCTGGCCGACAACCCCAACTC
CACCCTGGAAGAGCTGAACTACAAGGAGTTCCTGCGCATGACCGAGGACTCCTCCACGGAGGTCCTGGAC
AACTCCACCGTCAAGGACGCCGTCGGGACCGGCATCTCCGTCGTTGGGCAGATCCTGGGCGTCGTTGGCG
TCCCCTTCGCAGGTGCTCTCACCTCCTTCTACCAGTCCTTCCTGAACACCATCTGGCCCTCCGACGCCGA
CCCCTGGAAGGCCTTCATGGCCCAAGTCGAAGTCCTGATCGACAAGAAGATCGAGGAGTACGCCAAGTCC
AAGGCCCTGGCCGAGCTGCAAGGCCTGCAAAACAACTTCGAGGACTACGTCAACGCGCTGAACTCCTGGA
AGAAGACGCCTCTGTCCCTGCGCTCCAAGCGCTCCCAGGACCGCATCCGCGAGCTGTTCTCCCAGGCCGA
GTCCCACTTCCGCAACTCCATGCCGTCCTTCGCCGTCTCCAAGTTCGAGGTCCTGTTCCTGCCCACCTAC
GCCCAGGCTGCCAACACCCACCTCCTGTTGCTGAAGGACGCCCAGGTCTTCGGCGAGGAATGGGGCTACT
CCTCGGAGGACGTCGCCGAGTTCTACCGTCGCCAGCTGAAGCTGACCCAACAGTACACCGACCACTGCGT
CAACTGGTACAACGTCGGCCTGAACGGCCTGAGGGGCTCCACCTACGACGCATGGGTCAAGTTCAACCGC
TTCCGCAGGGAGATGACCCTGACCGTCCTGGACCTGATCGTCCTGTTCCCCTTCTACGACATCCGCCTGT
ACTCCAAGGGCGTCAAGACCGAGCTGACCCGCGACATCTTCACGGACCCCATCTTCCTGCTCACGACCCT
CCAGAAGTACGGTCCCACCTTCCTGTCCATCGAGAACTCCATCCGCAAGCCCCACCTGTTCGACTACCTC
CAGGGCATCGAGTTCCACACGCGCCTGAGGCCAGGCTACTTCGGCAAGGACTCCTTCAACTACTGGTCCG
GCAACTACGTCGAGACCAGGCCCTCCATCGGCTCCTCGAAGACGATCACCTCCCCTTTCTACGGCGACAA
GTCCACCGAGCCCGTCCAGAAGCTGTCCTTCGACGGCCAGAAGGTCTACCGCACCATCGCCAACACCGAC
GTCGCGGCTTGGCCGAACGGCAAGGTCTACCTGGGCGTCACGAAGGTCGACTTCTCCCAGTACGATGACC
AGAAGAACGAGACCTCCACCCAGACCTACGACTCCAAGCGCAACAATGGCCACGTCTCCGCCCAGGACTC
CATCGACCAGCTGCCGCCTGAGACCACTGACGAGCCCCTGGAGAAGGCCTACTCCCACCAGCTGAACTAC
GCGGAGTGCTTCCTGATGCAAGACCGCAGGGGCACCATCCCCTTCTTCACCTGGACCCACCGCTCCGTCG
ACTTCTTCAACACCATCGACGCCGAGAAGATCACCCAGCTGCCCGTGGTCAAGGCCTACGCCCTGTCCTC
GGGTGCCTCCATCATTGAGGGTCCAGGCTTCACCGGTGGCAACCTGCTGTTCCTGAAGGAGTCCTCGAAC
TCCATCGCCAAGTTCAAGGTCACCCTGAACTCCGCTGCCTTGCTGCAACGCTACCGCGTCCGCATCCGCT
ACGCCTCCACCACGAACCTGCGCCTGTTCGTCCAGAACTCCAACAATGACTTCCTGGTCATCTACATCAA
CAAGACCATGAACAAGGACGATGACCTGACCTACCAGACCTTCGACCTCGCCACCACGAACTCCAACATG
GGCTTCTCGGGCGACAAGAATGAACTGATCATTGGTGCTGAGTCCTTCGTCTCCAACGAGAAGATCTACA
TCGACAAGATCGAGTTCATCCCCGTCCAGCTGTGATAGGAACTCTGATTGAATTCTGCATGCGTTTGGAC
GTATGCTCATTCAGGTTGGAGCCAATTTGGTTGATGTGTGTGCGAGTTCTTGCGAGTCTGATGAGACATC
TCTGTATTGTGTTTCTTTCCCCAGTGTTTTCTGTACTTGTGTAATCGGCTAATCGCCAACAGATTCGGCG
ATGAATAAATGAGAAATAAATTGTTCTGATTTTGAGTGCAAAAAAAAAGGAATTAGATCTGTGTGTGTTT
TTTGGATCCCCGGGGCGGCCGCTCGAGCAGGACCTGCAGAAGCTAGCTTGATGGGGATCAGATTGTCGTT
TCCCGCCTTCAGTTTAAACAGAGTCGGGTTTGGATGGTCAACTCCGGCATACTGCCGAAAACAAACCAAT
CCGTCACCGTCAAGGCCCCGCACCGCTGGCCGCACGCAGGAAAAATAAGTTGCGACCGCGAGCGGGCGAA
TCAGAAAGGGCGTCCGGCCTTGGTCAGACACGACAGCGACGCGGAAAGGCTGCGCCCGCGGTGCCATCTA
CAAGGGTCCACGTCCATCCAAAAAGAGCGG

>EA135634_MON15985_3'_flank
CCAACTAACATCTCGCCGCTGTACTGATAGGAGCTCTGATCCCCATGGGAATTCCCGATCGTTCAAACAT
TTGGCAATAAAGTTTCTTAAGATTGAATCCTGTTGCCGGTCTTGCGATGATTATCATATAATTTCTGTTG
AATTACGTTAAGCATGTAATAATTAACATGTAATGCATGACGTTATTTATGAGATGGGTTTTTATGATTA
GAGTCCCGCAATTATACATTTAATACGCGATAGAAAACAAAATATAGCGCGCAAACTAGGATAAATTATC
GCGCGCGGTGTCATCTATGTTACTAGATCGGGGATATCCCCGGGGCGGCCGCTCTAGAACTAGTGGATCT
GCACTGAAATCCCATCCATTTAGCAACCTTAAAAGTAATAAACTTAAACTAAGTGTACTCCTTATCAAAA
CATAAACCAAGAGATGAGGATAACAGCGTTCGGCCAACCCCAATACCACCTATAATCAAAGCATGCGATG
TCACGAACGTTTTCCTGACGGCAATCGACACAAAGAAGAGATTAAAAAGGGAGACCACTATAGAAAGAAG
AAAGAGAATCGGCACCACTGTGAAACTACATTTGATAACCGTATTAGCAAGGCTCCACCATTGATAAAAA
CTCGACAACCAAGAGGAACTCACTTACCTTGATCAAAGTAGGGTTGTTGGTACTAAGGCTCAAAGCAAGG
AAAATGTCTAAATGAGTGAGTTGGGCTTTTTTAATTTAATTAAACTGAAAAATAAGAAAGGATGTTGCCA
ATTTTGTGTGGCAAGGACGGACAAGAGGTTTCGACCCAACCTAGAGAAGACATTAAAACTGCAGAACTGG
GCTCGCTTGTTCAGGATCGGGCTTTAAAGGAGAATGGTTTGATGGACCAGAATAGAGACAAACCAATACA
CTCAGATTTTGATCTTCCTTATTCTCCCTCAACTATTGATTTTGTTTTGGGTAATGATGGTGTAGCAGGG

GATCGGGTTTTAGGTGTTTTTCAAGGCATGACTGTTACTCATTTTAATCCGACTTGCGATGACCATAATG
AGATGGAGGCTGTACTGAAGGATGAAGTATTGGATCCAAATAGGCATTCGACATGTTTGATAAAACTCTT
AGTTACAAAATTCTTAGTAAACGAATCCATAAGGTTATCTTTAGATGTTATTTTCATCACATCTATAATT
CTTTCAGCTACTGCCTCTTGTATCAAGTGATACTTTCAGTCAGTATATTCGTCTTGCAGCACTGTTATCA
CAATACAGTGTTATATCTTTTTCTATATCAGGAATGACTTCAAGATCGGTTAAGAACTTTTAAAGCCATA
TTGTTTCTTTCGTAGCCTCAGAAGCAACCA

>EA135633_MON15985_5'_flank

CTTGTCATTGGTGCAGACAGGTTCGGGGAGTATGGCCTATGGGTGGTGGTAGAGAGAAAGTAACAGCGAA
ATCTTAGGGTGGTCCGAGAAATGAGATTGTTAAGTCCTGATTTAATGTGCTGAGTTCAGTTGATGGAGAG
GAAAATGGCAAAGATTTGAGGGGAGAGGATTTTGTGGATCCTAACATTAAACAGAATCGTTCTACTAAAA
GGGGTAAGGGTAGTGGTGGAAGAGATATTTTTAAAAAAGCTAAGGTAAAATCTGGGCAAAGTTTTAACGT
GGCCCAGGGGAATAAAAAAGGGACTATAGGGCCTAGTGGTAGTCAACAGGCCGGGCGGGAAAGTATTCAT
TTGAAATGGGTTTATTCGTATTAACAGCTACTCTAGGAGGAATGTTTTTATGCGGTGCTAACGATTTAAT
AACTATCTTTGTAGCTCCAGAATGTTTCAGTTTATGCTCCTACCTATTATCTGGATATACCAAGAAAGAT
GTACGGTCTAATGAGGCTACTACGAAATATTTACTCATGGGTGGGGCAAGCTCTTCTATTCTGGTTCATG
GTTTCTCTTGGCTATATGGTTCATCCGGGGGAGAGATCGAGCTTCAAGAAATAGTGAATGGTCTTATCAA
TACACAAATGTATAACTCCCCAGGAATTTCAATTGCGCTTATATTCATCACTGTAGGAATTGGGTTCAAG
CTTTCCCCAGCCCCTTCTCATCAATGGACTCCTGACGTATACGAAGGAGTTCTTTTAGGCTTTTGCATTT
TGTTGAAAATTCTTATTTTCACAGGACTTTGCTTATCATAATTATTGACAGCCAACTGGTGAAAATGGTG
CCACGAGAGCTCCCATCAGCATTGATTTGGCTAGGGATAGTTCTTTAAGTGCTAAGTCAATCATCTTGCA
AATTGACAATAAATCTCAACAGGTTTCAGCAAGTAAGGCCTCCTTCATTATTTTGGTTGCTTCTTTTTCA
TTAATGATGTTGCTTGTTTGCACCTGGAAAACATGCACCGTTCTTCATCATGCACCTTGGATATGGTCTT
GAGTGCTCTCTGACTGCCCGTCATCTTTCGAATTGGTGTCTGATTGAGTGCACCCGCCTAAGTTCAAGAT
GCCGGAGTTTGAATAGTATAACGGGACTAGTTGTCTTGAGGCTCATATTACTATGTTCTGTCGATATAAT
CATCCATGGGCTTCGCATCCACCATTGCAAGGCCCAACCACCTAGCACTGTGATGGGTCCAAACGCAAAG
GTGATCCCTTTAGGCCAAATGGATGGAGACGCTATAAGATTGGTCACCGCAAGAAAAGTGTGGATCGATA
GGCCCTGTAGTTGATGATGGGGATGATCACGATGACCCTGCCTATCCTCCAAGTTTCACCCCAACTAACA
TCCAAGTGCAACCAGAGGTGTACCCGCAGAGGGCACCTATTACCATTAGATCGACTGCAAGTGTAGGTAA
TGTTGGACAGGGTAGATCAGGGTGTTCACGGTGTGGTAGACTTTACTATGGTCCTTTTCGGGCAGGCGAA
AATGTTTGTTATAAATGCGGCACTCCAGATCATTTTGTACGAGAATGTCCAGAGGTGGCTAATCGAGAGA
ATCGAATCACTTACGAAAAAAGATTATTGTTACTAAGTTTTCATGTTTTATGCCATGCGTAGCCATGTTT
TTAATAAAGGGTTGAAGACTTGAATGACAAGTTAGCGTAAAGCATTTTTTTTCTTTCATGTTGGCACATT
TGATGCACTATGTCTTCGTCTATTTTTCAAACATACTGTTGGAAGAATTATGATTCACGTGTTGTTTAAG
ATCAAAAAGTGCATGCCTAACTAATACTTATCAGAAACAAATAATGCAATGAGTCATATCTCTATAAAGG
GTAATATCCGGAAACCTCCTCGGATTCCATTGCCCAGCTATCTGTCACTTTATTGTGAAGATAGTGGAAA
AGGAAGGTGGCTCCTACAAATGCCATCATTGCGATAAAGGAAAGGCCATCGTTGAAGATGCCTCTGCCGA
CAGTGGTCCCAAAGATGGACCCCCACCCACGAGGAGCATCGTGGAAAAAGAAGACGTTCCAACCACGTCT
TCAAAGCAAGTGGATTGATATGATATCTCCACTGACGTAAGGGATGACGCACAATCCCACTATCCTTCGC
AAGACCCTTCCTCTATATAAGGAAGTTCATTTCATTTGGAGAGGACACGCTGACAAGCTGACTCTAGCAG
ATCTCCATGGTCCGTCCTGTAGAAACC

Data S3. Annotations for reference sequences in CAF database.

```
##gff-version3
##sequence-region RIKILT20151130_MON810	1	4983
RIKILT20151130	manual	flank	1	803	.	+	.
	plantDNA_5'
RIKILT20151130	manual	promoter	804	1131	.	+	.	p35S
RIKILT20151130	manual	gene	1132	1937	.	+	.	hsp70
RIKILT20151130	manual	gene	1938	4394	.	+	.	cry1Ab
RIKILT20151130	manual	flank	4395	4983	.	+	.
	plantDNA_3'
##sequence-region JQ406878_MON810	1	855
JQ406878	manual	gene	1	66	.	+	.	cry1Ab
JQ406878	manual	flank	67	855	.	+	.	plantDNA_3'
##sequence-region JQ406879_MON810	1	1018
JQ406879	manual	flank	1	942	.	+	.	plantDNA_5'
JQ406879	manual	promoter	943	1018	.	+	.	p35S
##sequence-region AF434709_MON810	1	927
AF434709	manual	flank	1	803	.	+	.	plantDNA_5'
AF434709	manual	promoter	804	927	.	+	.	p35S
##sequence-region AY326434_MON810	1	4180
AY326434	manual	promoter	1	328	.	+	.	p35S
AY326434	manual	gene	323	1134	.	+	.	hsp70
AY326434	manual	gene	1135	3591	.	+	.	cry1Ab
AY326434	manual	flank	3581	4180	.	+	.	plantDNA_3'
##sequence-region DI362404_MON89034	1	12208
DI362404	manual	flank	1	2071	.	+	.	plantDNA_5'
DI362404	manual	promoter	2350	2651	.	+	.	p35S
DI362404	manual	gene	2678	2738	.	+	.	L-Ta.lhcbl
DI362404	manual	gene	2755	3234	.	+	.	Os.Act
DI362404	manual	gene	3244	6777	.	+	.	Cry1A
DI362404	manual	terminator	6809	7018	.	+	.	T-Hsp
DI362404	manual	promoter	7086	7649	.	+	.	pFMV
DI362404	manual	gene	7672	8475	.	+	.	Hsp70
DI362404	manual	gene	8492	8892	.	+	.	CTP2
DI362404	manual	gene	8893	10800	.	+	.	cry2Ab
DI362404	manual	terminator	10827	11377	.	+	.	tNOS
DI362404	manual	flank	11388	12208	.	+	.	plantDNA_3'
##sequence-region HV702026_MON88017	1	7450
HV702026	manual	flank	1	103	.	+	.	plantDNA_5'
HV702026	manual	plasmid_backbone	104	408	.	+	.
	plasmid
HV702026	manual	promoter	409	1814	.	+	.	P-ract
HV702026	manual	gene	1815	2048	.	+	.	CTP2
HV702026	manual	gene	2049	3420	.	+	.	cp4-epsps
HV702026	manual	terminator	3421	3964	.	+	.	tNOS
HV702026	manual	promoter	3695	4361	.	+	.	p35S
HV702026	manual	gene	4362	4440	.	+	.	wtCAB
HV702026	manual	intron	4441	4924	.	+	.
	rect_intron
HV702026	manual	gene	4925	6914	.	+	.	cry3Bb1
HV702026	manual	terminator	6915	7124	.	+	.	tahsp17
```

```
HV702026    manual      plasmid_backbone  7215  7229  .     +     .
      plasmid
HV702026    manual      flank 7230  7450  .     +     .     plantDNA_3'
##sequence-region EA135634_MON15985 1     1130
EA135634    manual      termnator   1     349   .     +     .     tNOS
EA135634    manual      flank 350   1130  .     +     .     plantDNA_3'
##sequence-region EA135633_MON15985 1     2267
EA135633    manual      flank1      1     361   .     +     .
      plantDNA_5'
EA135633    manual      chDNA 362   750   .     +     .     chDNA
EA135633    manual      flank2      751   1885  .     +     .     plantDNA
EA135633    manual      promoter    1886  2267  .     +     .     p35S
```

Data S4. Element database - redundant, containing element sequences from all input GMOs.

```
>lcl|JQ406878 MON810|cryIAb|1-66
ACAGCCACCACTTCTCCTTGGACATCGATGTGGGCTGCACCGACCTGAACGAGGACTTTCGGTAGC

>lcl|JQ406878 MON810|3'Maize|67-855
CTTCTTTCATTTCCGAATTTGCTTGCGAGCAGTCAGGTCCTTTTGATTCATCTGAGTTTGGCTTTACAATAGCTT
TTCCTTTTCCTTTGGCAGTACTAGTGCTTTCATCATGAGAATCCTTCTTAGATGTAAGACCACCTGCAGCAGATG
ACTTTGATCTTGTTGTTGGGCGCCGACCAGATTGAGCCATTGCAGCTGTTAATGATGCACCAGCCGTGGTGCCAG
GAACCCCAGATTCAGAATTATTACCAGATGGAATTATAGGCTTCGATGCAACCTCACTGCGTTGAACTCTAGGCC
AAAGGAATTCTTCAACAGATGCAAGACTAGCAAATGGGTCGATAAGCACAATATTTGATGAATAATCCCGAAGTG
ATTTTTCGCCTTGAGCTCGGGAAAGACGAAGCTTGAAGGGTTGAGCCAGAGCACTAAGACCTGAAGTCAGACGAG
ACCCTCCAATACCAATCCTACTAGACTGGCTGAGCACAACAGGGAAACGTTCCAGCGAAGACAATGCACTTTGCA
GTTTCTGAACCAACAGTGCCATGGGAGTTTCATCTCGTCCATGATCAATAGAAAGGGCAACAGATATGAAGGTTT
TGTATCGTCTAAGGGCCTGCTGACGAAGCTGAGGTAGGTTTCCTTCAGATACCTTTTCCTTCCCAAATGTCCCAC
AGGACAAATAGTCAAGCAACGCTATGACAACACCACTTCTAATGAATTCAAATGTGGAAACACCATTAACTTTGC
TAAGCTCAGAGAGCATTTCAGATATTATTTTGTCTAAT

>lcl|JQ406879 MON810|5'Maize|1-942
GAGACATCTTCGGAAATGCTGACAAAAGTGCTCTCAAAGCCGAAGCTTAAAAAATCTAAAAAGCCAAGCAAATGT
TGGTGCGCAAGAGCTGAAAATTGAGTAAGAAAGAGCAGATGGCAAGAAAGCGTGCCAAATGAGCTCGTGGTGCGC
TCTTATTTATACGCCTAGTGCGCTGAAAACTGGAAGGGCCCGCTTGTCAGTGACTGTTGCTATTCTAGCAAAGGA
AAGGTGTTTTTTCGGACCTTCGGCTTAGGGCCTTCGTCCATATCGCAATCTAAATTTATCATTCTAACAAATTAA
TATTACGAGGGGCTACTGTTGGTGGCCTTCGGCTTCTGAAGGTCCTCAAAAACATGATTTAACAAAGTTTCTGGA
GTATGATGCATGAACAGGTATCTTCGGACTTGAGTTAAAACCACAGTGTGAAGAAGCACAAAAGGAATACGAAGG
ATGTCGCGGAGCCGAAGCTGTGCGCAGAAGAGCTTCGAGATAATAGCAGAAAAGGAAACCGACTTAAAGATGAAA
AGGCTATTTAGACCTCGACGGATTACTATAGAGTTATTAGCAAATGTAGAGGGCATGGGTGTAATTTCATATGGG
CTGCGTCTCGTGCCTATAAATAGATGAACAGTGTTTCCGTACTGTTCGCGCTGACTTGGCATTTGCTTTTGCGCC
ACGCTTATACTTTTACCTTCTTTCAAGCCGAAGGTACATCTGTAATTTGATATCATTTCTATTCTTCCATGATAA
TAAAATAGAAATAAGTTGATTATAATATATAATTGTTTATGTTATCTCTTATACTTCATATGATTCCTTCTTCAT
TATTATATCTTGTGCTGATGAAGGTATGTCCTTCATAACCTTCGCCCGAAAATCATTATATCCCAAGGGAAATAA
TGCTTCGAAGGACGAAGGACTCTAACGTTTAACATCCTTTGC

>lcl|AF434709 MON810|5'Maize|1-803
TCGTGGTGCGCTCTTATTTATACGCCTAGTGCGCTGAAAACTGGAAGGGCCCGCTTGTCAGTGACTGTCGCTATT
CTAGCAAAGGAAAGGTGTTTTTTCGGACCTTCGGCTTAGGGCCTTCGTCCATATCGCAATCTAAATTTATCATTC
TAACAAATTAATATTACGAGGGGCTACTGTTGGTGGCCTTCGGCTTCTGAAGGTCCTCAAAAACATGATTTAACA
AAGTTTCTGGAGTATGATGCATGAACAGGTATCTTCGGACTTGAGTTAAAACYACAGTGTGAAGAAGCACAAAAG
GAATACGAAGGATGTCGCGGAGCCGAAGCTGTGCGCAGAAGAGCTTCGAGATAATAGCAGAAAAGGAAACCGACT
TAAAGATGAAAAGGCTATTTAGACCTCGACGGATTACTATAGAGTTATTAGCAAATGTAGAGGGCATGGGTGTAA
TTTCATATGGGCTGCGTCTCGTGCCTATAAATAGATGAACAGTGTTTCCGTACTGTTCGCGCTGACTTGGCATTT
GCTTTTGCGCCACGCTTATACTTTTACCTTCTTTCAAGCCGAAGGTACATCTGTAATTTGATATCATTTCTATTC
TTCCATGATAATAAAATAGAAATAAGTTGATTATAATATATAATTGTTTATGTTATCTCTTATACTTCATATGAT
TCCTTCTTCATTATTATATCTTGTGCTGATGAAGGTATGTCCTTCATAACCTTCGCCCGAAAATCATTATATCCC
AAGGGAAATAATGCTTCGAAGGACGAAGGACTCTAACGTTTAACATCCTTTGC

>lcl|AY326434 MON810|P35S|1-328
CATTGCCCAGCTATCTGTCACTTTATTGTGAAGATAGTGGAAAAGGAAGGTGGCTCCTACAAATGCCATCATTGC
GATAAAGGAAAGGCCATCGTTGAAGATGCCTCTGCCGACAGTGGTCCCAAAGATGGACCCCCACCCACGAGGAGC
ATCGTGGAAAAAGAAGACGTTCCAACCACGTCTTCAAAGCAAGTGGATTGATGTGATATCTCCACTGACGTAAGG
GATGACGCACAATCCCACTATCCTTCGCAAGACCCTTCCTCTATATAAGGAAGTTCATTTCATTTGGAGAGGACA
CGCTGACAAGCTGACTCTAGCAGATCTA
```

```
&gt;lcl|AY326434 MON810|hsp70|323-1134
GATCTACCGTCTTCGGTACGCGCTCACTCCGCCCTCTGCCTTTGTTACTGCCACGTTTCTCTGAATGCTCTCTTG
TGTGGTGATTGCTGAGAGTGGTTTAGCTGGATCTAGAATTACACTCTGAAATCGTGTTCTGCCTGTGCTGATTAC
TTGCCGTCCTTTGTAGCAGCAAAATATAGGGACATGGTAGTACGAAACGAAGATAGAACCTACACAGCAATACGA
GAAATGTGTAATTTGGTGCTTAGCGGTATTTATTTAAGCACATGTTGGTGTTATAGGGCACTTGGATTCAGAAGT
TTGCTGTTAATTTAGGCACAGGCTTCATACTACATGGGTCAATAGTATAGGGATTCATATTATAGGCGATACTAT
AATAATTTGTTCGTCTGCAGAGCTTATTATTTGCCAAAATTAGATATTCCTATTCTGTTTTTGTTTGTGTGCTGT
TAAATTGTTAACGCCTGAAGGAATAAATATAAATGACGAAATTTTGATGTTTATCTCTGCTCCTTTATTGTGACC
ATAAGTCAAGATCAGATGCACTTGTTTTAAATATTGTTGTCTGAAGAAATAAGTACTGACAGTATTTTGATGCAT
TGATCTGCTTGTTTGTTGTAACAAAATTTAAAAATAAAGAGTTTCCTTTTTGTTGCTCTCCTTACCTCCTGATGG
TATCTAGTATCTACCAACTGACACTATATTGCTTCTCTTTACATACGTATCTTGCTCGATGCCTTCTCCCTAGTG
TTGACCAGTGTTACTCACATAGTCTTTGCTCATTTCATTGTAATGCAGATACCAAGCGGCCA

&gt;lcl|AY326434 MON810|cry1Ab|1135-3591
TGGACAACAACCCAAACATCAACGAGTGCATCCCGTACAACTGCCTCAGCAACCCTGAGGTCGAGGTGCTCGGCG
GTGAGCGCATCGAGACCGGTTACACCCCCATCGACATCTCCCTCTCCCTCACGCAGTTCCTGCTCAGCGAGTTCG
TGCCAGGCGCTGGCTTCGTCCTGGGCCTCGTGGACATCATCTGGGGCATCTTTGGCCCCTCCCAGTGGGACGCCT
TCCTGGTGCAAATCGAGCAGCTCATCAACCAGAGGATCGAGGAGTTCGCCAGGAACCAGGCCATCAGCCGCCTGG
AGGGCCTCAGCAACCTCTACCAAATCTACGCTGAGAGCTTCCGCGAGTGGGAGGCCGACCCCACTAACCCAGCTC
TCCGCGAGGAGATGCGCATCCAGTTCAACGACATGAACAGCGCCCTGACCACCGCCATCCCACTCTTCGCCGTCC
AGAACTACCAAGTCCCGCTCCTGTCCGTGTACGTCCAGGCCGCCAACCTGCACCTCAGCGTGCTGAGGGACGTCA
GCGTGTTTGGCCAGAGGTGGGGCTTCGACGCCGCCACCATCAACAGCCGCTACAACGACCTCACCAGGCTGATCG
GCAACTACACCGACCACGCTGTCCGCTGGTACAACACTGGCCTGGAGCGCGTCTGGGGCCCTGATTCTAGAGACT
GGATTCGCTACAACCAGTTCAGGCGCGAGCTGACCCTCACCGTCCTGGACATTGTGTCCCTCTTCCCGAACTACG
ACTCCCGCACCTACCCGATCCGCACCGTGTCCCAACTGACCCGCGAAATCTACACCAACCCCGTCCTGGAGAACT
TCGACGGTAGCTTCAGGGGCAGCGCCCAGGGCATCGAGGGCTCCATCAGGAGCCCACACCTGATGGACATCCTCA
ACAGCATCACTATCTACACCGATGCCCACCGCGGCGAGTACTACTGGTCCGGCCACCAGATCATGGCCTCCCCGG
TCGGCTTCAGCGGCCCCGAGTTTACCTTTCCTCTCTACGGCACGATGGGCAACGCCGCTCCACAACAACGCATCG
TCGCTCAGCTGGGCCAGGGCGTCTACCGCACCCTGAGCTCCACCCTGTACCGCAGGCCCTTCAACATCGGTATCA
ACAACCAGCAGCTGTCCGTCCTGGATGGCACTGAGTTCGCCTACGGCACCTCCTCCAACCTGCCCTCCGCTGTCT
ACCGCAAGAGCGGCACGGTGGATTCCCTGGACGAGATCCCACCACAGAACAACAATGTGCCCCCCAGGCAGGGTT
TTTCCCACAGGCTCAGCCACGTGTCCATGTTCCGCTCCGGCTTCAGCAACTCGTCCGTGAGCATCATCAGAGCTC
CTATGTTCTCCTGGATTCATCGCAGCGCGGAGTTCAACAATATCATTCCGTCCTCCCAAATCACCCAAATCCCCC
TCACCAAGTCCACCAACCTGGGCAGCGGCACCTCCGTGGTGAAGGGCCCAGGCTTCACGGGCGGCGACATCCTGC
GCAGGACCTCCCCGGGCCAGATCAGCACCCTCCGCGTCAACATCACCGCTCCCCTGTCCCAGAGGTACCGCGTCA
GGATTCGCTACGCTAGCACCACCAACCTGCAATTCCACACCTCCATCGACGGCAGGCCGATCAATCAGGGTAACT
TCTCCGCCACCATGTCCAGCGGCAGCAACCTCCAATCCGGCAGCTTCCGCACCGTGGGTTTCACCACCCCCTTCA
ACTTCTCCAACGGCTCCAGCGTTTTCACCCTGAGCGCCCACGTGTTCAATTCCGGCAATGAGGTGTACATTGACC
GCATTGAGTTCGTGCCAGCCGAGGTCACCTTCGAAGCCGAGTACGACCTGGAGAGAGCCCAGAAGGCTGTCAATG
AGCTCTTCACGTCCAGCAATCAGATCGGCCTGAAGACCGACGTCACTGACTACCACATCGACCAAGTCTCCAACC
TCGTGGAGTGCCTCTCCGATGAGTTCTGCCTCGACGAGAAGAAGGAGCTGTCCGAGAAGGTGAAGCATGCCAAGC
GTCTCAGCGACGAGAGGAATCTCCTCCAGGACCCCAATTTCCGCGGCATCAACAGGCAGCTCGACCGCGGCTGGC
GCGGCAGCACCGACATCACGATCCAGGGCGGCGACGATGTGTTCAAGGAGAACTACGTGACTCTCCTGGGCACTT
TCGACGAGTGCTACCCTACCTACTTGTACCAGAAGATCGATGAGTCCAAGCTCAAGGCTTACACTCGCTACCAGC
TCCGCGGCTACATCGAAGACAGCCAAGACCTCGAGATTTACCTGATCCGCTACAACGCCAAGCACGAGACCGTCA
ACGTGCCCGGTACTGGTTCCCTCTGGCCGCTGAGCGCCCCCAGCCCGATCGGCAAGTGTGCCCACCACAGCCACC
ACTTCTCCTTGGACATCGATGTGGGCTGCACCGACCTGAACGAGGACTTTCGGTAGC

&gt;lcl|AY326434 MON810|3'Maize|3581-4180
CTTTCGGTAGCCTTCTTTCATTTCCGAATTTGCTTGCGAGCAGTCAGGTCCTTTTGATTCATCTGAGTTTGGCTT
TACAATAGCTTTTCCTTTTCCTTTGGCAGTACTAGTGCTTTCATCATGAGAATCCTTCTTAGATGTAAGACCACC
TGCAGCAGATGACTTTGATCTTGTTGTTGGGCGCCGACCAGATTGAGCCATTGCAGCTGTTAATGATGCACCAGC
CGTGGTGCCAGGAACCCCAGATTCAGAATTATTACCAGATGGAATTATAGGCTTCGATGCAACCTCACTGCGTTG
```

AACTCTAGGCCAAAGGAATTCTTCAACAGATGCAAGACTAGCAAATGGGTCGATAAGCACAATATTTGATGAATA
ATCCCGAAGTGATTTTTCGCCATGAGCTCGGGAAAGACGAAGCTTGAAGGGTTGAGCCAGAGCACTAAGACCTGA
AGTCAGACGAGACCCTCCAATACCAATCCTACTAGACTGGCTGAGCACAACAGGGAAACGTTCCAGCGAAGACAA
TGCACTTTGCAGTTTCTGAACCAACAGTGCCATGGGAGTTTCATCTCTCTCGACCAATCGAATTTCCGCGGGCC

>lcl|DI362404 MON89034|5'Maize|1-2071
ATATTTAAAAATGGAAGTAATACTATATTAAAATGATTCATGTGGAACTCCTGCGCTTCTTTTTGAAGTTTCAAA
AGGGAGCTTTCAGGGTCGCTTAGAGTTTGTTTGGTTGGAAATACAAGCGAAAAGAGAGCTAATGAGGGGGACATC
CATATTTTCTATGGTGTTTGAATAAGAGTCACGCGGGAATAAGATGAACACCGAAACAATTTTTTTGTAGCTACG
TGGTTCCAAAAAATCGAGTAGACGGTGTCGCTTCCACCTCATACTACTTCAACCTCAAACCACACATCCTTACGC
GCCCGCCGGTGTCTCCGTCGTCTCAAGTTCTCAACATACCTACACATGTAACCACTACCGGTCTTTGTCATGTTT
CGAAAGAAGATTACAGGTCCTCTAGAAGAGAGGACGCGGGGTGGCGAGAAAGCTGGGGAAGAAAAAGGCTAGTAC
ATATGATTGGTCTGTGAACCTGTGAGGTGGGTAGGTAGGTAGGTGGAGATTTTTGTTAACTGGTGTTGTTGACGG
ACTCGAACGGGGCCGGGCGTGTGGTGTGGCTAGCTGTGGTGGTTTGCTCGCCAGCCAGCCAGCCACACATCAGCG
AGCATGCAGAGCTTAAGCATGTATGTACGGATCGGCTTGCTTAGCGGTTAAGGGTGTGTTTGGTTTGGCTTTTGG
CTTTGGCTTTTGCCCCCTAAAAGCCAAAAGCCAACCAAGGGTGTATTTGGTTTGACTTTTGGCTTTTGGCTTTTG
TCCCCTAAAAGCCAAAAGCCAAACAAAGGGTTAGATCTAGGAAGCAGCTTTTTCTAAAAGCTGGCTTTCTCACAG
TGCAAATCTGAAAGCACCCCTGAACCTGCTTTTAGTGGCTTTTCGAATGGAACTGTGAAAACATATATCGAAGAA
CTTTTAACGACTTTTAGTGGTTTCCACCAAACAGTTTAGCTTTTTAACGGCTTACAGCCTACAACAGCTTTTTCC
ACAGCTCACAGCCCACAGCAACTTTTTTCACAGCCACAGCCCAACCAAACAGACCCCAAAGGGCTGAATCCAGGA
AGCAGCTTTTTCTAAAAGCCGACTTTCTCGTAGTGTAAAACTGAAAACACCCCTGGACCTGCTTTTAGTGGCTTT
TGGATGGAACTGTGAAAACATATATCGAAGAACTTTTAACGACTTTTAGTGGTTTCCACCAAACGATTTCTAGCT
TTTTAACAGCACACAGCCTACAACAGCTTTTTTCACAGCTCACAGCCCACAACAACTTTTTCTACAGCCACAACC
CAACCAAACGGACCCTAAGGCGGCCGAGCGAGCGCAAAGCGTCGTCAGCTTTGATTGCCATGCCATCTCCTGCTC
CACTTGTCTCTCTGGCCGTCGTCAGCCACCATCCAACAAGGCCGGTGCTACTGGCGGCTCCTAGGTAGACGACGA
CGACGACGACACCTCCACCGTTCGCCGCCGTCCACTCACCAATCAACACGGAACGCCCAAAACACACACACACGC
ACGCTGGGGAGGGAAAAAAAGGCAGAGACATATGCGTGCGTCGCTGCATTATTGTACGCGATCGAATGGCATCTC
TCTCACTCTCTCTCCTCCCTTTATTAATCTGGTACTGGCTAGCTGGTCCGGCGACACCGACGTGTCAGCTCCGTC
GCGCGCGTCCGTCCGTCCGCTGGAGCGGACACGGACCGCGGCGTCTGTCGATCGCCGGCTCGCCGCAGCGCAGCT
ACCTAGCACGCTCACGCATGCTACACTGCCTACACGCACACGGCCGGCCCAAAAGCGTTCCCTGCCGCCTGCCGG
CCGGCTTTTTTATTATTATTGGAACATGAGGCTATTTCTCCTCCCACACGGGCTACGACGTGAGCACGAGTACTG
GGATCCCCGGATCCGCCCTCTCTGTCCCTGCTGCTACTCCAGCCACTGAAATGTTGTCAGATGAAACAGCAGAGC
CGATCTCCGCACGGAAACCCATGCACGGCCATTCAAATTCAGGTGCCCACGTACGTCAGGGTGCTGCTGCTACTA
CTATCAAGCCAATAAAAGGATGGTAATGAGTATGATGGATCAGCAA

>lcl|DI362404 MON89034|P35S|2350-2651
CGATCCTACCTGTCACTTCATCAAAAGGACAGTAGAAAAGGAAGGTGGCTCCTACAAATGCCATCATTGCGATAA
AGGAAAGGCCATCGTTGAAGATGCCTCTGCCGACAGTGGTCCCAAAGATGGACCCCCACCCACGAGGAGCATCGT
GGAAAAAGAAGACGTTCCAACCACGTCTTCAAAGCAAGTGGATTGATGTGATATCTCCACTGACGTAAGGGATGA
CGCACAATCCCACTATCCTTCGCAAGACCCTTCCTCTATATAAGGAAGTTCATTTCATTTGGAGAGGACACGCTG
AC

>lcl|DI362404 MON89034|LTa.Ihcbl|2678-2738
ACCATCTTCCACACACTCAAGCCACACTATTGGAGAACACACAGGGACAACACACCATAAG

>lcl|DI362404 MON89034|Os.Act|2755-3234
CGCCGCCGCCGGTAACCACCCCGCCCCTCTCCTCTTTCTTTCTCCGTTTTTTTTTCCGTCTCGGTCTCGATCTTT
GGCCTTGGTAGTTTGGGTGGGCGAGAGGCGGCTTCGTGCGCGCCCAGATCGGTGCGCGGGAGGGGCGGGATCTCG
CGGCTGGGGCTCTCGCCGGCGTGGATCCGGCCCGGATCTCGCGGGGAATGGGGCTCTCGGATGTAGATCTGCGAT
CCGCCGTTGTTGGGGGAGATGATGGGGGGTTTAAAATTTCCGCCGTGCTAAACAAGATCAGGAAGAGGGGAAAAG
GGCACTATGGTTTATATTTTTATATATTTCTGCTGCTTCGTCAGGCTTAGATGTGCTAGATCTTTCTTTCTTCTT
TTTGTGGGTAGAATTTGAATCCCTCAGCATTGTTCATCGGTAGTTTTTCTTTTCATGATTTGTGACAAATGCAGC
CTCGTGCGGAGCTTTTTTGTAGGTAGAAGT

>lcl|DI362404 MON89034|Cry1A.105|3244-6777
TGGACAACAACCCAAACATCAACGAGTGCATCCCGTACAACTGCCTCAGCAACCCTGAGGTCGAGGTGCTCGGCG
GTGAGCGCATCGAGACCGGTTACACCCCCATCGACATCTCCCTCTCCCTCACGCAGTTCCTGCTCAGCGAGTTCG
TGCCAGGCGCTGGCTTCGTCCTGGGCCTCGTGGACATCATCTGGGGCATCTTTGGCCCCTCCCAGTGGGACGCCT
TCCTGGTGCAAATCGAGCAGCTCATCAACCAGAGGATCGAGGAGTTCGCCAGGAACCAGGCCATCAGCCGCCTGG
AGGGCCTCAGCAACCTCTACCAAATCTACGCTGAGAGCTTCCGCGAGTGGGAGGCCGACCCCACTAACCCAGCTC
TCCGCGAGGAGATGCGCATCCAGTTCAACGACATGAACAGCGCCCTGACCACCGCCATCCCACTCTTCGCCGTCC
AGAACTACCAAGTCCCGCTCCTGTCCGTGTACGTCCAGGCCGCCAACCTGCACCTCAGCGTGCTGAGGGACGTCA
GCGTGTTTGGCCAGAGGTGGGGCTTCGACGCCGCCACCATCAACAGCCGCTACAACGACCTCACCAGGCTGATCG
GCAACTACACCGACCACGCTGTCCGCTGGTACAACACTGGCCGTCCTGGACATTGTGTCCCTCTTCCCGAACTAC
GACTCCCGCACCTACCCGATCCGCACCGTGTCCCAACTGACCCGCGAAATCTACACCAACCCCGTCCTGGAGAAC
TTCGACGGTAGCTTCAGGGGCAGCGCCCAGGGCATCGAGGGCTCCATCAGGAGCCCACACCTGATGGACATCCTC
AACAGCATCACTATCTACACCGATGCCCACCGCGGCGAGTACTACTGGTCCGGCCACCAGATCATGGCCTCCCCG
GTCGGCTTCAGCGGCCCCGAGTTTACCTTTCCTCTCTACGGCACGATGGGCAACGCCGCTCCACAACAACGCATC
GTCGCTCAGCTGGGCCAGGGCGTCTACCGCACCCTGAGCTCCACCCTGTACCGCAGGCCCTTCAACATCGGTATC
AACAACCAGCAGCTGTCCGTCCTGGATGGCACTGAGTTCGCCTACGGCACCTCCTCCAACCTGCCCTCCGCTGTC
TACCGCAAGAGCGGCACGGTGGATTCCCTGGACGAGATCCCACCACAGAACAACAATGTGCCCCCCAGGCAGGGT
TTTTCCCACAGGCTCAGCCACGTGTCCATGTTCCGCTCCGGCTTCAGCAACTCGTCCGTGAGCATCATCAGAGCT
CCTATGTTCTCTTGGATACACCGTAGTGCTGAGTTCAACAACATCATTGCATCCGACAGCATTACTCAAATACCC
TTGGTGAAAGCACATACACTTCAGTCAGGTACTACTGTTGTCAGAGGTCCAGGGTTTACAGGAGGAGACATTCTT
CGTCGCACAAGTGGAGGACCCTTTGCTTACACTATTGTTAACATCAATGGCCAATTGCCCCAAAGGTATCGTGCA
AGAATCCGCTATGCCTCTACTACAAATCTCAGGATCTACGTGACTGTTGCAGGTGAAAGGATCTTTGCTGGTCAG
TTCAACAAGACTATGGATACCGGTGACCCTTTGACATTCCAATCTTTTAGCTACGCAACTATCAACACAGCTTTT
ACATTCCCAATGAGCCAGAGTAGCTTCACAGTAGGTGCTGACACTTTCAGCTCAGGGAATGAAGTTTACATCGAC
AGGTTTGAATTGATTCCAGTTACTGCAACCCTCGAGGCTGAGTACAACCTTGAGAGAGCCCAGAAGGCTGTGAAC
GCCCTCTTTACCTCCACCAATCAGCTTGGCTTGAAAACTAACGTTACTGACTATCACATTGACCAAGTGTCCAAC
TTGGTCACCTACCTTAGCGATGAGTTCTGCCTCGACGAGAAGCGTGAACTCTCCGAGAAAGTTAAACACGCCAAG
CGTCTCAGCGACGAGAGGAATCTCTTGCAAGACTCCAACTTCAAAGACATCAACAGGCAGCCAGAACGTGGTTGG
GGTGGAAGCACCGGGATCACCATCCAAGGAGGCGACGATGTGTTCAAGGAGAACTACGTCACCCTCTCCGGAACT
TTCGACGAGTGCTACCCTACCTACTTGTACCAGAAGATCGATGAGTCCAAACTCAAAGCCTTCACCAGGTATCAA
CTTAGAGGCTACATCGAAGACAGCCAAGACCTTGAAATCTACTCGATCAGGTACAATGCCAAGCACGAGACCGTG
AATGTCCCAGGTACTGGTTCCCTCTGGCCACTTTCTGCCCAATCTCCCATTGGGAAGTGTGGAGAGCCTAACAGA
TGCGCTCCACACCTTGAGTGGAATCCTGACTTGGACTGCTCCTGCAGGGATGGCGAGAAGTGTGCCCACCATTCT
CATCACTTCTCCTTGGACATCGATGTGGGATGTACTGACCTGAATGAGGACCTCGGAGTCTGGGTCATCTTCAAG
ATCAAGACCCAAGACGGACACGCAAGACTTGGCAACCTTGAGTTTCTCGAAGAGAAACCATTGGTCGGTGAAGCT
CTCGCTCGTGTGAAGAGAGCAGAGAAGAAGTGGAGGGACAAACGTGAGAAACTCGAATGGGAAACTAACATCGTT
TACAAGGAGGCCAAAGAGTCCGTGGATGCTTTGTTCGTGAACTCCCAATATGATCAGTTGCAAGCCGACACCAAC
ATCGCCATGATCCACGCCGCAGACAAACGTGTGCACAGCATTCGTGAGGCTTACTTGCCTGAGTTGTCCGTGATC
CCTGGTGTGAACGCTGCCATCTTCGAGGAACTTGAGGGACGTATCTTTACCGCATTCTCCTTGTACGATGCCAGA
AACGTCATCAAGAACGGTGACTTCAACAATGGCCTCAGCTGCTGGAATGTGAAAGGTCATGTGGACGTGGAGGAA
CAGAACAATCAGCGTTCCGTCCTGGTTGTGCCTGAGTGGGAAGCTGAAGTGTCCCAAGAGGTTAGAGTCTGTCCA
GGTAGAGGCTACATTCTCCGTGTGACCGCTTACAAGGAGGGATACGGTGAGGGTTGCGTGACCATCCACGAGATC
GAGAACAACACCGACGAGCTTAAGTTCTCCAACTGCGTCGAGGAAGAAATCTATCCCAACAACACCGTTACTTGC
AACGACTACACTGTGAATCAGGAAGAGTACGGAGGTGCCTACACTAGCCGTAACAGAGGTTACAACGAAGCTCCT
TCCGTTCCTGCTGACTATGCCTCCGTGTACGAGGAGAAATCCTACACAGATGGCAGACGTGAGAACCCTTGCGAG
TTCAACAGAGGTTACAGGGACTACACACCACTTCCAGTTGGCTATGTTACCAAGGAGCTTGAGTACTTTCCTGAG
ACCGACAAAGTGTGGATCGAGATCGGTGAAACCGAGGGAACCTTCATCGTGGACAGCGTGGAGCTTCTCTTGATG
GAGGAATAATGAGATCTATCGATTCTAGAAGGCCTGAATTCTGCATGCGTTTGGACGTATGCTCATTCAGGTTGG
AGCCAATTT

>lcl|DI362404 MON89034|T-Hsp|6809-7018

```
GATGAGACATCTCTGTATTGTGTTTCTTTCCCCAGTGTTTTCTGTACTTGTGTAATCGGCTAATCGCCAACAGAT
TCGGCGATGAATAAATGAGAAATAAATTGTTCTGATTTTGAGTGCAAAAAAAAAGGAATTAGATCTGTGTGTGTT
TTTTGGATCCCCGGGGCGGCCGCGTTAACAAGCTTGAGCTCAGGATTTAGCAGCATTCCA

>lcl|DI362404 MON89034|P-FMV|7086-7649
AGGAACTCCCATCCTCAAAGGTTTGTAAGGAAGAATTCTCAGTCCAAAGCCTCAACAAGGTCAGGGTACAGAGTC
TCCAAACCATTAGCCAAAAGCTACAGGAGATCAATGAAGAATCTTCAATCAAAGTAAACTACTGTTCCAGCACAT
GCATCATGGTCAGTAAGTTTCAGAAAAAGACATCCACCGAAGACTTAAAGTTAGTGGGCATCTTTGAAAGTAATC
TTGTCAACATCGAGCAGCTGGCTTGTGGGGACCAGACAAAAAAGGAATGGTGCAGAATTGTTAGGCGCACCTACC
AAAAGCATCTTTGCCTTTATTGCAAAGATAAAGCAGATTCCTCTAGTACAAGTGGGGAACAAAATAACGTGGAAA
AGAGCTGTCCTGACAGCCCACTCACTAATGCGTATGACGAACGCAGTGACGACCACAAAAGAATTCCCTCTATAT
AAGAAGGCATTCATTCCCATTTGAAGGATCATCAGATACTCAACCAATCCTTCTAGGATCTACCGTCTTCGGTAC
GCGCTCACTCCGCCCTCTGCCTTTGTTACTGCCACGTTT

>lcl|DI362404 MON89034|Hsp70|7672-8475
TGATTGCTGAGAGTGGTTTAGCTGGATCTAGAATTACACTCTGAAATCGTGTTCTGCCTGTGCTGATTACTTGCC
GTCCTTTGTAGCAGCAAAATATAGGGACATGGTAGTACGAAACGAAGATAGAACCTACACAGCAATACGAGAAAT
GTGTAATTTGGTGCTTAGCGGTATTTATTTAAGCACATGTTGGTGTTATAGGGCACTTGGATTCAGAAGTTTGCT
GTTAATTTAGGCACAGGCTTCATACTACATGGGTCAATAGTATAGGGATTCATATTATAGGCGATACTATAATAA
TTTGTTCGTCTGCAGAGCTTATTATTTGCCAAAATTAGATATTCCTATTCTGTTTTTGTTTGTGTGCTGTTAAAT
TGTTAACGCCTGAAGGAATAAATATAAATGACGAAATTTTGATGTTTATCTCTGCTCCTTTATTGTGACCATAAG
TCAAGATCAGATGCACTTGTTTTAAATATTGTTGTCTGAAGAAATAAGTACTGACAGTATTTTGATGCATTGATC
TGCTTGTTTGTTGTAACAAAATTTAAAAATAAAGAGTTTCCTTTTTGTTGCTCTCCTTACCTCCTGATGGTATCT
AGTATCTACCAACTGACACTATATTGCTTCTCTTTACATACGTATCTTGCTCGATGCCTTCTCCCTAGTGTTGAC
CAGTGTTACTCACATAGTCTTTGCTCATTTCATTGTAATGCAGATACCAAGCGGCCTCTAGAGGATCAGCATGGC
GCCCACCGTGATGATGGCCTCGTCGGCCACCGCCGTCGCTCCGTTCCAGGGGCT

>lcl|DI362404 MON89034|Zm_CTP2|8492-8892
CTCCCCGTCGCCCGCCGCTCCTCCAGAAGCCTCGGCAACGTCAGCAACGGCGGAAGGATCCGGTGCATGCAGGTA
ACAAATGCATCCTAGCTAGTAGTTCTTTGCATTGCAGCAGCTGCAGCTAGCGAGTTAGTAATAGGAAGGGAACTG
ATGATCCATGCATGGACTGATGTGTGTTGCCCATCCCATCCCATTTCCCAACCCCAAACGAACCAAAACACACGT
ACTACGTGCAGGTGTGGCCGGCCTACGGCAACAAGAAGTTCGAGACGCTGTCGTACCTGCCGCCGCTGTCGACCG
GCGGGCGCATCCGCTGCATGCAGGCCATGGACAACTCCGTCCTGAACTCTGGTCGCACCACCATCTGCGACGCCT
ACAACGTCGCGGCGCATGATCCATTC

>lcl|DI362404 MON89034|Cry2Ab|8893-10745
AGCTTCCAGCACAAGAGCCTCGACACTGTTCAGAAGGAGTGGACGGAGTGGAAGAAGAACAACCACAGCCTGTAC
CTGGACCCCATCGTCGGCACGGTGGCCAGCTTCCTTCTCAAGAAGGTCGGCTCTCTCGTCGGGAAGCGCATCCTC
TCGGAACTCCGCAACCTGATCTTTCCATCTGGCTCCACCAACCTCATGCAAGACATCCTCAGGGAGACCGAGAAG
TTTCTCAACCAGCGCCTCAACACTGATACCCTTGCTCGCGTCAACGCTGAGCTGACGGGTCTGCAAGCAAACGTG
GAGGAGTTCAACCGCCAAGTGGACAACTTCCTCAACCCCAACCGCAATGCGGTGCCTCTGTCCATCACTTCTTCC
GTGAACACCATGCAACAACTGTTCCTCAACCGCTTGCCTCAGTTCCAGATGCAAGGCTACCAGCTGCTCCTGCTG
CCACTCTTTGCTCAGGCTGCCAACCTGCACCTCTCCTTCATTCGTGACGTGATCCTCAACGCTGACGAGTGGGGC
ATCTCTGCAGCCACGCTGAGGACCTACCGCGACTACCTGAAGAACTACACCAGGGACTACTCCAACTATTGCATC
AACACCTACCAGTCGGCCTTCAAGGGCCTCAATACGAGGCTTCACGACATGCTGGAGTTCAGGACCTACATGTTC
CTGAACGTGTTCGAGTACGTCAGCATCTGGTCGCTCTTCAAGTACCAGAGCCTGCTGGTGTCCAGCGGCGCCAAC
CTCTACGCCAGCGGCTCTGGTCCCCAACAAACTCAGAGCTTCACCAGCCAGGACTGGCCATTCCTGTATTCGTTG
TTCCAAGTCAACTCCAACTACGTCCTCAACGGCTTCTCTGGTGCTCGCCTCTCCAACACCTTCCCCAACATTGTT
GGCCTCCCCGGCTCCACCACAACTCATGCTCTGCTTGCTGCCAGAGTGAACTACTCCGGCGGCATCTCGAGCGGC
GACATTGGTGCATCGCCGTTCAACCAGAACTTCAACTGCTCCACCTTCCTGCCGCCGCTGCTCACCCCGTTCGTG
AGGTCCTGGCTCGACAGCGGCTCCGACCGCGAGGGCGTGGCCACCGTCACCAACTGGCAAACCGAGTCCTTCGAG
ACCACCCTTGGCCTCCGGAGCGGCGCCTTCACGGCGCGTGGAAATTCTAACTACTTCCCCGACTACTTCATCAGG
AACATCTCTGGTGTTCCTCTCGTCGTCCGCAACGAGGACCTCCGCCGTCCACTGCACTACAACGAGATCAGGAAC
```

ATCGCCTCTCCGTCCGGGACGCCCGGAGGTGCAAGGGCGTACATGGTGAGCGTCCATAACAGGAAGAACAACATC
CACGCTGTGCATGAGAACGGCTCCATGATCCACCTGGCGCCCAATGATTACACCGGCTTCACCATCTCTCCAATC
CACGCCACCCAAGTGAACAACCAGACACGCACCTTCATCTCCGAGAAGTTCGGCAACCAGGGCGACTCCCTGAGG
TTCGAGCAGAACAACACCACCGCCAGGTACACCCTGCGCGGCAACGGCAACAGCTACAACCTGTACCTGCGCGTC
AGCTCCATTGGCAACTCCACCATCAGGGTCACCATCAACGGGAGGGTGTACACAGCCACCAATGTGAACACGACG
ACCAACAATGATGGCGTCAACGACAACGGCGCCCGCTTCAGCGACATCAACATTGGCAACGTGGTGGCCAGCAGC
AACTCCGACGTCCCGCTGGACATCAACGTGACCCTGAACTCTGGCACCCAGTTCGACCTCATGAACATCATGCTG
GTGCCAACTAACATCTCGCCGCTGTACTGATAGGAGCTCTGATCCCCATGGGA

>lcl|DI362404 MON89034|T-nos|10746-11012
ATTCCCGATCGTTCAAACATTTGGCAATAAAGTTTCTTAAGATTGAATCCTGTTGCCGGTCTTGCGATGATTATC
ATATAATTTCTGTTGAATTACGTTAAGCATGTAATAATTAACATGTAATGCATGACGTTATTTATGAGATGGGTT
TTTATGATTAGAGTCCCGCAATTATACATTTAATACGCGATAGAAAACAAAATATAGCGCGCAAACTAGGATAAA
TTATCGCGCGCGGTGTCATCTATGTTACTAGATCGGGGATAT

>lcl|DI362404 MON89034|3'Maize|11388-12208
AAAGTTTCTTCATGGCACATCTAGCTTTTGCCTAATACCATGTAGGGCTACATCTTAAAAATCTATACTACTATA
TTAAAGCTGCAGGGGTAGCCTGTCTCCACCTGGTTCTGCCTCGAGCCAATCTAAACCGTCCATCTATATCCATCA
AATCAGCACCGTCCGGTCCGTGCGCACCTCCTCTCCCGCTATTCAGTTGCATACTTGCAGCAGGTTCTCCCTCCT
CACCATTTCCTCTGCCTCCTCTCTCGCTCACTGGTCAGATTCATCCTGCCTCTCCCGCATGCGCTCCCTCCCCAT
GCCCCGTCTCGCACTATCGCCACACCTCACCGCGGGGAGACGAAGACGGTGGACGCATCCTCACCTCCTCCGCTA
GTTGTCGCTCTTCCATCCTCTTCAACAACTTCTACATAGGGAGAGGCGGTTCGGCGTCCCGACGCCGCCGCTTCT
CCCCTCCCCATGGAGGACGAGAACATCGACCTCGGCGGCGGGGGCGATGCCTCCGCTCTGCATAGAGGAGGGTTG
TAGTGGCAAGCAGCAATGCCAACACCGAGGCGGGCCAAGACTAGGCAACAATAGGACGGCACGCCCGGTTGTCAG
CGAGGTGGCGGCATCGTGTGCCGCTACCGAACAACATCTCCGGCGCTGGAGTCGGTGAGTTACTGCGCCACCCGG
ACGCCCTCAATGCACTGATATCTACCCGGTCTCCATCGCCGCCCTTCCTCCCTTCCCTCTCCCTGTGCCTCCCTC
TCTTGCCCTCTCCCTTCCAACTGCTCCCGCCCCAGCCCTAGCCCAACCACCTCCCGCGCAGGGTCACCAA

>lcl|HV702026 MON88017|5'Maize|1-103
ACCCGATCAGAGCGCTAAGCAGCAGAATCGTGTGACAACGCTAGCAGCTCTCCTCCAACACATCATCGACAAGCA
CCTTTTTTGCCGGAGTATGACGGTGACG

>lcl|HV702026 MON88017|P-ract|409-1814
GCTTACTCGAGGTCATTCATATGCTTGAGAAGAGAGTCGGGATAGTCCAAAATAAAACAAAGGTAAGATTACCTG
GTCAAAAGTGAAAACATCAGTTAAAAGGTGGTATAAAGTAAAATATCGGTAATAAAAGGTGGCCCAAAGTGAAAT
TTACTCTTTTCTACTATTATAAAAATTGAGGATGTTTTTGTCGGTACTTTGATACGTCATTTTTGTATGAATTGG
TTTTTAAGTTTATTCGCTTTTGGAAATGCATATCTGTATTTGAGTCGGGTTTTAAGTTCGTTTGCTTTTGTAAAT
ACAGAGGGATTTGTATAAGAAATATCTTTAGAAAAACCCATATGCTAATTTGACATAATTTTTGAGAAAAATATA
TATTCAGGCGAATTCTCACAATGAACAATAATAAGATTAAAATAGCTTTCCCCCGTTGCAGCGCATGGGTATTTT
TTCTAGTAAAAATAAAAGATAAACTTAGACTCAAAACATTTACAAAAACAACCCCTAAAGTTCCTAAAGCCCAAA
GTGCTATCCACGATCCATAGCAAGCCCAGCCCAACCCAACCCAACCCAACCCACCCCAGTCCAGCCAACTGGACA
ATAGTCTCCACACCCCCCCACTATCACCGTGAGTTGTCCGCACGCACCGCACGTCTCGCAGCCAAAAAAAAAAAG
AAAGAAAAAAAAGAAAAAGAAAAAAACAGCAGGTGGGTCCGGGTCGTGGGGGCCGGAAACGCGAGGAGGATCGCGA
GCCAGCGACGAGGCCGGCCCTCCCTCCGCTTCCAAAGAAACGCCCCCCATCGCCACTATATACATACCCCCCCCT
CTCCTCCCATCCCCCCAACCCTACCACCACCACCACCACCACCTCCACCTCCTCCCCCCTCGCTGCCGGACGACG
AGCTCCTCCCCCCTCCCCCTCCGCCGCCGCCGCGCCGGTAACCACCCCGCCCCTCTCCTCTTTCTTTCTCCGTTT
TTTTTTCCGTCTCGGTCTCGATCTTTGGCCTTGGTAGTTTGGGTGGGCGAGAGGCGGCTTCGTGCGCGCCCAGAT
CGGTGCGCGGGAGGGGCGGGATCTCGCGGCTGGGGCTCTCGCCGGCGTGGATCCGGCCCGGATCTCGCGGGGAAT
GGGGCTCTCGGATGTAGATCTGCGATCCGCCGTTGTTGGGGGAGATGATGGGGGGTTTAAAATTTCCGCCGTGCT
AAACAAGATCAGGAAGAGGGGAAAAGGGCACTATGGTTTATATTTTTATATATTTCTGCTGCTTCGTCAGGCTTA
GATGTGCTAGATCTTTCTTTCTTCTTTTTGTGGGTAGAATTTGAATCCCTCAGCATTGTTCATCGGTAGTTTTTC
TTTTCATGATTTGTGACAAATGCAGCCTCGTGCGGAGCTTTTTTGTAGGTAGAAGT

>lcl|HV702026 MON88017|At_CTP2|1815-2048
GATCAACCATGGCGCAAGTTAGCAGAATCTGCAATGGTGTGCAGAACCCATCTCTTATCTCCAATCTCTCGAAAT
CCAGTCAACGCAAATCTCCCTTATCGGTTTCTCTGAAGACGCAGCAGCATCCACGAGCTTATCCGATTTCGTCGT
CGTGGGGATTGAAGAAGAGTGGGATGACGTTAATTGGCTCTGAGCTTCGTCCTCTTAAGGTCATGTCTTCTGTTT
CCACGGCGT

>lcl|HV702026 MON88017|cp4_epsps|2049-3420
GCATGCTTCACGGTGCAAGCAGCCGGCCCGCAACCGCCCGCAAATCCTCTGGCCTTTCCGGAACCGTCCGCATTC
CCGGCGACAAGTCGATCTCCCACCGGTCCTTCATGTTCGGCGGTCTCGCGAGCGGTGAAACGCGCATCACCGGCC
TTCTGGAAGGCGAGGACGTCATCAATACGGGCAAGGCCATGCAGGCGATGGGCGCCCGCATCCGTAAGGAAGGCG
ACACCTGGATCATCGATGGCGTCGGCAATGGCGGCCTCCTGGCGCCTGAGGCGCCGCTCGATTTCGGCAATGCCG
CCACGGGCTGCCGCCTGACGATGGGCCTCGTCGGGGTCTACGATTTCGACAGCACCTTCATCGGCGACGCCTCGC
TCACAAAGCGCCCGATGGGCCGCGTGTTGAACCCGCTGCGCGAAATGGGCGTGCAGGTGAAATCGGAAGACGGTG
ACCGTCTTCCCGTTACCTTGCGCGGGCCGAAGACGCCGACGCCGATCACCTACCGCGTGCCGATGGCCTCCGCAC
AGGTGAAGTCCGCCGTGCTGCTCGCCGGCCTCAACACGCCCGGCATCACGACGGTCATCGAGCCGATCATGACGC
GCGATCATACGGAAAAGATGCTGCAGGGCTTTGGCGCCAACCTTACCGTCGAGACGGATGCGGACGGCGTGCGCA
CCATCCGCCTGGAAGGCCGCGGCAAGCTCACCGGCCAAGTCATCGACGTGCCGGGCGACCCGTCCTCGACGGCCT
TCCCGCTGGTTGCGGCCCTGCTTGTTCCGGGCTCCGACGTCACCATCCTCAACGTGCTGATGAACCCCACCCGCA
CCGGCCTCATCCTGACGCTGCAGGAAATGGGCGCCGACATCGAAGTCATCAACCCGCGCCTTGCCGGCGGCGAAG
ACGTGGCGGACCTGCGCGTTCGCTCCTCCACGCTGAAGGGCGTCACGGTGCCGGAAGACCGCGCGCCTTCGATGA
TCGACGAATATCCGATTCTCGCTGTCGCCGCCGCCTTCGCGGAAGGGGCGACCGTGATGAACGGTCTGGAAGAAC
TCCGCGTCAAGGAAAGCGACCGCCTCTCGGCCGTCGCCAATGGCCTCAAGCTCAATGGCGTGGATTGCGATGAGG
GCGAGACGTCGCTCGTCGTGCGTGGCCGCCCTGACGGCAAGGGGCTCGGCAACGCCTCGGGCGCCGCCGTCGCCA
CCCATCTCGATCACCGCATCGCCATGAGCTTCCTCGTCATGGGCCTCGTGTCGGAAAACCCTGTCACGGTGGACG
ATGCCACGATGATCGCCACGAGCTTCCCGGAGTTCATGGACCTGATGGCCGGGCTGGGCGCGAAGATCGAACTCT
CCGATACGAAGGCTGCCTGATG

>lcl|HV702026 MON88017|NOS|3421-3694
AGCTCGAATTCCCGATCGTTCAAACATTTGGCAATAAAGTTTCTTAAGATTGAATCCTGTTGCCGGTCTTGCGAT
GATTATCATATAATTTCTGTTGAATTACGTTAAGCATGTAATAATTAACATGTAATGCATGACGTTATTTATGAG
ATGGGTTTTTATGATTAGAGTCCCGCAATTATACATTTAATACGCGATAGAAAACAAAATATAGCGCGCAAACTA
GGATAAATTATCGCGCGCGGTGTCATCTATGTTACTAGATCGGGGATTT

>lcl|HV702026 MON88017|P-35S|3695-4361
GCGGCCGCGTTAACAAGCTTCTGCAGGTCCGATTGAGACTTTTCAACAAAGGGTAATATCCGGAAACCTCCTCGG
ATTCCATTGCCCAGCTATCTGTCACTTTATTGTGAAGATAGTGGAAAAGGAAGGTGGCTCCTACAAATGCCATCA
TTGCGATAAAGGAAAGGCCATCGTTGAAGATGCCTCTGCCGACAGTGGTCCCAAAGATGGACCCCCACCCACGAG
GAGCATCGTGGAAAAAGAAGACGTTCCAACCACGTCTTCAAAGCAAGTGGATTGATGTGATGGTCCGATTGAGAC
TTTTCAACAAAGGGTAATATCCGGAAACCTCCTCGGATTCCATTGCCCAGCTATCTGTCACTTTATTGTGAAGAT
AGTGGAAAAGGAAGGTGGCTCCTACAAATGCCATCATTGCGATAAAGGAAAGGCCATCGTTGAAGATGCCTCTGC
CGACAGTGGTCCCAAAGATGGACCCCCACCCACGAGGAGCATCGTGGAAAAAGAAGACGTTCCAACCACGTCTTC
AAAGCAAGTGGATTGATGTGATATCTCCACTGACGTAAGGGATGACGCACAATCCCACTATCCTTCGCAAGACCC
TTCCTCTATATAAGGAAGTTCATTTCATTTGGAGAGGACACGCTGACAAGCTGACTCTAGCAGATCC

>lcl|HV702026 MON88017|wtCAB_leader|4362-4440
TCTAGAACCATCTTCCACACACTCAAGCCACACTATTGGAGAACACACAGGGACAACACACCATAAGATCCAAGG
GAGG

>lcl|HV702026 MON88017|ract_intron|4441-4924
CCTCCGCCGCCGCCGGTAACCACCCCGCCCCTCTCCTCTTTCTTTCTCCGTTTTTTTTTCCGTCTCGGTCTCGAT
CTTTGGCCTTGGTAGTTTGGGTGGGCGAGAGGCGGCTTCGTGCGCGCCCAGATCGGTGCGCGGGAGGGGCGGGAT
CTCGCGGCTGGGGCTCTCGCCGGCGTGGATCCGGCCCGGATCTCGCGGGGAATGGGGCTCTCGGATGTAGATCTG
CGATCCGCCGTTGTTGGGGGAGATGATGGGGGGTTTAAAATTTCCGCCGTGCTAAACAAGATCAGGAAGAGGGGA

AAAGGGCACTATGGTTTATATTTTTATATATTTCTGCTGCTTCGTCAGGCTTAGATGTGCTAGATCTTTCTTTCT
TCTTTTTGTGGGTAGAATTTGAATCCCTCAGCATTGTTCATCGGTAGTTTTTCTTTTCATGATTTGTGACAAATG
CAGCCTCGTGCGGAGCTTTTTTGTAGGTAGAAGT

>lcl|HV702026 MON88017|cry3Bb1|4925-6914
GATCAACCATGGCCAACCCCAACAATCGCTCCGAGCACGACACGATCAAGGTCACCCCCAACTCCGAGCTCCAGA
CCAACCACAACCAGTACCCGCTGGCCGACAACCCCAACTCCACCCTGGAAGAGCTGAACTACAAGGAGTTCCTGC
GCATGACCGAGGACTCCTCCACGGAGGTCCTGGACAACTCCACCGTCAAGGACGCCGTCGGGACCGGCATCTCCG
TCGTTGGGCAGATCCTGGGCGTCGTTGGCGTCCCCTTCGCAGGTGCTCTCACCTCCTTCTACCAGTCCTTCCTGA
ACACCATCTGGCCCTCCGACGCCGACCCCTGGAAGGCCTTCATGGCCCAAGTCGAAGTCCTGATCGACAAGAAGA
TCGAGGAGTACGCCAAGTCCAAGGCCCTGGCCGAGCTGCAAGGCCTGCAAAACAACTTCGAGGACTACGTCAACG
CGCTGAACTCCTGGAAGAAGACGCCTCTGTCCCTGCGCTCCAAGCGCTCCCAGGACCGCATCCGCGAGCTGTTCT
CCCAGGCCGAGTCCCACTTCCGCAACTCCATGCCGTCCTTCGCCGTCTCCAAGTTCGAGGTCCTGTTCCTGCCCA
CCTACGCCCAGGCTGCCAACACCCACCTCCTGTTGCTGAAGGACGCCCAGGTCTTCGGCGAGGAATGGGGCTACT
CCTCGGAGGACGTCGCCGAGTTCTACCGTCGCCAGCTGAAGCTGACCCAACAGTACACCGACCACTGCGTCAACT
GGTACAACGTCGGCCTGAACGGCCTGAGGGGCTCCACCTACGACGCATGGGTCAAGTTCAACCGCTTCCGCAGGG
AGATGACCCTGACCGTCCTGGACCTGATCGTCCTGTTCCCCTTCTACGACATCCGCCTGTACTCCAAGGGCGTCA
AGACCGAGCTGACCCGCGACATCTTCACGGACCCCATCTTCCTGCTCACGACCCTCCAGAAGTACGGTCCCACCT
TCCTGTCCATCGAGAACTCCATCCGCAAGCCCCACCTGTTCGACTACCTCCAGGGCATCGAGTTCCACACGCGCC
TGAGGCCAGGCTACTTCGGCAAGGACTCCTTCAACTACTGGTCCGGCAACTACGTCGAGACCAGGCCCTCCATCG
GCTCCTCGAAGACGATCACCTCCCCTTTCTACGGCGACAAGTCCACCGAGCCCGTCCAGAAGCTGTCCTTCGACG
GCCAGAAGGTCTACCGCACCATCGCCAACACCGACGTCGCGGCTTGGCCGAACGGCAAGGTCTACCTGGGCGTCA
CGAAGGTCGACTTCTCCCAGTACGATGACCAGAAGAACGAGACCTCCACCCAGACCTACGACTCCAAGCGCAACA
ATGGCCACGTCTCCGCCCAGGACTCCATCGACCAGCTGCCGCCTGAGACCACTGACGAGCCCCTGGAGAAGGCCT
ACTCCCACCAGCTGAACTACGCGGAGTGCTTCCTGATGCAAGACCGCAGGGGCACCATCCCCTTCTTCACCTGGA
CCCACCGCTCCGTCGACTTCTTCAACACCATCGACGCCGAGAAGATCACCCAGCTGCCCGTGGTCAAGGCCTACG
CCCTGTCCTCGGGTGCCTCCATCATTGAGGGTCCAGGCTTCACCGGTGGCAACCTGCTGTTCCTGAAGGAGTCCT
CGAACTCCATCGCCAAGTTCAAGGTCACCCTGAACTCCGCTGCCTTGCTGCAACGCTACCGCGTCCGCATCCGCT
ACGCCTCCACCACGAACCTGCGCCTGTTCGTCCAGAACTCCAACAATGACTTCCTGGTCATCTACATCAACAAGA
CCATGAACAAGGACGATGACCTGACCTACCAGACCTTCGACCTCGCCACCACGAACTCCAACATGGGCTTCTCGG
GCGACAAGAATGAACTGATCATTGGTGCTGAGTCCTTCGTCTCCAACGAGAAGATCTACATCGACAAGATCGAGT
TCATCCCCGTCCAGCTGTGATAGGAACTCTGATTGAATTC

>lcl|HV702026 MON88017|tahsp17|6915-7124
TGCATGCGTTTGGACGTATGCTCATTCAGGTTGGAGCCAATTTGGTTGATGTGTGTGCGAGTTCTTGCGAGTCTG
ATGAGACATCTCTGTATTGTGTTTCTTTCCCCAGTGTTTTCTGTACTTGTGTAATCGGCTAATCGCCAACAGATT
CGGCGATGAATAAATGAGAAATAAATTGTTCTGATTTTGAGTGCAAAAAAAAAGGAATTA

>lcl|HV702026 MON88017|3'Maize|7230-7450
GAGTCGGGTTTGGATGGTCAACTCCGGCATACTGCCGAAAACAAACCAATCCGTCACCGTCAAGGCCCCGCACCG
CTGGCCGCACGCAGGAAAAATAAGTTGCGACCGCGAGCGGGCGAATCAGAAAGGGCGTCCGGCCTTGGTCAGACA
CGACAGCGACGCGGAAAGGCTGCGCCCGCGGTGCCATCTACAAGGGTCCACGTCCATCCAAAAAGAGCGG

>lcl|AX600188 MON15985|5'Cotton|1-499
GTTCGAGTGAGAGCATGCCTGTCGGGACCCGAAAGATGGTGAACTATGCCTGAGCGGGGCGAAGCCAGAGGAAAC
TCTGGTGGAGGCCCGCAGCGATACTGACGTGCAAATCGTTCGTCTGACTTGGGTATAGGGGCGAAAGACTAATCG
AACCGTCTAGTAGCTGGTTCCCTCCGAAGTTTCCCTCAGGATAGCTGGAGCCCTTAGCGAGTTCTATCGGGTAAA
GCCAATGATTAGAGGCATCGGGGGCGCAACGCCCTCGACCTATTCTCAAACTTTAAATAGGTAGGACGGCGCGGC
TGCTTCGTTGAGCCGCGCCACGGAATCGAGAGCTCCAAGTGGGCCATTTTTGGTAAGCAGAACTGGCGATGCGGG
ATGAACCGGAAGCCGGGTTACGGTGCCCAACTGCGCGCTAACCTAGAACCCACAAAGGGTGTTGGTCGATTAAGA
CAGCAGGACGGTGGTCATGGAAGTCGAAATCCGCTAAGGAGTGTGTAAC

>lcl|AX600188 MON15985|7S3'UTR|570-741

TCAATATTGTGGCAGGACATTGCTACATGATACCTCTTAGAATTGTTTAGACTTCAGATCGATCTTGTCAGTCTG
AAAGACCCAAAAACAAATGCAATTTCTTTTCTGGTAGACCGTGACAATTTGTCTAAGATGTATCTGATTTAATGC
CTTTTGTATATAATACACTCAC

>lcl|AX600188 MON15985|3'Cotton|742-1121
CTGCCGAATCAACTAGCCCCGAAAATGGATGGCGCTTAAGCGCGCGACCTATACCCGGCCGTCGGGGCAAGGGCC
AGGCCCCGATGAGTAGGAGGGCGCGGCGGTCGCCGCAAAACCCGGGGCGCGAGCCCGGGCGGAGCGGCCGTCGGT
GCAGATCTTGGTGGTAGTAGCAAATATTCAAATGAGAACTTTGAAGGCCGAAGAGGGGAAAGGTTCCATGTGAAC
GGCACTTGCACATGGGTTAGTCGATCCTAAGAGACGGGGGAAGCCCGTCCGACAGCGCGTCCAGCGCGAGCTTCG
AAAGGGAATCGGGTTAAAATTCCTGAACCGGGACGCGGCGGCTGACGGCAACGTTAGGGAGTCCGGAGACATCTG
AAGC

>lcl|AX600170 MON15985|5'Cotton|1-309
AGCAAGTTTCAAACCCAGAATTGAACATTGAATTCCTTCCCACTCGATCAACCAATAACATACTTGGTTTATATA
TATATAAATGAGTCCCCTATTCTATTTCCTCCATTCCCCCCCCCCTCCCCGGTACGGATGAGTAGGCCTACTCTT
TTTTTCATAGTTGTTTTTTAGCCGATTTAAGAGATGAAAATTCAGACAATGCAATAGGGAAGGTCATTGATGTAC
ACCAAAGAGAAACCCCAATCATAAAAGTATATGCGACAAAACGCGTTGAAATTAAAAACCAATGCCACCCCACTG
ACCCACTTA

>lcl|AX600170 MON15985|Partial_cry1Ac|310-1201
GCAGAGAAGAAGTGGAGGGACAAACGTGAGAAACTCGAATGGGAAACTAACATCGTTTACAAGGAGGCCAAAGAG
TCCGTGGATGCTTTGTTCGTGAACTCCCAATATGATCAGTTGCAAGCCGACACCAACATCGCCATGATCCACGCC
GCAGACAAACGTGTGCACAGCATTCGTGAGGCTTACTTGCCTGAGTTGTCCGTGATCCCTGGTGTGAACGCTGCC
ATCTTCGAGGAACTTGAGGGACGTATCTTTACCGCATTCTCCTTGTACGATGCCAGAAACGTCATCAAGAACGGT
GACTTCAACAATGGCCTCAGCTGCTGGAATGTGAAAGGTCATGTGGACGTGGAGGAACAGAACAATCAGCGTTCC
GTCCTGGTTGTGCCTGAGTGGGAAGCTGAAGTGTCCCAAGAGGTTAGAGTCTGTCCAGGTAGAGGCTACATTCTC
CGTGTGACCGCTTACAAGGAGGGATACGGTGAGGGTTGCGTGACCATCCACGAGATCGAGAACAACACCGACGAG
CTTAAGTTCTCCAACTGCGTCGAGGAAGAAATCTATCCCAACAACACCGTTACTTGCAACGACTACACTGTGAAT
CAGGAAGAGTACGGAGGTGCCTACACTAGCCGTAACAGAGGTTACAACGAAGCTCCTTCCGTTCCTGCTGACTAT
GCCTCCGTGTACGAGGAGAAATCCTACACAGATGGCAGACGTGAGAACCCTTGCGAGTTCAACAGAGGTTACAGG
GACTACACACCACTTCCAGTTGGCTATGTTACCAAGGAGCTTGAGTACTTTCCTGAGACCGACAAAGTGTGGATC
GAGATCGGTGAAACCGAGGGAACCTTCATCGTGGACAGCGTGGAGCTTCTCTTGATGGAGGAATAAT

>lcl|AX600170 MON15985|Partial_7S3'UTR|1243-1413
CCGTCCTTTGTCTTCAATTTTGAGGGCTTTTTACTGAATAAGTATGTAGTACTAAAATGTATGCTGTAATAGCTC
ATAGTGAGCGAGGAAAGTATCGGGCTATTTAACTATGACTTGAGCTCCATCTATGAATAAATAAATCAGCATATG
ATGCTTTTGTTTTGTGTACT

>lcl|AX600169 MON15985|OriV|1-378
CCCTCAAATGTCAATAGGTGCGCCCCTCATCTGTCAGCACTCTGCCCCTCAAGTGTCAAGGATCGCGCCCCTCAT
CTGTCAGTAGTCGCGCCCCTCAAGTGTCAATACCGCAGGGCACTTATCCCCAGGCTTGTCCACATCATCTGTGGG
AAACTCGCGTAAAATCAGGCGTTTTCGCCGATTTGCGAGGCTGGCCAGCTCCACGTCGCCGGCCGAAATCGAGCC
TGCCCCTCATCTGTCAACGCCGCGCCGGGTGAGTCGGCCCCTCAAGTGTCAACGTCCGCCCCTCATCTGTCAGTG
AGGGCCAAGTTTTCCGCGAGGTATCCACAACGCCGGCGGCCGCGGTGTCTCGCACACGGCTTCGACGGCGTTTCT
GGT

>lcl|KJ608138 MON15985|CaMV35S_promotor|1-610
TTGAGACTTTTCAACAAAGGGTAATATCCGGAAACCTCCTCGGATTCCATTGCCCAGCTATCTGTCACTTTATTG
TGAAGATAGTGGAAAAGGAAGGTGGCTCCTACAAATGCCATCATTGCGATAAAGGAAAGGCCATCGTTGAAGATG
CCTCTGCCGACAGTGGTCCCAAAGATGGACCCCCACCCACGAGGAGCATCGTGGAAAAAGAAGACGTTCCAACCA
CGTCTTCAAAGCAAGTGGATTGATGTGATGGTCCGATTGAGACTTTTCAACAAAGGGTAATATCCGGAAACCTCC
TCGGATTCCATTGCCCAGCTATCTGTCACTTTATTGTGAAGATAGTGGAAAAGGAAGGTGGCTCCTACAAATGCC
ATCATTGCGATAAAGGAAAGGCCATCGTTGAAGATGCCTCTGCCGACAGTGGTCCCAAAGATGGACCCCCACCCA

CGAGGAGCATCGTGGAAAAAGAAGACGTTCCAACCACGTCTTCAAAGCAAGTGGATTGATGTGATATCTCCACTG
ACGTAAGGGATGACGCACAATCCCACTATCCTTCGCAAGACCCTTCCTCTATATAAGGAAGTTCATTTCATTTGG
AGAGGACAC

>lcl|KJ608146 MON15985|CaMV35S_promotor|1-322
TACCTGTCACTTCATCAAAAGGACAGTAGAAAAGGAAGGTGGCACCTACAAATGCCATCATTGCGATAAAGGAAA
GGCTATCATTCAAGATGCCTCTGCCGACAGTGGTCCCAAAGATGGACCCCCACCCACGAGGAGCATCGTGGAAAA
AGAAGACGTTCCAACCACGTCTTCAAAGCAAGTGGATTGATGTGATACTTCCACTGACGTAAGGGATGACGCACA
ATCCCACTATCCTTCGCAAGACCCTTCCTCTATATAAGGAAGTTCATTTCATTTGGAGAGGACACGCTGAAATCA
CCAGTCTCTCTCTACAAGATC

>lcl|KJ608145 MON15985|CaMV35S_promotor|1-615
TTGAGACTTTTCAACAAAGGGTAATATCCGGAAACCTCCTCGGATTCCATTGCCCAGCTATCTGTCACTTTATTG
TGAAGATAGTGGAAAAGGAAGGTGGCTCCTACAAATGCCATCATTGCGATAAAGGAAAGGCCATCGTTGAAGATG
CCTCTGCCGACAGTGGTCCCAAAGATGGACCCCCACCCACGAGGAGCATCGTGGAAAAAGAAGACGTTCCAACCA
CGTCTTCAAAGCAAGTGGATTGATGTGATGGTCCGATTGAGACTTTTCAACAAAGGGTAATATCCGGAAACCTCC
TCGGATTCCATTGCCCAGCTATCTGTCACTTTATTGTGAAGATAGTGGAAAAGGAAGGTGGCTCCTACAAATGCC
ATCATTGCGATAAAGGAAAGGCCATCGTTGAAGATGCCTCTGCCGACAGTGGTCCCAAAGATGGACCCCCACCCA
CGAGGAGCATCGTGGAAAAAGAAGACGTTCCAACCACGTCTTCAAAGCAAGTGGATTGATGTGATATCTCCACTG
ACGTAAGGGATGACGCACAATCCCACTATCCTTCGCAAGACCCTTCCTCTATATAAGGAAGTTCATTTCATTTGG
AGAGGACACGCTGA

>lcl|EA135634 MON15985|NOS|49-315
AATTCCCGATCGTTCAAACATTTGGCAATAAAGTTTCTTAAGATTGAATCCTGTTGCCGGTCTTGCGATGATTAT
CATATAATTTCTGTTGAATTACGTTAAGCATGTAATAATTAACATGTAATGCATGACGTTATTTATGAGATGGGT
TTTTATGATTAGAGTCCCGCAATTATACATTTAATACGCGATAGAAAACAAAATATAGCGCGCAAACTAGGATAA
ATTATCGCGCGCGGTGTCATCTATGTTACTAGATCGGGGATA

>lcl|EA135634 MON15985|Cotton|350-1160
GCACTGAAATCCCATCCATTTAGCAACCTTAAAAGTAATAAACTTAAACTAAGTGTACTCCTTATCAAAACATAA
ACCAAGAGATGAGGATAACAGCGTTCGGCCAACCCCAATACCACCTATAATCAAAGCATGCGATGTCACGAACGT
TTTCCTGACGGCAATCGACACAAAGAAGAGATTAAAAAGGGAGACCACTATAGAAAGAAGAAAGAGAATCGGCAC
CACTGTGAAACTACATTTGATAACCGTATTAGCAAGGCTCCACCATTGATAAAAACTCGACAACCAAGAGGAACT
CACTTACCTTGATCAAAGTAGGGTTGTTGGTACTAAGGCTCAAAGCAAGGAAAATGTCTAAATGAGTGAGTTGGG
CTTTTTTAATTTAATTAAACTGAAAAATAAGAAAGGATGTTGCCAATTTTGTGTGGCAAGGACGGACAAGAGGTT
TCGACCCAACCTAGAGAAGACATTAAAACTGCAGAACTGGGCTCGCTTGTTCAGGATCGGGCTTTAAAGGAGAAT
GGTTTGATGGACCAGAATAGAGACAAACCAATACACTCAGATTTTGATCTTCCTTATTCTCCCTCAACTATTGAT
TTTGTTTTGGGTAATGATGGTGTAGCAGGGGATCGGGTTTTAGGTGTTTTTCAAGGCATGACTGTTACTCATTTT
AATCCGACTTGCGATGACCATAATGAGATGGAGGCTGTACTGAAGGATGAAGTATTGGATCCAAATAGGCATTCG
ACATGTTTGATAAAACTCTTAGTTACAAAATTCTTAGTAAACGAATCCATAAGGTTATCTT

>lcl|EA135633 MON15985|5'Cotton|1-361
TTGTCATTGGTGCAGACAGGTTCGGGGAGTATGGCCTATGGGTGGTGGTAGAGAGAAAGTAACAGCGAAATCTTA
GGGTGGTCCGAGAAATGAGATTGTTAAGTCCTGATTTAATGTGCTGAGTTCAGTTGATGGAGAGGAAAATGGCAA
AGATTTGAGGGGAGAGGATTTTGTGGATCCTAACATTAAACAGAATCGTTCTACTAAAAGGGGTAAGGGTAGTGG
TGGAAGAGATATTTTTAAAAAAGCTAAGGTAAAATCTGGGCAAAGTTTTAACGTGGCCCAGGGGAATAAAAAAGG
GACTATAGGGCCTAGTGGTAGTCAACAGGCCGGGCGGGAAAGTATTCATTTGAAATGGGTT

>lcl|EA135633 MON15985|Chloroplast|362-750
TATTCGTATTAACAGCTACTCTAGGAGGAATGTTTTTATGCGGTGCTAACGATTTAATAACTATCTTTGTAGCTC
CAGAATGTTTCAGTTTATGCTCCTACCTATTATCTGGATATACCAAGAAAGATGTACGGTCTAATGAGGCTACTA
CGAAATATTTACTCATGGGTGGGGCAAGCTCTTCTATTCTGGTTCATGGTTTCTCTTGGCTATATGGTTCATCCG
GGGGAGAGATCGAGCTTCAAGAAATAGTGAATGGTCTTATCAATACACAAATGTATAACTCCCCAGGAATTTCAA

TTGCGCTTATATTCATCACTGTAGGAATTGGGTTCAAGCTTTCCCCAGCCCCTTCTCATCAATGGACTCCTGACG
TATACGAAGGAGTT

>lcl|EA135633 MON15985|Cotton|751-1885
CTTTTAGGCTTTTGCATTTTGTTGAAAATTCTTATTTTCACAGGACTTTGCTTATCATAATTATTGACAGCCAAC
TGGTGAAAATGGTGCCACGAGAGCTCCCATCAGCATTGATTTGGCTAGGGATAGTTCTTTAAGTGCTAAGTCAAT
CATCTTGCAAATTGACAATAAATCTCAACAGGTTTCAGCAAGTAAGGCCTCCTTCATTATTTTGGTTGCTTCTTT
TTCATTAATGATGTTGCTTGTTTGCACCTGGAAAACATGCACCGTTCTTCATCATGCACCTTGGATATGGTCTTG
AGTGCTCTCTGACTGCCCGTCATCTTTCGAATTGGTGTCTGATTGAGTGCACCCGCCTAAGTTCAAGATGCCGGA
GTTTGAATAGTATAACGGGACTAGTTGTCTTGAGGCTCATATTACTATGTTCTGTCGATATAATCATCCATGGGC
TTCGCATCCACCATTGCAAGGCCCAACCACCTAGCACTGTGATGGGTCCAAACGCAAAGGTGATCCCTTTAGGCC
AAATGGATGGAGACGCTATAAGATTGGTCACCGCAAGAAAAGTGTGGATCGATAGGCCCTGTAGTTGATGATGGG
GATGATCACGATGACCCTGCCTATCCTCCAAGTTTCACCCCAACTAACATCCAAGTGCAACCAGAGGTGTACCCG
CAGAGGGCACCTATTACCATTAGATCGACTGCAAGTGTAGGTAATGTTGGACAGGGTAGATCAGGGTGTTCACGG
TGTGGTAGACTTTACTATGGTCCTTTTCGGGCAGGCGAAAATGTTTGTTATAAATGCGGCACTCCAGATCATTTT
GTACGAGAATGTCCAGAGGTGGCTAATCGAGAGAATCGAATCACTTACGAAAAAAGATTATTGTTACTAAGTTTT
CATGTTTTATGCCATGCGTAGCCATGTTTTTAATAAAGGGTTGAAGACTTGAATGACAAGTTAGCGTAAAGCATT
TTTTTTCTTTCATGTTGGCACATTTGATGCACTATGTCTTCGTCTATTTTTCAAACATACTGTTGGAAGAATTAT
GATTCACGTGTTGTTTAAGATCAAAAAGTGCATGCCTAACTAATACTTATCAGAAACAAATAATGCAATGAGTCA
TATCTCTATA

>lcl|EA135633 MON15985|P35S|1886-2267
AAGGGTAATATCCGGAAACCTCCTCGGATTCCATTGCCCAGCTATCTGTCACTTTATTGTGAAGATAGTGGAAAA
GGAAGGTGGCTCCTACAAATGCCATCATTGCGATAAAGGAAAGGCCATCGTTGAAGATGCCTCTGCCGACAGTGG
TCCCAAAGATGGACCCCCACCCACGAGGAGCATCGTGGAAAAAGAAGACGTTCCAACCACGTCTTCAAAGCAAGT
GGATTGATATGATATCTCCACTGACGTAAGGGATGACGCACAATCCCACTATCCTTCGCAAGACCCTTCCTCTAT
ATAAGGAAGTTCATTTCATTTGGAGAGGACACGCTGACAAGCTGACTCTAGCAGATCTCCATGGTCCGTCCTGTA
GAAACC

>lcl|X06613 Arabidopsis|At_CTP2|379-603
TGGCGCAAGTTAGCAGAATCTGCAATGGTGTGCAGAACCCATCTCTTATCTCCAATCTCTCGAAATCCAGTCAAC
GCAAATCTCCCTTATCGGTTTCTCTGAAGACGCAGCAGCATCCACGAGCTTATCCGATTTCGTCGTCGTGGGGAT
TGAAGAAGAGTGGGATGACGTTAATTGGCTCTGAGCTTCGTCCTCTTAAGGTCATGTCTTCTGTTTCCACGGCGG

>lcl|X06932 Petunia|Untranslated_leader_HSP70|143-239
CAGAAAAATTTGCTACATTGTTTCACAAACTTCAAATATTATTCATTTATTTGTCAGCTTTCAAACTCTTTGTTT
CTTGTTTGTTGATTGAGAATAA

>lcl|U12639 pBI101|UidA|2551-4359
TGTTACGTCCTGTAGAAACCCCAACCCGTGAAATCAAAAAACTCGACGGCCTGTGGGCATTCAGTCTGGATCGCG
AAAACTGTGGAATTGATCAGCGTTGGTGGGAAAGCGCGTTACAAGAAAGCCGGGCAATTGCTGTGCCAGGCAGTT
TTAACGATCAGTTCGCCGATGCAGATATTCGTAATTATGCGGGCAACGTCTGGTATCAGCGCGAAGTCTTTATAC
CGAAAGGTTGGGCAGGCCAGCGTATCGTGCTGCGTTTCGATGCGGTCACTCATTACGGCAAAGTGTGGGTCAATA
ATCAGGAAGTGATGGAGCATCAGGGCGGCTATACGCCATTTGAAGCCGATGTCACGCCGTATGTTATTGCCGGGA
AAAGTGTACGTATCACCGTTTGTGTGAACAACGAACTGAACTGGCAGACTATCCCGCCGGGAATGGTGATTACCG
ACGAAAACGGCAAGAAAAAGCAGTCTTACTTCCATGATTTCTTTAACTATGCCGGAATCCATCGCAGCGTAATGC
TCTACACCACGCCGAACACCTGGGTGGACGATATCACCGTGGTGACGCATGTCGCGCAAGACTGTAACCACGCGT
CTGTTGACTGGCAGGTGGTGGCCAATGGTGATGTCAGCGTTGAACTGCGTGATGCGGATCAACAGGTGGTTGCAA
CTGGACAAGGCACTAGCGGGACTTTGCAAGTGGTGAATCCGCACCTCTGGCAACCGGGTGAAGGTTATCTCTATG
AACTGTGCGTCACAGCCAAAAGCCAGACAGAGTGTGATATCTACCCGCTTCGCGTCGGCATCCGGTCAGTGGCAG
TGAAGGGCCAACAGTTCCTGATTAACCACAAACCGTTCTACTTTACTGGCTTTGGTCGTCATGAAGATGCGGACT
TACGTGGCAAAGGATTCGATAACGTGCTGATGGTGCACGACCACGCATTAATGGACTGGATTGGGGCCAACTCCT
ACCGTACCTCGCATTACCCTTACGCTGAAGAGATGCTCGACTGGGCAGATGAACATGGCATCGTGGTGATTGATG

AAACTGCTGCTGTCGGCTTTAACCTCTCTTTAGGCATTGGTTTCGAAGCGGGCAACAAGCCGAAAGAACTGTACA
GCGAAGAGGCAGTCAACGGGGAAACTCAGCAAGCGCACTTACAGGCGATTAAAGAGCTGATAGCGCGTGACAAAA
ACCACCCAAGCGTGGTGATGTGGAGTATTGCCAACGAACCGGATACCCGTCCGCAAGTGCACGGGAATATTTCGC
CACTGGCGGAAGCAACGCGTAAACTCGACCCGACGCGTCCGATCACCTGCGTCAATGTAATGTTCTGCGACGCTC
ACACCGATACCATCAGCGATCTCTTTGATGTGCTGTGCCTGAACCGTTATTACGGATGGTATGTCCAAAGCGGCG
ATTTGGAAACGGCAGAGAAGGTACTGGAAAAAGAACTTCTGGCCTGGCAGGAGAAACTGCATCAGCCGATTATCA
TCACCGAATACGGCGTGGATACGTTAGCCGGGCTGCACTCAATGTACACCGACATGTGGAGTGAAGAGTATCAGT
GTGCATGGCTGGATATGTATCACCGCGTCTTTGATCGCGTCAGCGCCGTCGTCGGTGAACAGGTATGGAATTTCG
CCGATTTTGCGACCTCGCAAGGCATATTGCGCGTTGGCGGTAACAAGAAAGGGATCTTCACTCGCGACCGCAAAC
CGAAGTCGGCGGCTTTTCTGCTGCAAAAACGCTGGACTGGCATGAACTTCGGTGAAAAACCGCAGCAGGGAGGCA
AACAATGAA

>lcl|X55416 Bacillus|cry2Ab|874-2775
TGAATAGTGTATTGAATAGCGGAAGAACTACTATTTGTGATGCGTATAATGTAGCGGCTCATGATCCATTTAGTT
TTCAACACAAATCATTAGATACCGTACAAAAGGAATGGACGGAGTGGAAAAAAAATAATCATAGTTTATACCTAG
ATCCTATTGTTGGAACTGTGGCTAGTTTTCTGTTAAAGAAAGTGGGGAGTCTTGTTGGAAAAAGGATACTAAGTG
AGTTACGGAATTTAATATTTCCTAGTGGTAGTACAAATCTAATGCAAGATATTTTAAGAGAGACAGAAAAATTCC
TGAATCAAAGACTTAATACAGACACTCTTGCCCGTGTAAATGCGGAATTGACAGGGCTGCAAGCAAATGTAGAAG
AGTTTAATCGACAAGTAGATAATTTTTTGAACCCTAACCGAAACGCTGTTCCTTTATCAATAACTTCTTCAGTTA
ATACAATGCAACAATTATTTCTAAATAGATTACCCCAGTTCCAGATGCAAGGATACCAACTGTTATTATTACCTT
TATTTGCACAGGCAGCCAATTTACATCTTTCTTTTATTAGAGATGTTATTCTAAATGCAGATGAATGGGGAATTT
CAGCAGCAACATTACGTACGTATCGAGATTACTTGAAAAATTATACAAGAGATTACTCTAACTATTGTATAAATA
CGTATCAAAGTGCGTTTAAAGGTTTAAACACTCGTTTACACGATATGTTAGAATTTAGAACATATATGTTTTTAA
ATGTATTTGAGTATGTATCTATCTGGTCGTTGTTTAAATATCAAAGTCTTCTAGTATCTTCCGGTGCTAATTTAT
ATGCAAGTGGTAGTGGACCACAGCAGACCCAATCATTTACTTCACAAGACTGGCCATTTTTATATTCTCTTTTCC
AAGTTAATTCAAATTATGTGTTAAATGGATTTAGTGGTGCTAGGCTTTCTAATACCTTCCCTAATATAGTTGGTT
TACCTGGTTCTACTACAACTCACGCATTGCTTGCTGCAAGGGTTAATTACAGTGGAGGAATTTCGTCTGGTGATA
TAGGTGCATCTCCGTTTAATCAAAATTTTAATTGTAGCACATTTCTCCCCCCATTGTTAACGCCATTTGTTAGGA
GTTGGCTAGATTCAGGTTCAGATCGGGAGGGCGTTGCCACCGTTACAAATTGGCAAACAGAATCCTTTGAGACAA
CTTTAGGGTTAAGGAGTGGTGCTTTTACAGCTCGCGGTAATTCAAACTATTTCCCAGATTATTTTATTCGTAATA
TTTCTGGAGTTCCTTTAGTTGTTAGAAATGAAGATTTAAGAAGACCGTTACACTATAATGAAATAAGAAATATAG
CAAGTCCTTCAGGAACACCTGGTGGAGCACGAGCTTATATGGTATCTGTGCATAACAGAAAAAATAATATCCATG
CTGTTCATGAAAATGGTTCTATGATTCATTTAGCGCCAAATGACTATACAGGATTTACTATTTCGCCGATACATG
CAACTCAAGTGAATAATCAAACACGAACATTTATTTCTGAAAAATTTGGAAATCAAGGTGATTCTTTAAGGTTTG
AACAAAACAACACGACAGCTCGTTATACGCTTAGAGGGAATGGAAATAGTTACAATCTTTATTTAAGAGTTTCTT
CAATAGGAAATTCCACTATTCGAGTTACTATAAACGGTAGGGTATATACTGCTACAAATGTTAATACTACTACAA
ATAACGATGGAGTTAATGATAATGGAGCTCGTTTTTCAGATATTAATATCGGTAATGTAGTAGCAAGTAGTAATT
CTGATGTACCATTAGATATAAATGTAACATTAAACTCCGGTACTCAATTTGATCTTATGAATATTATGCTTGTAC
CAACTAATATTTCACCACTTTATTAAA

>lcl|X03043 Escherichia|aadA|82-870
TGAGGGAAGCGGTGATCGCCGAAGTATCGACTCAACTATCAGAGGTAGTTGGCGTCATCGAGCGCCATCTCGAAC
CGACGTTGCTGGCCGTACATTTGTACGGCTCCGCAGTGGATGGCGGCCTGAAGCCACACAGTGATATTGATTTGC
TGGTTACGGTGACCGTAAGGCTTGATGAAACAACGCGGCGAGCTTTGATCAACGACCTTTTGGAAACTTCGGCTT
CCCCTGGAGAGAGCGAGATTCTCCGCGCTGTAGAAGTCACCATTGTTGTGCACGACGACATCATTCCGTGGCGTT
ATCCAGCTAAGCGCGAACTGCAATTTGGAGAATGGCAGCGCAATGACATTCTTGCAGGTATCTTCGAGCCAGCCA
CGATCGACATTGATCTGGCTATCTTGCTGACAAAAGCAAGAGAACATAGCGTTGCCTTGGTAGGTCCAGCGGCGG
AGGAACTCTTTGATCCGGTTCCTGAACAGGATCTATTTGAGGCGCTAAATGAAACCTTAACGCTATGGAACTCGC
CGCCCGACTGGGCTGGCGATGAGCGAAATGTAGTGCTTACGTTGTCCCGCATTTGGTACAGCGCAGTAACCGGCA
AAATCGCGCCGAAGGATGTCGCTGCCGACTGGGCAATGGAGCGCCTGCCGGCCCAGTATCAGCCCGTCATACTTG
AAGCTAGGCAGGCTTATCTTGGACAAGAAGATCGCTTGGCCTCGCGCGCAGATCAGTTGGAAGAATTTGTTCACT
ACGTGAAAGGCGAGATCACCAAGGTAGTCGGCAAATAAT

>lcl|AM887683 pICH14313|NPTII|3861-4658
TGGGGATTGAACAAGATGGATTGCACGCAGGTTCTCCGGCCGCTTGGGTGGAGAGGCTATTCGGCTATGACTGGG
CACAACAGACAATCGGCTGCTCTGATGCCGCCGTGTTCCGGCTGTCAGCGCAGGGGCGCCCGGTTCTTTTTGTCA
AGACCGACCTGTCCGGTGCCCTGAATGAACTCCAGGACGAGGCAGCGCGGCTATCGTGGCTGGCCACGACGGGCG
TTCCTTGCGCAGCTGTGCTCGACGTTGTCACTGAAGCGGGAAGGGACTGGCTGCTATTGGGCGAAGTGCCGGGGC
AGGATCTCCTGTCATCTCACCTTGCTCCTGCCGAGAAAGTATCCATCATGGCTGATGCAATGCGGCGGCTGCATA
CGCTTGATCCGGCTACCTGCCCATTCGACCACCAAGCGAAACATCGCATCGAGCGAGCACGTACTCGGATGGAAG
CCGGTCTTGTCGATCAGGATGATCTGGACGAAGAGCATCAGGGGCTCGCGCCAGCCGAACTGTTCGCCAGGCTCA
AGGCGCGCATGCCCGACGGCGAGGATCTCGTCGTGACACATGGCGATGCCTGCTTGCCGAATATCATGGTGGAAA
ATGGCCGCTTTTCTGGATTCATCGACTGTGGCCGGCTGGGTGTGGCGGACCGCTATCAGGACATAGCGTTGGCTA
CCCGTGATATTGCTGAAGAGCTTGGCGGCGAATGGGCTGACCGCTTCCTCGTGCTTTACGGTATCGCCGCTCCCG
ATTCGCAGCGCATCGCCTTCTATCGCCTTCTTGACGAGTTCTTCTGAG

>lcl|DQ666283 pRGK367|7S_UTR|3149-3521
TGTAGTACTAAAATGTATGCTGTAATAGCTCATAGTGAGCGAGGAAAGTATCGGGCTATTTAACTATGACTTGAG
CTCCATCTATGAATAAATAAATCAGCATATGATGCTTTTGTTTTGTGTACTTCAACTGTCTGCTTAGCTAATTTG
ATATGGTTGGCACTTGGCACGTATAAATATGCTGAAGTAATTTACTCTGAAGCTAAATTAACTAGATTAGATGAG
TGTATTATATACAAAAGGCATTAAATCAGATACATCTTAGACAAATTGTCACGGTCTACCAGAAAAGAAATTGCA
TTTGTTTTTGGGTCTTTCAGACTGACAAGATCGATCTGAAGTCTAAACAATTCTAAGAGGTATCATGTAGCAA

>lcl|HB386793 MON87701|cry1Ac|7899-11442
TGGACAACAACCCAAACATCAACGAATGCATTCCATACAACTGCTTGAGTAACCCAGAAGTTGAAGTACTTGGTG
GAGAACGCATTGAAACCGGTTACACTCCCATCGACATCTCCTTGTCCTTGACACAGTTTCTGCTCAGCGAGTTCG
TGCCAGGTGCTGGGTTCGTTCTCGGACTAGTTGACATCATCTGGGGTATCTTTGGTCCATCTCAATGGGATGCAT
TCCTGGTGCAAATTGAGCAGTTGATCAACCAGAGGATCGAAGAGTTCGCCAGGAACCAGGCCATCTCTAGGTTGG
AAGGATTGAGCAATCTCTACCAAATCTATGCAGAGAGCTTCAGAGAGTGGGAAGCCGATCCTACTAACCCAGCTC
TCCGCGAGGAAATGCGTATTCAATTCAACGACATGAACAGCGCCTTGACCACAGCTATCCCATTGTTCGCAGTCC
AGAACTACCAAGTTCCTCTCTTGTCCGTGTACGTTCAAGCAGCTAATCTTCACCTCAGCGTGCTTCGAGACGTTA
GCGTGTTTGGGCAAAGGTGGGGATTCGATGCTGCAACCATCAATAGCCGTTACAACGACCTTACTAGGCTGATTG
GAAACTACACCGACCACGCTGTTCGTTGGTACAACACTGGCTTGGAGCGTGTCTGGGGTCCTGATTCTAGAGATT
GGATTAGATACAACCAGTTCAGGAGAGAATTGACCCTCACAGTTTTGGACATTGTGTCTCTCTTCCCGAACTATG
ACTCCAGAACCTACCCTATCCGTACAGTGTCCCAACTTACCAGAGAAATCTATACTAACCCAGTTCTTGAGAACT
TCGACGGTAGCTTCCGTGGTTCTGCCCAAGGTATCGAAGGCTCCATCAGGAGCCCACACTTGATGGACATCTTGA
ACAGCATAACTATCTACACCGATGCTCACAGAGGAGAGTATTACTGGTCTGGACACCAGATCATGGCCTCTCCAG
TTGGATTCAGCGGGCCCGAGTTTACCTTTCCTCTCTATGGAACTATGGGAAACGCCGCTCCACAACAACGTATCG
TTGCTCAACTAGGTCAGGGTGTCTACAGAACCTTGTCTTCCACCTTGTACAGAAGACCCTTCAATATCGGTATCA
ACAACCAGCAACTTTCCGTTCTTGACGGAACAGAGTTCGCCTATGGAACCTCTTCTAACTTGCCATCCGCTGTTT
ACAGAAAGAGCGGAACCGTTGATTCCTTGGACGAAATCCCACCACAGAACAACAATGTGCCACCCAGGCAAGGAT
TCTCCCACAGGTTGAGCCACGTGTCCATGTTCCGTTCCGGATTCAGCAACAGTTCCGTGAGCATCATCAGAGCTC
CTATGTTCTCTTGGATACATCGTAGTGCTGAGTTCAACAACATCATCGCATCCGATAGTATTACTCAAATCCCTG
CAGTGAAGGGAAACTTTCTCTTCAACGGTTCTGTCATTTCAGGACCAGGATTCACTGGTGGAGACCTCGTTAGAC
TCAACAGCAGTGGAAATAACATTCAGAATAGAGGGTATATTGAAGTTCCAATTCACTTCCCATCCACATCTACCA
GATATAGAGTTCGTGTGAGGTATGCTTCTGTGACCCCTATTCACCTCAACGTTAATTGGGGTAATTCATCCATCT
TCTCCAATACAGTTCCAGCTACAGCTACCTCCTTGGATAATCTCCAATCCAGCGATTTCGGTTACTTTGAAAGTG
CCAATGCTTTTACATCTTCACTCGGTAACATCGTGGGTGTTAGAAACTTTAGTGGGACTGCAGGAGTGATTATCG
ACAGATTCGAGTTCATTCCAGTTACTGCAACACTCGAGGCTGAGTACAACCTTGAGAGAGCCCAGAAGGCTGTGA
ACGCCCTCTTTACCTCCACCAATCAGCTTGGCTTGAAAACTAACGTTACTGACTATCACATTGACCAAGTGTCCA
ACTTGGTCACCTACCTTAGCGATGAGTTCTGCCTCGACGAGAAGCGTGAACTCTCCGAGAAAGTTAAACACGCCA
AGCGTCTCAGCGACGAGAGGAATCTCTTGCAAGACTCCAACTTCAAAGACATCAACAGGCAGCCAGAACGTGGTT
GGGGTGGAAGCACCGGGATCACCATCCAAGGAGGCGACGATGTGTTCAAGGAGAACTACGTCACCCTCTCCGGAA
CTTTCGACGAGTGCTACCCTACCTACTTGTACCAGAAGATCGATGAGTCCAAACTCAAAGCCTTCACCAGGTATC
AACTTAGAGGCTACATCGAAGACAGCCAAGACCTTGAAATCTACTCGATCAGGTACAATGCCAAGCACGAGACCG
TGAATGTCCCAGGTACTGGTTCCCTCTGGCCACTTTCTGCCCAATCTCCCATTGGGAAGTGTGGAGAGCCTAACA

GATGCGCTCCACACCTTGAGTGGAATCCTGACTTGGACTGCTCCTGCAGGGATGGCGAGAAGTGTGCCCACCATT
CTCATCACTTCTCCTTGGACATCGATGTGGGATGTACTGACCTGAATGAGGACCTCGGAGTCTGGGTCATCTTCA
AGATCAAGACCCAAGACGGACACGCAAGACTTGGCAACCTTGAGTTTCTCGAAGAGAAACCATTGGTCGGTGAAG
CTCTCGCTCGTGTGAAGAGAGCAGAGAAGAAGTGGAGGGACAAACGTGAGAAACTCGAATGGGAAACTAACATCG
TTTACAAGGAGGCCAAAGAGTCCGTGGATGCTTTGTTCGTGAACTCCCAATATGATCAGTTGCAAGCCGACACCA
ACATCGCCATGATCCACGCCGCAGACAAACGTGTGCACAGCATTCGTGAGGCTTACTTGCCTGAGTTGTCCGTGA
TCCCTGGTGTGAACGCTGCCATCTTCGAGGAACTTGAGGGACGTATCTTTACCGCATTCTCCTTGTACGATGCCA
GAAACGTCATCAAGAACGGTGACTTCAACAATGGCCTCAGCTGCTGGAATGTGAAAGGTCATGTGGACGTGGAGG
AACAGAACAATCAGCGTTCCGTCCTGGTTGTGCCTGAGTGGGAAGCTGAAGTGTCCCAAGAGGTTAGAGTCTGTC
CAGGTAGAGGCTACATTCTCCGTGTGACCGCTTACAAGGAGGGATACGGTGAGGGTTGCGTGACCATCCACGAGA
TCGAGAACAACACCGACGAGCTTAAGTTCTCCAACTGCGTCGAGGAAGAAATCTATCCCAACAACACCGTTACTT
GCAACGACTACACTGTGAATCAGGAAGAGTACGGAGGTGCCTACACTAGCCGTAACAGAGGTTACAACGAAGCTC
CTTCCGTTCCTGCTGACTATGCCTCCGTGTACGAGGAGAAATCCTACACAGATGGCAGACGTGAGAACCCTTGCG
AGTTCAACAGAGGTTACAGGGACTACACACCACTTCCAGTTGGCTATGTTACCAAGGAGCTTGAGTACTTTCCTG
AGACCGACAAAGTGTGGATCGAGATCGGTGAAACCGAGGGAACCTTCATCGTGGACAGCGTGGAGCTTCTCTTGA
TGGAGGAATAATGAGATCC

Data S5. Element database , an un-redundant element database, containing only the longest of the element sequences.

```
>lcl|JQ406879 MON810|5'Maize|1-942
GAGACATCTTCGGAAATGCTGACAAAAGTGCTCTCAAAGCCGAAGCTTAAAAAATCTAAAAAGCCAAGCAAATGT
TGGTGCGCAAGAGCTGAAAATTGAGTAAGAAAGAGCAGATGGCAAGAAAGCGTGCCAAATGAGCTCGTGGTGCGC
TCTTATTTATACGCCTAGTGCGCTGAAAACTGGAAGGGCCCGCTTGTCAGTGACTGTTGCTATTCTAGCAAAGGA
AAGGTGTTTTTTCGGACCTTCGGCTTAGGGCCTTCGTCCATATCGCAATCTAAATTTATCATTCTAACAAATTAA
TATTACGAGGGGCTACTGTTGGTGGCCTTCGGCTTCTGAAGGTCCTCAAAAACATGATTTAACAAAGTTTCTGGA
GTATGATGCATGAACAGGTATCTTCGGACTTGAGTTAAAACCACAGTGTGAAGAAGCACAAAAGGAATACGAAGG
ATGTCGCGGAGCCGAAGCTGTGCGCAGAAGAGCTTCGAGATAATAGCAGAAAAGGAAACCGACTTAAAGATGAAA
AGGCTATTTAGACCTCGACGGATTACTATAGAGTTATTAGCAAATGTAGAGGGCATGGGTGTAATTTCATATGGG
CTGCGTCTCGTGCCTATAAATAGATGAACAGTGTTTCCGTACTGTTCGCGCTGACTTGGCATTTGCTTTTGCGCC
ACGCTTATACTTTTACCTTCTTTCAAGCCGAAGGTACATCTGTAATTTGATATCATTTCTATTCTTCCATGATAA
TAAAATAGAAATAAGTTGATTATAATATATAATTGTTTATGTTATCTCTTATACTTCATATGATTCCTTCTTCAT
TATTATATCTTGTGCTGATGAAGGTATGTCCTTCATAACCTTCGCCCGAAAATCATTATATCCCAAGGGAAATAA
TGCTTCGAAGGACGAAGGACTCTAACGTTTAACATCCTTTGC

>lcl|AY326434 MON810|hsp70|323-1134
GATCTACCGTCTTCGGTACGCGCTCACTCCGCCCTCTGCCTTTGTTACTGCCACGTTTCTCTGAATGCTCTCTTG
TGTGGTGATTGCTGAGAGTGGTTTAGCTGGATCTAGAATTACACTCTGAAATCGTGTTCTGCCTGTGCTGATTAC
TTGCCGTCCTTTGTAGCAGCAAAATATAGGGACATGGTAGTACGAAACGAAGATAGAACCTACACAGCAATACGA
GAAATGTGTAATTTGGTGCTTAGCGGTATTTATTTAAGCACATGTTGGTGTTATAGGGCACTTGGATTCAGAAGT
TTGCTGTTAATTTAGGCACAGGCTTCATACTACATGGGTCAATAGTATAGGGATTCATATTATAGGCGATACTAT
AATAATTTGTTCGTCTGCAGAGCTTATTATTTGCCAAAATTAGATATTCCTATTCTGTTTTTGTTTGTGTGCTGT
TAAATTGTTAACGCCTGAAGGAATAAATATAAATGACGAAATTTTGATGTTTATCTCTGCTCCTTTATTGTGACC
ATAAGTCAAGATCAGATGCACTTGTTTTAAATATTGTTGTCTGAAGAAATAAGTACTGACAGTATTTTGATGCAT
TGATCTGCTTGTTTGTTGTAACAAAATTTAAAAATAAAGAGTTTCCTTTTTGTTGCTCTCCTTACCTCCTGATGG
TATCTAGTATCTACCAACTGACACTATATTGCTTCTCTTTACATACGTATCTTGCTCGATGCCTTCTCCCTAGTG
TTGACCAGTGTTACTCACATAGTCTTTGCTCATTTCATTGTAATGCAGATACCAAGCGGCCA

>lcl|AY326434 MON810|cry1Ab|1135-3591
TGGACAACAACCCAAACATCAACGAGTGCATCCCGTACAACTGCCTCAGCAACCCTGAGGTCGAGGTGCTCGGCG
GTGAGCGCATCGAGACCGGTTACACCCCCATCGACATCTCCCTCTCCCTCACGCAGTTCCTGCTCAGCGAGTTCG
TGCCAGGCGCTGGCTTCGTCCTGGGCCTCGTGGACATCATCTGGGGCATCTTTGGCCCCTCCCAGTGGGACGCCT
TCCTGGTGCAAATCGAGCAGCTCATCAACCAGAGGATCGAGGAGTTCGCCAGGAACCAGGCCATCAGCCGCCTGG
AGGGCCTCAGCAACCTCTACCAAATCTACGCTGAGAGCTTCCGCGAGTGGGAGGCCGACCCCACTAACCCAGCTC
TCCGCGAGGAGATGCGCATCCAGTTCAACGACATGAACAGCGCCCTGACCACCGCCATCCCACTCTTCGCCGTCC
AGAACTACCAAGTCCCGCTCCTGTCCGTGTACGTCCAGGCCGCCAACCTGCACCTCAGCGTGCTGAGGGACGTCA
GCGTGTTTGGCCAGAGGTGGGGCTTCGACGCCGCCACCATCAACAGCCGCTACAACGACCTCACCAGGCTGATCG
GCAACTACACCGACCACGCTGTCCGCTGGTACAACACTGGCCTGGAGCGCGTCTGGGGCCCTGATTCTAGAGACT
GGATTCGCTACAACCAGTTCAGGCGCGAGCTGACCCTCACCGTCCTGGACATTGTGTCCCTCTTCCCGAACTACG
ACTCCCGCACCTACCCGATCCGCACCGTGTCCCAACTGACCCGCGAAATCTACACCAACCCCGTCCTGGAGAACT
TCGACGGTAGCTTCAGGGGCAGCGCCCAGGGCATCGAGGGCTCCATCAGGAGCCCACACCTGATGGACATCCTCA
ACAGCATCACTATCTACACCGATGCCCACCGCGGCGAGTACTACTGGTCCGGCCACCAGATCATGGCCTCCCCGG
TCGGCTTCAGCGGCCCCGAGTTTACCTTTCCTCTCTACGGCACGATGGGCAACGCCGCTCCACAACAACGCATCG
TCGCTCAGCTGGGCCAGGGCGTCTACCGCACCCTGAGCTCCACCCTGTACCGCAGGCCCTTCAACATCGGTATCA
ACAACCAGCAGCTGTCCGTCCTGGATGGCACTGAGTTCGCCTACGGCACCTCCTCCAACCTGCCCTCCGCTGTCT
ACCGCAAGAGCGGCACGGTGGATTCCCTGGACGAGATCCCACCACAGAACAACAATGTGCCCCCCAGGCAGGGTT
TTTCCCACAGGCTCAGCCACGTGTCCATGTTCCGCTCCGGCTTCAGCAACTCGTCCGTGAGCATCATCAGAGCTC
CTATGTTCTCCTGGATTCATCGCAGCGCGGAGTTCAACAATATCATTCCGTCCTCCCAAATCACCCAAATCCCCC
TCACCAAGTCCACCAACCTGGGCAGCGGCACCTCCGTGGTGAAGGGCCCAGGCTTCACGGGCGGCGACATCCTGC
GCAGGACCTCCCCGGGCCAGATCAGCACCCTCCGCGTCAACATCACCGCTCCCCTGTCCCAGAGGTACCGCGTCA
```

GGATTCGCTACGCTAGCACCACCAACCTGCAATTCCACACCTCCATCGACGGCAGGCCGATCAATCAGGGTAACT
TCTCCGCCACCATGTCCAGCGGCAGCAACCTCCAATCCGGCAGCTTCCGCACCGTGGGTTTCACCACCCCCTTCA
ACTTCTCCAACGGCTCCAGCGTTTTCACCCTGAGCGCCCACGTGTTCAATTCCGGCAATGAGGTGTACATTGACC
GCATTGAGTTCGTGCCAGCCGAGGTCACCTTCGAAGCCGAGTACGACCTGGAGAGAGCCCAGAAGGCTGTCAATG
AGCTCTTCACGTCCAGCAATCAGATCGGCCTGAAGACCGACGTCACTGACTACCACATCGACCAAGTCTCCAACC
TCGTGGAGTGCCTCTCCGATGAGTTCTGCCTCGACGAGAAGAAGGAGCTGTCCGAGAAGGTGAAGCATGCCAAGC
GTCTCAGCGACGAGAGGAATCTCCTCCAGGACCCCAATTTCCGCGGCATCAACAGGCAGCTCGACCGCGGCTGGC
GCGGCAGCACCGACATCACGATCCAGGGCGGCGACGATGTGTTCAAGGAGAACTACGTGACTCTCCTGGGCACTT
TCGACGAGTGCTACCCTACCTACTTGTACCAGAAGATCGATGAGTCCAAGCTCAAGGCTTACACTCGCTACCAGC
TCCGCGGCTACATCGAAGACAGCCAAGACCTCGAGATTTACCTGATCCGCTACAACGCCAAGCACGAGACCGTCA
ACGTGCCCGGTACTGGTTCCCTCTGGCCGCTGAGCGCCCCCAGCCCGATCGGCAAGTGTGCCCACCACAGCCACC
ACTTCTCCTTGGACATCGATGTGGGCTGCACCGACCTGAACGAGGACTTTCGGTAGC

>lcl|AY326434 MON810|3'Maize|3581-4180
CTTTCGGTAGCCTTCTTTCATTTCCGAATTTGCTTGCGAGCAGTCAGGTCCTTTTGATTCATCTGAGTTTGGCTT
TACAATAGCTTTTCCTTTTCCTTTGGCAGTACTAGTGCTTTCATCATGAGAATCCTTCTTAGATGTAAGACCACC
TGCAGCAGATGACTTTGATCTTGTTGTTGGGCGCCGACCAGATTGAGCCATTGCAGCTGTTAATGATGCACCAGC
CGTGGTGCCAGGAACCCCAGATTCAGAATTATTACCAGATGGAATTATAGGCTTCGATGCAACCTCACTGCGTTG
AACTCTAGGCCAAAGGAATTCTTCAACAGATGCAAGACTAGCAAATGGGTCGATAAGCACAATATTTGATGAATA
ATCCCGAAGTGATTTTTCGCCATGAGCTCGGGAAAGACGAAGCTTGAAGGGTTGAGCCAGAGCACTAAGACCTGA
AGTCAGACGAGACCCTCCAATACCAATCCTACTAGACTGGCTGAGCACAACAGGGAAACGTTCCAGCGAAGACAA
TGCACTTTGCAGTTTCTGAACCAACAGTGCCATGGGAGTTTCATCTCTCTCGACCAATCGAATTTCCGCGGGCC

>lcl|DI362404 MON89034|5'Maize|1-2071
ATATTTAAAAATGGAAGTAATACTATATTAAAATGATTCATGTGGAACTCCTGCGCTTCTTTTTGAAGTTTCAAA
AGGGAGCTTTCAGGGTCGCTTAGAGTTTGTTTGGTTGGAAATACAAGCGAAAAGAGAGCTAATGAGGGGGACATC
CATATTTTCTATGGTGTTTGAATAAGAGTCACGCGGGAATAAGATGAACACCGAAACAATTTTTTTGTAGCTACG
TGGTTCCAAAAAATCGAGTAGACGGTGTCGCTTCCACCTCATACTACTTCAACCTCAAACCACACATCCTTACGC
GCCCGCCGGTGTCTCCGTCGTCTCAAGTTCTCAACATACCTACACATGTAACCACTACCGGTCTTTGTCATGTTT
CGAAAGAAGATTACAGGTCCTCTAGAAGAGAGGACGCGGGGTGGCGAGAAAGCTGGGGAAGAAAAAGGCTAGTAC
ATATGATTGGTCTGTGAACCTGTGAGGTGGGTAGGTAGGTAGGTGGAGATTTTTGTTAACTGGTGTTGTTGACGG
ACTCGAACGGGGCCGGGCGTGTGGTGTGGCTAGCTGTGGTGGTTTGCTCGCCAGCCAGCCAGCCACACATCAGCG
AGCATGCAGAGCTTAAGCATGTATGTACGGATCGGCTTGCTTAGCGGTTAAGGGTGTGTTTGGTTTGGCTTTTGG
CTTTGGCTTTTGCCCCCTAAAAGCCAAAAGCCAACCAAGGGTGTATTTGGTTTGACTTTTGGCTTTTGGCTTTTG
TCCCCTAAAAGCCAAAAGCCAAACAAAGGGTTAGATCTAGGAAGCAGCTTTTTCTAAAAGCTGGCTTTCTCACAG
TGCAAATCTGAAAGCACCCCTGAACCTGCTTTTAGTGGCTTTTCGAATGGAACTGTGAAAACATATATCGAAGAA
CTTTTAACGACTTTTAGTGGTTTCCACCAAACAGTTTAGCTTTTTAACGGCTTACAGCCTACAACAGCTTTTTCC
ACAGCTCACAGCCCACAGCAACTTTTTTCACAGCCACAGCCCAACCAAACAGACCCCAAAGGGCTGAATCCAGGA
AGCAGCTTTTTCTAAAAGCCGACTTTCTCGTAGTGTAAAACTGAAAACACCCCTGGACCTGCTTTTAGTGGCTTT
TGGATGGAACTGTGAAAACATATATCGAAGAACTTTTAACGACTTTTAGTGGTTTCCACCAAACGATTTCTAGCT
TTTTAACAGCACACAGCCTACAACAGCTTTTTTCACAGCTCACAGCCCACAACAACTTTTTCTACAGCCACAACC
CAACCAAACGGACCCTAAGGCGGCCGAGCGAGCGCAAAGCGTCGTCAGCTTTGATTGCCATGCCATCTCCTGCTC
CACTTGTCTCTCTGGCCGTCGTCAGCCACCATCCAACAAGGCCGGTGCTACTGGCGGCTCCTAGGTAGACGACGA
CGACGACGACACCTCCACCGTTCGCCGCCGTCCACTCACCAATCAACACGGAACGCCCAAAACACACACACACGC
ACGCTGGGGAGGGAAAAAAAGGCAGAGACATATGCGTGCGTCGCTGCATTATTGTACGCGATCGAATGGCATCTC
TCTCACTCTCTCTCCTCCCTTTATTAATCTGGTACTGGCTAGCTGGTCCGGCGACACCGACGTGTCAGCTCCGTC
GCGCGCGTCCGTCCGTCCGCTGGAGCGGACACGGACCGCGGCGTCTGTCGATCGCCGGCTCGCCGCAGCGCAGCT
ACCTAGCACGCTCACGCATGCTACACTGCCTACACGCACACGGCCGGCCCAAAAGCGTTCCCTGCCGCCTGCCGG
CCGGCTTTTTTATTATTATTGGAACATGAGGCTATTTCTCCTCCCACACGGGCTACGACGTGAGCACGAGTACTG
GGATCCCCGGATCCGCCCTCTCTGTCCCTGCTGCTACTCCAGCCACTGAAATGTTGTCAGATGAAACAGCAGAGC
CGATCTCCGCACGGAAACCCATGCACGGCCATTCAAATTCAGGTGCCCACGTACGTCAGGGTGCTGCTGCTACTA
CTATCAAGCCAATAAAAGGATGGTAATGAGTATGATGGATCAGCAA

>lcl|DI362404 MON89034|LTa.Ihcbl|2678-2738
ACCATCTTCCACACACTCAAGCCACACTATTGGAGAACACACAGGGACAACACACCATAAG
>lcl|DI362404 MON89034|Os.Act|2755-3234
CGCCGCCGCCGGTAACCACCCCGCCCCTCTCCTCTTTCTTTCTCCGTTTTTTTTTCCGTCTCGGTCTCGATCTTT
GGCCTTGGTAGTTTGGGTGGGCGAGAGGCGGCTTCGTGCGCGCCCAGATCGGTGCGCGGGAGGGGCGGGATCTCG
CGGCTGGGGCTCTCGCCGGCGTGGATCCGGCCCGGATCTCGCGGGGAATGGGGCTCTCGGATGTAGATCTGCGAT
CCGCCGTTGTTGGGGGAGATGATGGGGGGTTTAAAATTTCCGCCGTGCTAAACAAGATCAGGAAGAGGGGAAAAG
GGCACTATGGTTTATATTTTTATATATTTCTGCTGCTTCGTCAGGCTTAGATGTGCTAGATCTTTCTTTCTTCTT
TTTGTGGGTAGAATTTGAATCCCTCAGCATTGTTCATCGGTAGTTTTTCTTTTCATGATTTGTGACAAATGCAGC
CTCGTGCGGAGCTTTTTTGTAGGTAGAAGT

>lcl|DI362404 MON89034|Cry1A.105|3244-6777
TGGACAACAACCCAAACATCAACGAGTGCATCCCGTACAACTGCCTCAGCAACCCTGAGGTCGAGGTGCTCGGCG
GTGAGCGCATCGAGACCGGTTACACCCCCATCGACATCTCCCTCTCCCTCACGCAGTTCCTGCTCAGCGAGTTCG
TGCCAGGCGCTGGCTTCGTCCTGGGCCTCGTGGACATCATCTGGGGCATCTTTGGCCCCTCCCAGTGGGACGCCT
TCCTGGTGCAAATCGAGCAGCTCATCAACCAGAGGATCGAGGAGTTCGCCAGGAACCAGGCCATCAGCCGCCTGG
AGGGCCTCAGCAACCTCTACCAAATCTACGCTGAGAGCTTCCGCGAGTGGGAGGCCGACCCCACTAACCCAGCTC
TCCGCGAGGAGATGCGCATCCAGTTCAACGACATGAACAGCGCCCTGACCACCGCCATCCCACTCTTCGCCGTCC
AGAACTACCAAGTCCCGCTCCTGTCCGTGTACGTCCAGGCCGCCAACCTGCACCTCAGCGTGCTGAGGGACGTCA
GCGTGTTTGGCCAGAGGTGGGGCTTCGACGCCGCCACCATCAACAGCCGCTACAACGACCTCACCAGGCTGATCG
GCAACTACACCGACCACGCTGTCCGCTGGTACAACACTGGCCGTCCTGGACATTGTGTCCCTCTTCCCGAACTAC
GACTCCCGCACCTACCCGATCCGCACCGTGTCCCAACTGACCCGCGAAATCTACACCAACCCCGTCCTGGAGAAC
TTCGACGGTAGCTTCAGGGGCAGCGCCCAGGGCATCGAGGGCTCCATCAGGAGCCCACACCTGATGGACATCCTC
AACAGCATCACTATCTACACCGATGCCCACCGCGGCGAGTACTACTGGTCCGGCCACCAGATCATGGCCTCCCCG
GTCGGCTTCAGCGGCCCCGAGTTTACCTTTCCTCTCTACGGCACGATGGGCAACGCCGCTCCACAACAACGCATC
GTCGCTCAGCTGGGCCAGGGCGTCTACCGCACCCTGAGCTCCACCCTGTACCGCAGGCCCTTCAACATCGGTATC
AACAACCAGCAGCTGTCCGTCCTGGATGGCACTGAGTTCGCCTACGGCACCTCCTCCAACCTGCCCTCCGCTGTC
TACCGCAAGAGCGGCACGGTGGATTCCCTGGACGAGATCCCACCACAGAACAACAATGTGCCCCCCAGGCAGGGT
TTTTCCCACAGGCTCAGCCACGTGTCCATGTTCCGCTCCGGCTTCAGCAACTCGTCCGTGAGCATCATCAGAGCT
CCTATGTTCTCTTGGATACACCGTAGTGCTGAGTTCAACAACATCATTGCATCCGACAGCATTACTCAAATACCC
TTGGTGAAAGCACATACACTTCAGTCAGGTACTACTGTTGTCAGAGGTCCAGGGTTTACAGGAGGAGACATTCTT
CGTCGCACAAGTGGAGGACCCTTTGCTTACACTATTGTTAACATCAATGGCCAATTGCCCCAAAGGTATCGTGCA
AGAATCCGCTATGCCTCTACTACAAATCTCAGGATCTACGTGACTGTTGCAGGTGAAAGGATCTTTGCTGGTCAG
TTCAACAAGACTATGGATACCGGTGACCCTTTGACATTCCAATCTTTTAGCTACGCAACTATCAACACAGCTTTT
ACATTCCCAATGAGCCAGAGTAGCTTCACAGTAGGTGCTGACACTTTCAGCTCAGGGAATGAAGTTTACATCGAC
AGGTTTGAATTGATTCCAGTTACTGCAACCCTCGAGGCTGAGTACAACCTTGAGAGAGCCCAGAAGGCTGTGAAC
GCCCTCTTTACCTCCACCAATCAGCTTGGCTTGAAAACTAACGTTACTGACTATCACATTGACCAAGTGTCCAAC
TTGGTCACCTACCTTAGCGATGAGTTCTGCCTCGACGAGAAGCGTGAACTCTCCGAGAAAGTTAAACACGCCAAG
CGTCTCAGCGACGAGAGGAATCTCTTGCAAGACTCCAACTTCAAAGACATCAACAGGCAGCCAGAACGTGGTTGG
GGTGGAAGCACCGGGATCACCATCCAAGGAGGCGACGATGTGTTCAAGGAGAACTACGTCACCCTCTCCGGAACT
TTCGACGAGTGCTACCCTACCTACTTGTACCAGAAGATCGATGAGTCCAAACTCAAAGCCTTCACCAGGTATCAA
CTTAGAGGCTACATCGAAGACAGCCAAGACCTTGAAATCTACTCGATCAGGTACAATGCCAAGCACGAGACCGTG
AATGTCCCAGGTACTGGTTCCCTCTGGCCACTTTCTGCCCAATCTCCCATTGGGAAGTGTGGAGAGCCTAACAGA
TGCGCTCCACACCTTGAGTGGAATCCTGACTTGGACTGCTCCTGCAGGGATGGCGAGAAGTGTGCCCACCATTCT
CATCACTTCTCCTTGGACATCGATGTGGGATGTACTGACCTGAATGAGGACCTCGGAGTCTGGGTCATCTTCAAG
ATCAAGACCCAAGACGGACACGCAAGACTTGGCAACCTTGAGTTTCTCGAAGAGAAACCATTGGTCGGTGAAGCT
CTCGCTCGTGTGAAGAGAGCAGAGAAGAAGTGGAGGGACAAACGTGAGAAACTCGAATGGGAAACTAACATCGTT
TACAAGGAGGCCAAAGAGTCCGTGGATGCTTTGTTCGTGAACTCCCAATATGATCAGTTGCAAGCCGACACCAAC
ATCGCCATGATCCACGCCGCAGACAAACGTGTGCACAGCATTCGTGAGGCTTACTTGCCTGAGTTGTCCGTGATC
CCTGGTGTGAACGCTGCCATCTTCGAGGAACTTGAGGGACGTATCTTTACCGCATTCTCCTTGTACGATGCCAGA
AACGTCATCAAGAACGGTGACTTCAACAATGGCCTCAGCTGCTGGAATGTGAAAGGTCATGTGGACGTGGAGGAA
CAGAACAATCAGCGTTCCGTCCTGGTTGTGCCTGAGTGGGAAGCTGAAGTGTCCCAAGAGGTTAGAGTCTGTCCA
GGTAGAGGCTACATTCTCCGTGTGACCGCTTACAAGGAGGGATACGGTGAGGGTTGCGTGACCATCCACGAGATC

GAGAACAACACCGACGAGCTTAAGTTCTCCAACTGCGTCGAGGAAGAAATCTATCCCAACAACACCGTTACTTGC
AACGACTACACTGTGAATCAGGAAGAGTACGGAGGTGCCTACACTAGCCGTAACAGAGGTTACAACGAAGCTCCT
TCCGTTCCTGCTGACTATGCCTCCGTGTACGAGGAGAAATCCTACACAGATGGCAGACGTGAGAACCCTTGCGAG
TTCAACAGAGGTTACAGGGACTACACACCACTTCCAGTTGGCTATGTTACCAAGGAGCTTGAGTACTTTCCTGAG
ACCGACAAAGTGTGGATCGAGATCGGTGAAACCGAGGGAACCTTCATCGTGGACAGCGTGGAGCTTCTCTTGATG
GAGGAATAATGAGATCTATCGATTCTAGAAGGCCTGAATTCTGCATGCGTTTGGACGTATGCTCATTCAGGTTGG
AGCCAATTT

>lcl|DI362404 MON89034|T-Hsp|6809-7018
GATGAGACATCTCTGTATTGTGTTTCTTTCCCCAGTGTTTTCTGTACTTGTGTAATCGGCTAATCGCCAACAGAT
TCGGCGATGAATAAATGAGAAATAAATTGTTCTGATTTTGAGTGCAAAAAAAAAGGAATTAGATCTGTGTGTGTT
TTTTGGATCCCCGGGGCGGCCGCGTTAACAAGCTTGAGCTCAGGATTTAGCAGCATTCCA

>lcl|DI362404 MON89034|P-FMV|7086-7649
AGGAACTCCCATCCTCAAAGGTTTGTAAGGAAGAATTCTCAGTCCAAAGCCTCAACAAGGTCAGGGTACAGAGTC
TCCAAACCATTAGCCAAAAGCTACAGGAGATCAATGAAGAATCTTCAATCAAAGTAAACTACTGTTCCAGCACAT
GCATCATGGTCAGTAAGTTTCAGAAAAAGACATCCACCGAAGACTTAAAGTTAGTGGGCATCTTTGAAAGTAATC
TTGTCAACATCGAGCAGCTGGCTTGTGGGGACCAGACAAAAAAGGAATGGTGCAGAATTGTTAGGCGCACCTACC
AAAAGCATCTTTGCCTTTATTGCAAAGATAAAGCAGATTCCTCTAGTACAAGTGGGGAACAAAATAACGTGGAAA
AGAGCTGTCCTGACAGCCCACTCACTAATGCGTATGACGAACGCAGTGACGACCACAAAAGAATTCCCTCTATAT
AAGAAGGCATTCATTCCCATTTGAAGGATCATCAGATACTCAACCAATCCTTCTAGGATCTACCGTCTTCGGTAC
GCGCTCACTCCGCCCTCTGCCTTTGTTACTGCCACGTTT

>lcl|DI362404 MON89034|Zm_CTP2|8492-8892
CTCCCCGTCGCCCGCCGCTCCTCCAGAAGCCTCGGCAACGTCAGCAACGGCGGAAGGATCCGGTGCATGCAGGTA
ACAAATGCATCCTAGCTAGTAGTTCTTTGCATTGCAGCAGCTGCAGCTAGCGAGTTAGTAATAGGAAGGGAACTG
ATGATCCATGCATGGACTGATGTGTGTTGCCCATCCCATCCCATTTCCCAACCCCAAACGAACCAAAACACACGT
ACTACGTGCAGGTGTGGCCGGCCTACGGCAACAAGAAGTTCGAGACGCTGTCGTACCTGCCGCCGCTGTCGACCG
GCGGGCGCATCCGCTGCATGCAGGCCATGGACAACTCCGTCCTGAACTCTGGTCGCACCACCATCTGCGACGCCT
ACAACGTCGCGGCGCATGATCCATTC

>lcl|DI362404 MON89034|Cry2Ab|8893-10745
AGCTTCCAGCACAAGAGCCTCGACACTGTTCAGAAGGAGTGGACGGAGTGGAAGAAGAACAACCACAGCCTGTAC
CTGGACCCCATCGTCGGCACGGTGGCCAGCTTCCTTCTCAAGAAGGTCGGCTCTCTCGTCGGGAAGCGCATCCTC
TCGGAACTCCGCAACCTGATCTTTCCATCTGGCTCCACCAACCTCATGCAAGACATCCTCAGGGAGACCGAGAAG
TTTCTCAACCAGCGCCTCAACACTGATACCCTTGCTCGCGTCAACGCTGAGCTGACGGGTCTGCAAGCAAACGTG
GAGGAGTTCAACCGCCAAGTGGACAACTTCCTCAACCCCAACCGCAATGCGGTGCCTCTGTCCATCACTTCTTCC
GTGAACACCATGCAACAACTGTTCCTCAACCGCTTGCCTCAGTTCCAGATGCAAGGCTACCAGCTGCTCCTGCTG
CCACTCTTTGCTCAGGCTGCCAACCTGCACCTCTCCTTCATTCGTGACGTGATCCTCAACGCTGACGAGTGGGGC
ATCTCTGCAGCCACGCTGAGGACCTACCGCGACTACCTGAAGAACTACACCAGGGACTACTCCAACTATTGCATC
AACACCTACCAGTCGGCCTTCAAGGGCCTCAATACGAGGCTTCACGACATGCTGGAGTTCAGGACCTACATGTTC
CTGAACGTGTTCGAGTACGTCAGCATCTGGTCGCTCTTCAAGTACCAGAGCCTGCTGGTGTCCAGCGGCGCCAAC
CTCTACGCCAGCGGCTCTGGTCCCCAACAAACTCAGAGCTTCACCAGCCAGGACTGGCCATTCCTGTATTCGTTG
TTCCAAGTCAACTCCAACTACGTCCTCAACGGCTTCTCTGGTGCTCGCCTCTCCAACACCTTCCCCAACATTGTT
GGCCTCCCCGGCTCCACCACAACTCATGCTCTGCTTGCTGCCAGAGTGAACTACTCCGGCGGCATCTCGAGCGGC
GACATTGGTGCATCGCCGTTCAACCAGAACTTCAACTGCTCCACCTTCCTGCCGCCGCTGCTCACCCCGTTCGTG
AGGTCCTGGCTCGACAGCGGCTCCGACCGCGAGGGCGTGGCCACCGTCACCAACTGGCAAACCGAGTCCTTCGAG
ACCACCCTTGGCCTCCGGAGCGGCGCCTTCACGGCGCGTGGAAATTCTAACTACTTCCCCGACTACTTCATCAGG
AACATCTCTGGTGTTCCTCTCGTCGTCCGCAACGAGGACCTCCGCCGTCCACTGCACTACAACGAGATCAGGAAC
ATCGCCTCTCCGTCCGGGACGCCCGGAGGTGCAAGGGCGTACATGGTGAGCGTCCATAACAGGAAGAACAACATC
CACGCTGTGCATGAGAACGGCTCCATGATCCACCTGGCGCCCAATGATTACACCGGCTTCACCATCTCTCCAATC
CACGCCACCCAAGTGAACAACCAGACACGCACCTTCATCTCCGAGAAGTTCGGCAACCAGGGCGACTCCCTGAGG
TTCGAGCAGAACAACACCACCGCCAGGTACACCCTGCGCGGCAACGGCAACAGCTACAACCTGTACCTGCGCGTC

AGCTCCATTGGCAACTCCACCATCAGGGTCACCATCAACGGGAGGGTGTACACAGCCACCAATGTGAACACGACG
ACCAACAATGATGGCGTCAACGACAACGGCGCCCGCTTCAGCGACATCAACATTGGCAACGTGGTGGCCAGCAGC
AACTCCGACGTCCCGCTGGACATCAACGTGACCCTGAACTCTGGCACCCAGTTCGACCTCATGAACATCATGCTG
GTGCCAACTAACATCTCGCCGCTGTACTGATAGGAGCTCTGATCCCCATGGGA

>lcl|DI362404 MON89034|3'Maize|11388-12208
AAAGTTTCTTCATGGCACATCTAGCTTTTGCCTAATACCATGTAGGGCTACATCTTAAAAATCTATACTACTATA
TTAAAGCTGCAGGGGTAGCCTGTCTCCACCTGGTTCTGCCTCGAGCCAATCTAAACCGTCCATCTATATCCATCA
AATCAGCACCGTCCGGTCCGTGCGCACCTCCTCTCCCGCTATTCAGTTGCATACTTGCAGCAGGTTCTCCCTCCT
CACCATTTCCTCTGCCTCCTCTCTCGCTCACTGGTCAGATTCATCCTGCCTCTCCCGCATGCGCTCCCTCCCCAT
GCCCCGTCTCGCACTATCGCCACACCTCACCGCGGGGAGACGAAGACGGTGGACGCATCCTCACCTCCTCCGCTA
GTTGTCGCTCTTCCATCCTCTTCAACAACTTCTACATAGGGAGAGGCGGTTCGGCGTCCCGACGCCGCCGCTTCT
CCCCTCCCCATGGAGGACGAGAACATCGACCTCGGCGGCGGGGGCGATGCCTCCGCTCTGCATAGAGGAGGGTTG
TAGTGGCAAGCAGCAATGCCAACACCGAGGCGGGCCAAGACTAGGCAACAATAGGACGGCACGCCCGGTTGTCAG
CGAGGTGGCGGCATCGTGTGCCGCTACCGAACAACATCTCCGGCGCTGGAGTCGGTGAGTTACTGCGCCACCCGG
ACGCCCTCAATGCACTGATATCTACCCGGTCTCCATCGCCGCCCTTCCTCCCTTCCCTCTCCCTGTGCCTCCCTC
TCTTGCCCTCTCCCTTCCAACTGCTCCCGCCCCAGCCCTAGCCCAACCACCTCCCGCGCAGGGTCACCAA

>lcl|HV702026 MON88017|5'Maize|1-103
ACCCGATCAGAGCGCTAAGCAGCAGAATCGTGTGACAACGCTAGCAGCTCTCCTCCAACACATCATCGACAAGCA
CCTTTTTTGCCGGAGTATGACGGTGACG

>lcl|HV702026 MON88017|P-ract|409-1814
GCTTACTCGAGGTCATTCATATGCTTGAGAAGAGAGTCGGGATAGTCCAAAATAAAACAAAGGTAAGATTACCTG
GTCAAAAGTGAAAACATCAGTTAAAAGGTGGTATAAAGTAAAATATCGGTAATAAAAGGTGGCCCAAAGTGAAAT
TTACTCTTTTCTACTATTATAAAAATTGAGGATGTTTTTGTCGGTACTTTGATACGTCATTTTTGTATGAATTGG
TTTTTAAGTTTATTCGCTTTTGGAAATGCATATCTGTATTTGAGTCGGGTTTTAAGTTCGTTTGCTTTTGTAAAT
ACAGAGGGATTTGTATAAGAAATATCTTTAGAAAAACCCATATGCTAATTTGACATAATTTTTGAGAAAAATATA
TATTCAGGCGAATTCTCACAATGAACAATAATAAGATTAAAATAGCTTTCCCCCGTTGCAGCGCATGGGTATTTT
TTCTAGTAAAAATAAAAGATAAACTTAGACTCAAAACATTTACAAAAACAACCCCTAAAGTTCCTAAAGCCCAAA
GTGCTATCCACGATCCATAGCAAGCCCAGCCCAACCCAACCCAACCCAACCCACCCCAGTCCAGCCAACTGGACA
ATAGTCTCCACACCCCCCCACTATCACCGTGAGTTGTCCGCACGCACCGCACGTCTCGCAGCCAAAAAAAAAAAG
AAAGAAAAAAAAGAAAAAGAAAAAACAGCAGGTGGGTCCGGGTCGTGGGGGCCGGAAACGCGAGGAGGATCGCGA
GCCAGCGACGAGGCCGGCCCTCCCTCCGCTTCCAAAGAAACGCCCCCCATCGCCACTATATACATACCCCCCCCT
CTCCTCCCATCCCCCCAACCCTACCACCACCACCACCACCACCTCCACCTCCTCCCCCCTCGCTGCCGGACGACG
AGCTCCTCCCCCCTCCCCCTCCGCCGCCGCCGCGCCGGTAACCACCCCGCCCCTCTCCTCTTTCTTTCTCCGTTT
TTTTTTCCGTCTCGGTCTCGATCTTTGGCCTTGGTAGTTTGGGTGGGCGAGAGGCGGCTTCGTGCGCGCCCAGAT
CGGTGCGCGGGAGGGGCGGGATCTCGCGGCTGGGGCTCTCGCCGGCGTGGATCCGGCCCGGATCTCGCGGGGAAT
GGGGCTCTCGGATGTAGATCTGCGATCCGCCGTTGTTGGGGGAGATGATGGGGGGTTTAAAATTTCCGCCGTGCT
AAACAAGATCAGGAAGAGGGGAAAAGGGCACTATGGTTTATATTTTTATATATTTCTGCTGCTTCGTCAGGCTTA
GATGTGCTAGATCTTTCTTTCTTCTTTTTGTGGGTAGAATTTGAATCCCTCAGCATTGTTCATCGGTAGTTTTTC
TTTTCATGATTTGTGACAAATGCAGCCTCGTGCGGAGCTTTTTTGTAGGTAGAAGT

>lcl|HV702026 MON88017|At_CTP2|1815-2048
GATCAACCATGGCGCAAGTTAGCAGAATCTGCAATGGTGTGCAGAACCCATCTCTTATCTCCAATCTCTCGAAAT
CCAGTCAACGCAAATCTCCCTTATCGGTTTCTCTGAAGACGCAGCAGCATCCACGAGCTTATCCGATTTCGTCGT
CGTGGGGATTGAAGAAGAGTGGGATGACGTTAATTGGCTCTGAGCTTCGTCCTCTTAAGGTCATGTCTTCTGTTT
CCACGGCGT

>lcl|HV702026 MON88017|cp4_epsps|2049-3420
GCATGCTTCACGGTGCAAGCAGCCGGCCCGCAACCGCCCGCAAATCCTCTGGCCTTTCCGGAACCGTCCGCATTC
CCGGCGACAAGTCGATCTCCCACCGGTCCTTCATGTTCGGCGGTCTCGCGAGCGGTGAAACGCGCATCACCGGCC
TTCTGGAAGGCGAGGACGTCATCAATACGGGCAAGGCCATGCAGGCGATGGGCGCCCGCATCCGTAAGGAAGGCG

ACACCTGGATCATCGATGGCGTCGGCAATGGCGGCCTCCTGGCGCCTGAGGCGCCGCTCGATTTCGGCAATGCCG
CCACGGGCTGCCGCCTGACGATGGGCCTCGTCGGGGTCTACGATTTCGACAGCACCTTCATCGGCGACGCCTCGC
TCACAAAGCGCCCGATGGGCCGCGTGTTGAACCCGCTGCGCGAAATGGGCGTGCAGGTGAAATCGGAAGACGGTG
ACCGTCTTCCCGTTACCTTGCGCGGGCCGAAGACGCCGACGCCGATCACCTACCGCGTGCCGATGGCCTCCGCAC
AGGTGAAGTCCGCCGTGCTGCTCGCCGGCCTCAACACGCCCGGCATCACGACGGTCATCGAGCCGATCATGACGC
GCGATCATACGGAAAAGATGCTGCAGGGCTTTGGCGCCAACCTTACCGTCGAGACGGATGCGGACGGCGTGCGCA
CCATCCGCCTGGAAGGCCGCGGCAAGCTCACCGGCCAAGTCATCGACGTGCCGGGCGACCCGTCCTCGACGGCCT
TCCCGCTGGTTGCGGCCCTGCTTGTTCCGGGCTCCGACGTCACCATCCTCAACGTGCTGATGAACCCCACCCGCA
CCGGCCTCATCCTGACGCTGCAGGAAATGGGCGCCGACATCGAAGTCATCAACCCGCGCCTTGCCGGCGGCGAAG
ACGTGGCGGACCTGCGCGTTCGCTCCTCCACGCTGAAGGGCGTCACGGTGCCGGAAGACCGCGCGCCTTCGATGA
TCGACGAATATCCGATTCTCGCTGTCGCCGCCGCCTTCGCGGAAGGGGCGACCGTGATGAACGGTCTGGAAGAAC
TCCGCGTCAAGGAAAGCGACCGCCTCTCGGCCGTCGCCAATGGCCTCAAGCTCAATGGCGTGGATTGCGATGAGG
GCGAGACGTCGCTCGTCGTGCGTGGCCGCCCTGACGGCAAGGGGCTCGGCAACGCCTCGGGCGCCGCCGTCGCCA
CCCATCTCGATCACCGCATCGCCATGAGCTTCCTCGTCATGGGCCTCGTGTCGGAAAACCCTGTCACGGTGGACG
ATGCCACGATGATCGCCACGAGCTTCCCGGAGTTCATGGACCTGATGGCCGGGCTGGGCGCGAAGATCGAACTCT
CCGATACGAAGGCTGCCTGATG

>lcl|HV702026 MON88017|NOS|3421-3694
AGCTCGAATTCCCGATCGTTCAAACATTTGGCAATAAAGTTTCTTAAGATTGAATCCTGTTGCCGGTCTTGCGAT
GATTATCATATAATTTCTGTTGAATTACGTTAAGCATGTAATAATTAACATGTAATGCATGACGTTATTTATGAG
ATGGGTTTTTATGATTAGAGTCCCGCAATTATACATTTAATACGCGATAGAAAACAAAATATAGCGCGCAAACTA
GGATAAATTATCGCGCGCGGTGTCATCTATGTTACTAGATCGGGGATTT

>lcl|HV702026 MON88017|P-35S|3695-4361
GCGGCCGCGTTAACAAGCTTCTGCAGGTCCGATTGAGACTTTTCAACAAAGGGTAATATCCGGAAACCTCCTCGG
ATTCCATTGCCCAGCTATCTGTCACTTTATTGTGAAGATAGTGGAAAAGGAAGGTGGCTCCTACAAATGCCATCA
TTGCGATAAAGGAAAGGCCATCGTTGAAGATGCCTCTGCCGACAGTGGTCCCAAAGATGGACCCCCACCCACGAG
GAGCATCGTGGAAAAAGAAGACGTTCCAACCACGTCTTCAAAGCAAGTGGATTGATGTGATGGTCCGATTGAGAC
TTTTCAACAAAGGGTAATATCCGGAAACCTCCTCGGATTCCATTGCCCAGCTATCTGTCACTTTATTGTGAAGAT
AGTGGAAAAGGAAGGTGGCTCCTACAAATGCCATCATTGCGATAAAGGAAAGGCCATCGTTGAAGATGCCTCTGC
CGACAGTGGTCCCAAAGATGGACCCCCACCCACGAGGAGCATCGTGGAAAAAGAAGACGTTCCAACCACGTCTTC
AAAGCAAGTGGATTGATGTGATATCTCCACTGACGTAAGGGATGACGCACAATCCCACTATCCTTCGCAAGACCC
TTCCTCTATATAAGGAAGTTCATTTCATTTGGAGAGGACACGCTGACAAGCTGACTCTAGCAGATCC

>lcl|HV702026 MON88017|wtCAB_leader|4362-4440
TCTAGAACCATCTTCCACACACTCAAGCCACACTATTGGAGAACACACAGGGACAACACACCATAAGATCCAAGG
GAGG

>lcl|HV702026 MON88017|ract_intron|4441-4924
CCTCCGCCGCCGCCGGTAACCACCCCGCCCCTCTCCTCTTTCTTTCTCCGTTTTTTTTTCCGTCTCGGTCTCGAT
CTTTGGCCTTGGTAGTTTGGGTGGGCGAGAGGCGGCTTCGTGCGCGCCCAGATCGGTGCGCGGGAGGGGCGGGAT
CTCGCGGCTGGGGCTCTCGCCGGCGTGGATCCGGCCCGGATCTCGCGGGGAATGGGGCTCTCGGATGTAGATCTG
CGATCCGCCGTTGTTGGGGGAGATGATGGGGGGTTTAAAATTTCCGCCGTGCTAAACAAGATCAGGAAGAGGGGA
AAAGGGCACTATGGTTTATATTTTTATATATTTCTGCTGCTTCGTCAGGCTTAGATGTGCTAGATCTTTCTTTCT
TCTTTTTGTGGGTAGAATTTGAATCCCTCAGCATTGTTCATCGGTAGTTTTTCTTTTCATGATTTGTGACAAATG
CAGCCTCGTGCGGAGCTTTTTTGTAGGTAGAAGT

>lcl|HV702026 MON88017|cry3Bb1|4925-6914
GATCAACCATGGCCAACCCCAACAATCGCTCCGAGCACGACACGATCAAGGTCACCCCCAACTCCGAGCTCCAGA
CCAACCACAACCAGTACCCGCTGGCCGACAACCCCAACTCCACCCTGGAAGAGCTGAACTACAAGGAGTTCCTGC
GCATGACCGAGGACTCCTCCACGGAGGTCCTGGACAACTCCACCGTCAAGGACGCCGTCGGGACCGGCATCTCCG
TCGTTGGGCAGATCCTGGGCGTCGTTGGCGTCCCCTTCGCAGGTGCTCTCACCTCCTTCTACCAGTCCTTCCTGA
ACACCATCTGGCCCTCCGACGCCGACCCCTGGAAGGCCTTCATGGCCCAAGTCGAAGTCCTGATCGACAAGAAGA

```
TCGAGGAGTACGCCAAGTCCAAGGCCCTGGCCGAGCTGCAAGGCCTGCAAAACAACTTCGAGGACTACGTCAACG
CGCTGAACTCCTGGAAGAAGACGCCTCTGTCCCTGCGCTCCAAGCGCTCCCAGGACCGCATCCGCGAGCTGTTCT
CCCAGGCCGAGTCCCACTTCCGCAACTCCATGCCGTCCTTCGCCGTCTCCAAGTTCGAGGTCCTGTTCCTGCCCA
CCTACGCCCAGGCTGCCAACACCCACCTCCTGTTGCTGAAGGACGCCCAGGTCTTCGGCGAGGAATGGGGCTACT
CCTCGGAGGACGTCGCCGAGTTCTACCGTCGCCAGCTGAAGCTGACCCAACAGTACACCGACCACTGCGTCAACT
GGTACAACGTCGGCCTGAACGGCCTGAGGGGCTCCACCTACGACGCATGGGTCAAGTTCAACCGCTTCCGCAGGG
AGATGACCCTGACCGTCCTGGACCTGATCGTCCTGTTCCCCTTCTACGACATCCGCCTGTACTCCAAGGGCGTCA
AGACCGAGCTGACCCGCGACATCTTCACGGACCCCATCTTCCTGCTCACGACCCTCCAGAAGTACGGTCCCACCT
TCCTGTCCATCGAGAACTCCATCCGCAAGCCCCACCTGTTCGACTACCTCCAGGGCATCGAGTTCCACACGCGCC
TGAGGCCAGGCTACTTCGGCAAGGACTCCTTCAACTACTGGTCCGGCAACTACGTCGAGACCAGGCCCTCCATCG
GCTCCTCGAAGACGATCACCTCCCCTTTCTACGGCGACAAGTCCACCGAGCCCGTCCAGAAGCTGTCCTTCGACG
GCCAGAAGGTCTACCGCACCATCGCCAACACCGACGTCGCGGCTTGGCCGAACGGCAAGGTCTACCTGGGCGTCA
CGAAGGTCGACTTCTCCCAGTACGATGACCAGAAGAACGAGACCTCCACCCAGACCTACGACTCCAAGCGCAACA
ATGGCCACGTCTCCGCCCAGGACTCCATCGACCAGCTGCCGCCTGAGACCACTGACGAGCCCCTGGAGAAGGCCT
ACTCCCACCAGCTGAACTACGCGGAGTGCTTCCTGATGCAAGACCGCAGGGGCACCATCCCCTTCTTCACCTGGA
CCCACCGCTCCGTCGACTTCTTCAACACCATCGACGCCGAGAAGATCACCCAGCTGCCCGTGGTCAAGGCCTACG
CCCTGTCCTCGGGTGCCTCCATCATTGAGGGTCCAGGCTTCACCGGTGGCAACCTGCTGTTCCTGAAGGAGTCCT
CGAACTCCATCGCCAAGTTCAAGGTCACCCTGAACTCCGCTGCCTTGCTGCAACGCTACCGCGTCCGCATCCGCT
ACGCCTCCACCACGAACCTGCGCCTGTTCGTCCAGAACTCCAACAATGACTTCCTGGTCATCTACATCAACAAGA
CCATGAACAAGGACGATGACCTGACCTACCAGACCTTCGACCTCGCCACCACGAACTCCAACATGGGCTTCTCGG
GCGACAAGAATGAACTGATCATTGGTGCTGAGTCCTTCGTCTCCAACGAGAAGATCTACATCGACAAGATCGAGT
TCATCCCCGTCCAGCTGTGATAGGAACTCTGATTGAATTC

>lcl|HV702026 MON88017|tahsp17|6915-7124
TGCATGCGTTTGGACGTATGCTCATTCAGGTTGGAGCCAATTTGGTTGATGTGTGTGCGAGTTCTTGCGAGTCTG
ATGAGACATCTCTGTATTGTGTTTCTTTCCCCAGTGTTTTCTGTACTTGTGTAATCGGCTAATCGCCAACAGATT
CGGCGATGAATAAATGAGAAATAAATTGTTCTGATTTTGAGTGCAAAAAAAAAGGAATTA

>lcl|HV702026 MON88017|3'Maize|7230-7450
GAGTCGGGTTTGGATGGTCAACTCCGGCATACTGCCGAAAACAAACCAATCCGTCACCGTCAAGGCCCCGCACCG
CTGGCCGCACGCAGGAAAAATAAGTTGCGACCGCGAGCGGGCGAATCAGAAAGGGCGTCCGGCCTTGGTCAGACA
CGACAGCGACGCGGAAAGGCTGCGCCCGCGGTGCCATCTACAAGGGTCCACGTCCATCCAAAAAGAGCGG

>lcl|EA135633 MON15985|5'Cotton|1-361
TTGTCATTGGTGCAGACAGGTTCGGGGAGTATGGCCTATGGGTGGTGGTAGAGAGAAAGTAACAGCGAAATCTTA
GGGTGGTCCGAGAAATGAGATTGTTAAGTCCTGATTTAATGTGCTGAGTTCAGTTGATGGAGAGGAAAATGGCAA
AGATTTGAGGGGAGAGGATTTTGTGGATCCTAACATTAAACAGAATCGTTCTACTAAAAGGGGTAAGGGTAGTGG
TGGAAGAGATATTTTTAAAAAAGCTAAGGTAAAATCTGGGCAAAGTTTTAACGTGGCCCAGGGGAATAAAAAAGG
GACTATAGGGCCTAGTGGTAGTCAACAGGCCGGGCGGGAAAGTATTCATTTGAAATGGGTT

>lcl|EA135633 MON15985|Chloroplast|362-750
TATTCGTATTAACAGCTACTCTAGGAGGAATGTTTTTATGCGGTGCTAACGATTTAATAACTATCTTTGTAGCTC
CAGAATGTTTCAGTTTATGCTCCTACCTATTATCTGGATATACCAAGAAAGATGTACGGTCTAATGAGGCTACTA
CGAAATATTTACTCATGGGTGGGGCAAGCTCTTCTATTCTGGTTCATGGTTTCTCTTGGCTATATGGTTCATCCG
GGGGAGAGATCGAGCTTCAAGAAATAGTGAATGGTCTTATCAATACACAAATGTATAACTCCCCAGGAATTTCAA
TTGCGCTTATATTCATCACTGTAGGAATTGGGTTCAAGCTTTCCCCAGCCCCTTCTCATCAATGGACTCCTGACG
TATACGAAGGAGTT

>lcl|EA135633 MON15985|Cotton(5')|751-1885
CTTTTAGGCTTTTGCATTTTGTTGAAAATTCTTATTTTCACAGGACTTTGCTTATCATAATTATTGACAGCCAAC
TGGTGAAAATGGTGCCACGAGAGCTCCCATCAGCATTGATTTGGCTAGGGATAGTTCTTTAAGTGCTAAGTCAAT
CATCTTGCAAATTGACAATAAATCTCAACAGGTTTCAGCAAGTAAGGCCTCCTTCATTATTTTGGTTGCTTCTTT
TTCATTAATGATGTTGCTTGTTTGCACCTGGAAAACATGCACCGTTCTTCATCATGCACCTTGGATATGGTCTTG
```

AGTGCTCTCTGACTGCCCGTCATCTTTCGAATTGGTGTCTGATTGAGTGCACCCGCCTAAGTTCAAGATGCCGGA
GTTTGAATAGTATAACGGGACTAGTTGTCTTGAGGCTCATATTACTATGTTCTGTCGATATAATCATCCATGGGC
TTCGCATCCACCATTGCAAGGCCCAACCACCTAGCACTGTGATGGGTCCAAACGCAAAGGTGATCCCTTTAGGCC
AAATGGATGGAGACGCTATAAGATTGGTCACCGCAAGAAAAGTGTGGATCGATAGGCCCTGTAGTTGATGATGGG
GATGATCACGATGACCCTGCCTATCCTCCAAGTTTCACCCCAACTAACATCCAAGTGCAACCAGAGGTGTACCCG
CAGAGGGCACCTATTACCATTAGATCGACTGCAAGTGTAGGTAATGTTGGACAGGGTAGATCAGGGTGTTCACGG
TGTGGTAGACTTTACTATGGTCCTTTTCGGGCAGGCGAAAATGTTTGTTATAAATGCGGCACTCCAGATCATTTT
GTACGAGAATGTCCAGAGGTGGCTAATCGAGAGAATCGAATCACTTACGAAAAAAGATTATTGTTACTAAGTTTT
CATGTTTTATGCCATGCGTAGCCATGTTTTTAATAAAGGGTTGAAGACTTGAATGACAAGTTAGCGTAAAGCATT
TTTTTTCTTTCATGTTGGCACATTTGATGCACTATGTCTTCGTCTATTTTTCAAACATACTGTTGGAAGAATTAT
GATTCACGTGTTGTTTAAGATCAAAAAGTGCATGCCTAACTAATACTTATCAGAAACAAATAATGCAATGAGTCA
TATCTCTATA

>lcl|U12639 pBI101|UidA|2551-4359
TGTTACGTCCTGTAGAAACCCCAACCCGTGAAATCAAAAAACTCGACGGCCTGTGGGCATTCAGTCTGGATCGCG
AAAACTGTGGAATTGATCAGCGTTGGTGGGAAAGCGCGTTACAAGAAAGCCGGGCAATTGCTGTGCCAGGCAGTT
TTAACGATCAGTTCGCCGATGCAGATATTCGTAATTATGCGGGCAACGTCTGGTATCAGCGCGAAGTCTTTATAC
CGAAAGGTTGGGCAGGCCAGCGTATCGTGCTGCGTTTCGATGCGGTCACTCATTACGGCAAAGTGTGGGTCAATA
ATCAGGAAGTGATGGAGCATCAGGGCGGCTATACGCCATTTGAAGCCGATGTCACGCCGTATGTTATTGCCGGGA
AAAGTGTACGTATCACCGTTTGTGTGAACAACGAACTGAACTGGCAGACTATCCCGCCGGGAATGGTGATTACCG
ACGAAAACGGCAAGAAAAAGCAGTCTTACTTCCATGATTTCTTTAACTATGCCGGAATCCATCGCAGCGTAATGC
TCTACACCACGCCGAACACCTGGGTGGACGATATCACCGTGGTGACGCATGTCGCGCAAGACTGTAACCACGCGT
CTGTTGACTGGCAGGTGGTGGCCAATGGTGATGTCAGCGTTGAACTGCGTGATGCGGATCAACAGGTGGTTGCAA
CTGGACAAGGCACTAGCGGGACTTTGCAAGTGGTGAATCCGCACCTCTGGCAACCGGGTGAAGGTTATCTCTATG
AACTGTGCGTCACAGCCAAAAGCCAGACAGAGTGTGATATCTACCCGCTTCGCGTCGGCATCCGGTCAGTGGCAG
TGAAGGGCCAACAGTTCCTGATTAACCACAAACCGTTCTACTTTACTGGCTTTGGTCGTCATGAAGATGCGGACT
TACGTGGCAAAGGATTCGATAACGTGCTGATGGTGCACGACCACGCATTAATGGACTGGATTGGGGCCAACTCCT
ACCGTACCTCGCATTACCCTTACGCTGAAGAGATGCTCGACTGGGCAGATGAACATGGCATCGTGGTGATTGATG
AAACTGCTGCTGTCGGCTTTAACCTCTCTTTAGGCATTGGTTTCGAAGCGGGCAACAAGCCGAAAGAACTGTACA
GCGAAGAGGCAGTCAACGGGGAAACTCAGCAAGCGCACTTACAGGCGATTAAAGAGCTGATAGCGCGTGACAAAA
ACCACCCAAGCGTGGTGATGTGGAGTATTGCCAACGAACCGGATACCCGTCCGCAAGTGCACGGGAATATTTCGC
CACTGGCGGAAGCAACGCGTAAACTCGACCCGACGCGTCCGATCACCTGCGTCAATGTAATGTTCTGCGACGCTC
ACACCGATACCATCAGCGATCTCTTTGATGTGCTGTGCCTGAACCGTTATTACGGATGGTATGTCCAAAGCGGCG
ATTTGGAAACGGCAGAGAAGGTACTGGAAAAAGAACTTCTGGCCTGGCAGGAGAAACTGCATCAGCCGATTATCA
TCACCGAATACGGCGTGGATACGTTAGCCGGGCTGCACTCAATGTACACCGACATGTGGAGTGAAGAGTATCAGT
GTGCATGGCTGGATATGTATCACCGCGTCTTTGATCGCGTCAGCGCCGTCGTCGGTGAACAGGTATGGAATTTCG
CCGATTTTGCGACCTCGCAAGGCATATTGCGCGTTGGCGGTAACAAGAAAGGGATCTTCACTCGCGACCGCAAAC
CGAAGTCGGCGGCTTTTCTGCTGCAAAAACGCTGGACTGGCATGAACTTCGGTGAAAAACCGCAGCAGGGAGGCA
AACAATGAA

>lcl|EA135634 MON15985|3'Cotton|350-1160
GCACTGAAATCCCATCCATTTAGCAACCTTAAAAGTAATAAACTTAAACTAAGTGTACTCCTTATCAAAACATAA
ACCAAGAGATGAGGATAACAGCGTTCGGCCAACCCCAATACCACCTATAATCAAAGCATGCGATGTCACGAACGT
TTTCCTGACGGCAATCGACACAAAGAAGAGATTAAAAAGGGAGACCACTATAGAAAGAAGAAAGAGAATCGGCAC
CACTGTGAAACTACATTTGATAACCGTATTAGCAAGGCTCCACCATTGATAAAAACTCGACAACCAAGAGGAACT
CACTTACCTTGATCAAAGTAGGGTTGTTGGTACTAAGGCTCAAAGCAAGGAAAATGTCTAAATGAGTGAGTTGGG
CTTTTTTAATTTAATTAAACTGAAAAATAAGAAAGGATGTTGCCAATTTTGTGTGGCAAGGACGGACAAGAGGTT
TCGACCCAACCTAGAGAAGACATTAAAACTGCAGAACTGGGCTCGCTTGTTCAGGATCGGGCTTTAAAGGAGAAT
GGTTTGATGGACCAGAATAGAGACAAACCAATACACTCAGATTTTGATCTTCCTTATTCTCCCTCAACTATTGAT
TTTGTTTTGGGTAATGATGGTGTAGCAGGGGATCGGGTTTTAGGTGTTTTTCAAGGCATGACTGTTACTCATTTT
AATCCGACTTGCGATGACCATAATGAGATGGAGGCTGTACTGAAGGATGAAGTATTGGATCCAAATAGGCATTCG
ACATGTTTGATAAAACTCTTAGTTACAAAATTCTTAGTAAACGAATCCATAAGGTTATCTT

>lcl|AX600169 MON15985|OriV|1-378
CCCTCAAATGTCAATAGGTGCGCCCCTCATCTGTCAGCACTCTGCCCCTCAAGTGTCAAGGATCGCGCCCCTCAT
CTGTCAGTAGTCGCGCCCCTCAAGTGTCAATACCGCAGGGCACTTATCCCCAGGCTTGTCCACATCATCTGTGGG
AAACTCGCGTAAAATCAGGCGTTTTCGCCGATTTGCGAGGCTGGCCAGCTCCACGTCGCCGGCCGAAATCGAGCC
TGCCCCTCATCTGTCAACGCCGCGCCGGGTGAGTCGGCCCCTCAAGTGTCAACGTCCGCCCCTCATCTGTCAGTG
AGGGCCAAGTTTTCCGCGAGGTATCCACAACGCCGGCGGCCGCGGTGTCTCGCACACGGCTTCGACGGCGTTTCT
GGT

>lcl|AM887683 pICH14313 MON351|NPTII|3861-4658
TGGGGATTGAACAAGATGGATTGCACGCAGGTTCTCCGGCCGCTTGGGTGGAGAGGCTATTCGGCTATGACTGGG
CACAACAGACAATCGGCTGCTCTGATGCCGCCGTGTTCCGGCTGTCAGCGCAGGGGCGCCCGGTTCTTTTTGTCA
AGACCGACCTGTCCGGTGCCCTGAATGAACTCCAGGACGAGGCAGCGCGGCTATCGTGGCTGGCCACGACGGGCG
TTCCTTGCGCAGCTGTGCTCGACGTTGTCACTGAAGCGGGAAGGGACTGGCTGCTATTGGGCGAAGTGCCGGGGC
AGGATCTCCTGTCATCTCACCTTGCTCCTGCCGAGAAAGTATCCATCATGGCTGATGCAATGCGGCGGCTGCATA
CGCTTGATCCGGCTACCTGCCCATTCGACCACCAAGCGAAACATCGCATCGAGCGAGCACGTACTCGGATGGAAG
CCGGTCTTGTCGATCAGGATGATCTGGACGAAGAGCATCAGGGGCTCGCGCCAGCCGAACTGTTCGCCAGGCTCA
AGGCGCGCATGCCCGACGGCGAGGATCTCGTCGTGACACATGGCGATGCCTGCTTGCCGAATATCATGGTGGAAA
ATGGCCGCTTTTCTGGATTCATCGACTGTGGCCGGCTGGGTGTGGCGGACCGCTATCAGGACATAGCGTTGGCTA
CCCGTGATATTGCTGAAGAGCTTGGCGGCGAATGGGCTGACCGCTTCCTCGTGCTTTACGGTATCGCCGCTCCCG
ATTCGCAGCGCATCGCCTTCTATCGCCTTCTTGACGAGTTCTTCTGAG

>lcl|X03043 Escherichia|aadA|82-870
TGAGGGAAGCGGTGATCGCCGAAGTATCGACTCAACTATCAGAGGTAGTTGGCGTCATCGAGCGCCATCTCGAAC
CGACGTTGCTGGCCGTACATTTGTACGGCTCCGCAGTGGATGGCGGCCTGAAGCCACACAGTGATATTGATTTGC
TGGTTACGGTGACCGTAAGGCTTGATGAAACAACGCGGCGAGCTTTGATCAACGACCTTTTGGAAACTTCGGCTT
CCCCTGGAGAGAGCGAGATTCTCCGCGCTGTAGAAGTCACCATTGTTGTGCACGACGACATCATTCCGTGGCGTT
ATCCAGCTAAGCGCGAACTGCAATTTGGAGAATGGCAGCGCAATGACATTCTTGCAGGTATCTTCGAGCCAGCCA
CGATCGACATTGATCTGGCTATCTTGCTGACAAAAGCAAGAGAACATAGCGTTGCCTTGGTAGGTCCAGCGGCGG
AGGAACTCTTTGATCCGGTTCCTGAACAGGATCTATTTGAGGCGCTAAATGAAACCTTAACGCTATGGAACTCGC
CGCCCGACTGGGCTGGCGATGAGCGAAATGTAGTGCTTACGTTGTCCCGCATTTGGTACAGCGCAGTAACCGGCA
AAATCGCGCCGAAGGATGTCGCTGCCGACTGGGCAATGGAGCGCCTGCCGGCCCAGTATCAGCCCGTCATACTTG
AAGCTAGGCAGGCTTATCTTGGACAAGAAGATCGCTTGGCCTCGCGCGCAGATCAGTTGGAAGAATTTGTTCACT
ACGTGAAAGGCGAGATCACCAAGGTAGTCGGCAAATAAT

>lcl|DQ666283 pRGK367|7S_UTR|3149-3521
TGTAGTACTAAAATGTATGCTGTAATAGCTCATAGTGAGCGAGGAAAGTATCGGGCTATTTAACTATGACTTGAG
CTCCATCTATGAATAAATAAATCAGCATATGATGCTTTTGTTTTGTGTACTTCAACTGTCTGCTTAGCTAATTTG
ATATGGTTGGCACTTGGCACGTATAAATATGCTGAAGTAATTTACTCTGAAGCTAAATTAACTAGATTAGATGAG
TGTATTATATACAAAAGGCATTAAATCAGATACATCTTAGACAAATTGTCACGGTCTACCAGAAAAGAAATTGCA
TTTGTTTTTGGGTCTTTCAGACTGACAAGATCGATCTGAAGTCTAAACAATTCTAAGAGGTATCATGTAGCAA

>lcl|AX600170 MON531|Partial_cry1Ac|310-1201
GCAGAGAAGAAGTGGAGGGACAAACGTGAGAAACTCGAATGGGAAACTAACATCGTTTACAAGGAGGCCAAAGAG
TCCGTGGATGCTTTGTTCGTGAACTCCCAATATGATCAGTTGCAAGCCGACACCAACATCGCCATGATCCACGCC
GCAGACAAACGTGTGCACAGCATTCGTGAGGCTTACTTGCCTGAGTTGTCCGTGATCCCTGGTGTGAACGCTGCC
ATCTTCGAGGAACTTGAGGGACGTATCTTTACCGCATTCTCCTTGTACGATGCCAGAAACGTCATCAAGAACGGT
GACTTCAACAATGGCCTCAGCTGCTGGAATGTGAAAGGTCATGTGGACGTGGAGGAACAGAACAATCAGCGTTCC
GTCCTGGTTGTGCCTGAGTGGGAAGCTGAAGTGTCCCAAGAGGTTAGAGTCTGTCCAGGTAGAGGCTACATTCTC
CGTGTGACCGCTTACAAGGAGGGATACGGTGAGGGTTGCGTGACCATCCACGAGATCGAGAACAACACCGACGAG
CTTAAGTTCTCCAACTGCGTCGAGGAAGAAATCTATCCCAACAACACCGTTACTTGCAACGACTACACTGTGAAT
CAGGAAGAGTACGGAGGTGCCTACACTAGCCGTAACAGAGGTTACAACGAAGCTCCTTCCGTTCCTGCTGACTAT
GCCTCCGTGTACGAGGAGAAATCCTACACAGATGGCAGACGTGAGAACCCTTGCGAGTTCAACAGAGGTTACAGG

```
GACTACACACCACTTCCAGTTGGCTATGTTACCAAGGAGCTTGAGTACTTTCCTGAGACCGACAAAGTGTGGATC
GAGATCGGTGAAACCGAGGGAACCTTCATCGTGGACAGCGTGGAGCTTCTCTTGATGGAGGAATAAT

>lcl|AX600188 MON531|3'Cotton|742-1121
CTGCCGAATCAACTAGCCCCGAAAATGGATGGCGCTTAAGCGCGCGACCTATACCCGGCCGTCGGGGCAAGGGCC
AGGCCCCGATGAGTAGGAGGGCGCGGCGGTCGCCGCAAAACCCGGGGCGCGAGCCCGGGCGGAGCGGCCGTCGGT
GCAGATCTTGGTGGTAGTAGCAAATATTCAAATGAGAACTTTGAAGGCCGAAGAGGGGAAAGGTTCCATGTGAAC
GGCACTTGCACATGGGTTAGTCGATCCTAAGAGACGGGGGAAGCCCGTCCGACAGCGCGTCCAGCGCGAGCTTCG
AAAGGGAATCGGGTTAAAATTCCTGAACCGGGACGCGGCGGCTGACGGCAACGTTAGGGAGTCCGGAGACATCTG
AAGC
```

Data S6. CCS reads with element orders tNOS – gap_- *Npt II* – gap_- p35S (133951, 45207), and p35S – gap_– tNOS – gap_– *uidA* (156962). p35S promoter is coloured yellow, tNOS terminator green *Npt II* gene red and *uidA* gene purple.

```
> 133951
ATATGATAATCATCGCAAGACCGGCAACAGGATTCAATCTTAAGAAACTTTATTGCCAAATGTTTGAACGATCGG
GGATCGGGGGTGGGCGAAGAACTCCAGCATGAGACCCCGCGCTGGAGGATCATCCAGCCGGCGTCCGGAAAACGA
TTCCGAGGCCCAACCTTTCATAGAAGGCGGCGGTGGAATCGAAATCTCGTGATGGCAGGTTGGGCGTCGCTTGGT
CGGCCATTTCGAACCCCAGAGTCCCGCTCAGAAGAACTCGTCAAGAAGGCGATAGAAGGCGATGCGCTGCGAATCG
GGAGCGGCGATACCGTAAAGCACGAGGAAGCGGTCAGCCATTCGCCGCCAAGCTCTTCAGCGATATCACGGGTAG
CCAACGCTATGTCCTGATAGCGGTCCGCCACACCCAGCCGGCCACAGTCGATGAATCCAGAAAAGCGGCCATTTT
CCACCATGATATTCGGCAAGCAGGCATCGCCATGGGTCACGACGAGATCCTCGCCGTCGGGCATGCGCGCCTTGA
GCCTGGCGAACAGTTCGGCTGGCGCGTGCCCCTGATGCTCTTCGTCCAGATCGTCCTGATCGACGAGACCGGCTT
CCATCCGAGTACGTGCTCGCTCGATGCGATGTTTCGCTTGGTGGTCGAATGGGCAGGTAGCCGGATCAAGCGTAT
GCAGCCGCCGCATTGCATCAGCCATGATGGATACTTTCTCGGCAGGAGCAAGGTGAGATGACAGGAGATCCTGCC
CCGGCACTTCGCCCAATAGCAGCCAGTCCCTTCCCGCTTCAGTGACAACGTCGAGCACAGCTGCGCAAGGAACGC
CCGTCGTGGCCAGCCACGATAGCCGCGCTGCCTCGTCCTGCAGTTCATTCAGGGCACCGGACAGGTCGGTCTTGA
CAAAAAGAACCGGGCGCCCTGCGCTGACAGCCGGAACACGGCGGCATCAGAGCAGCCGATTGTCTGTTGTGCCCA
GTCATAGCCGAATAGCCTCTCCACCCAAGCGGCCGGAGAACCTGCGTGCAATCCATCTTGTTCAATCATGCGAAAC
GATCGTCTAGCTAGAGATCCCGATCTTGTAGAGAGAGACTGGTGATTTCAGCGTGTCCTCTCCAAATGAAATGAA
CTTCCTTATATAGAGGAAGGGTCTTGCGATGATTATCATATA

> 45207
TATATGATAATCATCGCAAGACCCTTCCTCTATATAAGGAAGTTCATTTCATTTGGAGAGGACACGCTGAAATCA
CCAGTCTCTCTCTACAAGATCGGGGATCTCTAGCTAGACGATCGTTTCGCATGATTGAACAAGATGGATTGCACG
CAGGTTCTCCGGCCGCTTGGGTGGAGAGGCTATTCGGCTATGACTGGGCACAACAGACAATCGGCTGCTCTGATG
CCGCCGTGTTCCGGCTGTCAGCGCAGGGGCGCCCGGTTCTTTTTGTCAAGACCGACCTGTCCGGTGCCCTGAATG
AACTGCAGGACGAGGCAGCGCGGCTATCGTGGCTGGCCACGACGGGCGTTCCTTGCGCAGCTGTGCTCGACGTTG
TCACTGAAGCGGGAAGGGACTGGCTGCTATAGGGCGAAGTGCCGGGGCAGGATCTCCTGTCATCTCACCTTGCTC
CTGCCGAGAAAGTATCCATCATGGCTGATGCAATGCGGCGGCTGCATACGCTTGATCCGGCTACCTGCCCATTCG
ACCACCAAGCGAAACATCGCATCGAGCGAGCACGTACTCGGATGGAAGCCGGTCTTGTCGATCAGGATGATCTGG
ACGAAGAGCATCAGGGGCTCGCGCCAGCCGAACTGTTCGCCAGGCTCAAGGCGCGCATGCCCGACGGCGAGGATC
TCGTCGTGACCCATGGCGATGCCTGCTTGCCGAATATCATGGTGGAAAATGGCCGCTTTTCTGGATTCATCGACT
GTGGCCGGCTGGGTGTGGCGGACCGCTATCAGGACATAGCGTTGGCTACCCGTGATATTGCTGAAGAGCTTGGCG
GCGAATGGGCTGACCGCTTCCTCGTGCTTTACGGTATCGCCGCTCCCGATTCGCAGCGCATCGCCTTCTATCGCC
TTCTTGACGAGTTCTTCTGAGCGGGACTCTGGGGTTCGAAATGACCGACCAAGCGACGCCCAACCTGCCATCACG
AGATTTCGATTCCACCGCCGCCTTCTATGAAAGGTTGGGCTTCGGAATCGTTTTCCGGGACGCCGGCTGGATGAT
CCTCCAGCGCGGGGATCTCATGCTGGAGTTCTTCGCCCACCCCCCGGATCCCCGATCGTTCAAACATTTGGCAAT
AAAGTTTCTTAAGATTGAATCCTGTTGCCGGTCTTGCGATGATTATCATATA

> 156962
GGTCTTGCGAAGGATAGTGGGATTGTGCGTCATCCCTTACGTCAGTGGAGATATCAATCAATCCACTTGCTTTGA
AGACGTGGTTGGAACGTCTTCTTTTTCCACGATGCTCCTCGTGGGTGGGGGTCCATCTTTGGGACCACTGTCGCA
GAGGCATCTTCAACGATGGCCTTTCCTTTATCGCAATGATGGCATTTGTAGGAGCCACCTTCCTTTTCCACTATC
TTCACAATAAAGTGACAGATAGCTGGGCAATGGAATCCGAGGAGGTCTCCGGATATTACCCTTTGTTGAAAAGTC
TCAATCGGACCATCACATCAATCCACTTGCTTTGAAGACGTGGTTGGAACGTCTTCTTTTTCCACGATGCTCCTC
GTGGGTGGGGGTCCATCTTTGGACCACTGTCGGCAGAGGCATCTTCAACGATGGCCTTTCCTTTATCGCAATGAT
GGCATTTGTAGGAGCCACCTTCCTTTTCCACTATCTTCACAATAAAGTGACAGATAGCTGGGCAATGGAATCCGA
GGAGGTTTCCGGATATTACCCTTTGTTGAAAAGTCTCAATCGGACCTGCAGTTAACGCGGCCGCCACCGCGGTGG
AGCTCGCTAGCCATATGGGGCCGCCCGGGGATCCCCGATCTAGTAACATAGATGACACCGCGCGCGATAATTTAT
CCTAGTTTGCGCGCTATATTTTGTTTTCTATCGCGTATTAAATGTATAATTGCGGGACTCTAATCATAAAAACCC
ATCTCATAAATAACGTCATGCATTACATGTTAATTATTACATGCTTAACGTAATTCAACAGAAATTATATGATA
ATCATCGCAAGACCGGCAACAGGATTCAATCTTAAGAAACTTTATTGCCAAATGTTTGAACGATCGGGAATTCCC
GAGGCTGTAGCCGACGATGGTGCGCCAGGAGAGTTGTTGATTCATTGTTTGCCTCCCTGCTGCGGTTTTTCACCG
AAGTTCATGCCAGTCCAGCGTTTTTGCAGCAGAAAAGCCGCTGACTTCGGTTTGCGGTCGCGAGTGAAGGTCCCT
TTCTTGTTACCGCCAACGCGCAATATGCCTTGCGAGGTCGCAAAATCGGCGAAATTCCATACCTGTTCACGACGA
CGGCGCTGACGCGATCAAAGACGCGGTGATACATATCCAGCCATGCACACTGATACTCTTCACTCCACATGTCGG
TGTACATTGAGTGCAGCCCGGCTAACGTATCCACGCCGTATTCGGTGATGATAATCGGCTGATGCAGTTTCTCCT
GCCAGGCCAGAAGTTCTTTTTCCAGTACCTTCTCTGCCGTTTCCAAATCGCCGCTTTGGACATACCATCCGTAAT
AACGGTTCAGGCACAGCACATCAAAGAGATCGCTGATGGTATCGGTGTGAGCGTCGCAGAACATTACATTGACGC
```

AGGTGATCGGACGCGTCGGGTCGAGTTTACGCGTTGCTTCCGCCAGTGGCGCGAAATATTCCCGTGCACCTTGCG
GACGGGTATCCGGTTCGTTGGCAATACTCCACATCACCACGCTTGGGTGGTTTTTGTCACGCGCTATCAGCTCTT
TAATCGCCTGTAAGTGCGCTTGCTGAGTTTCCCCGTTGACTGCCTCTTTGCTGTACAGTTCTTTCGGCTTGTTGC
CCGCTTCGAAACCAATGCCTAAAGAGAGGTTAAAGCCGACAGCAGCAGTTTCATCAATCACCACGATGCCATGTT
CATC

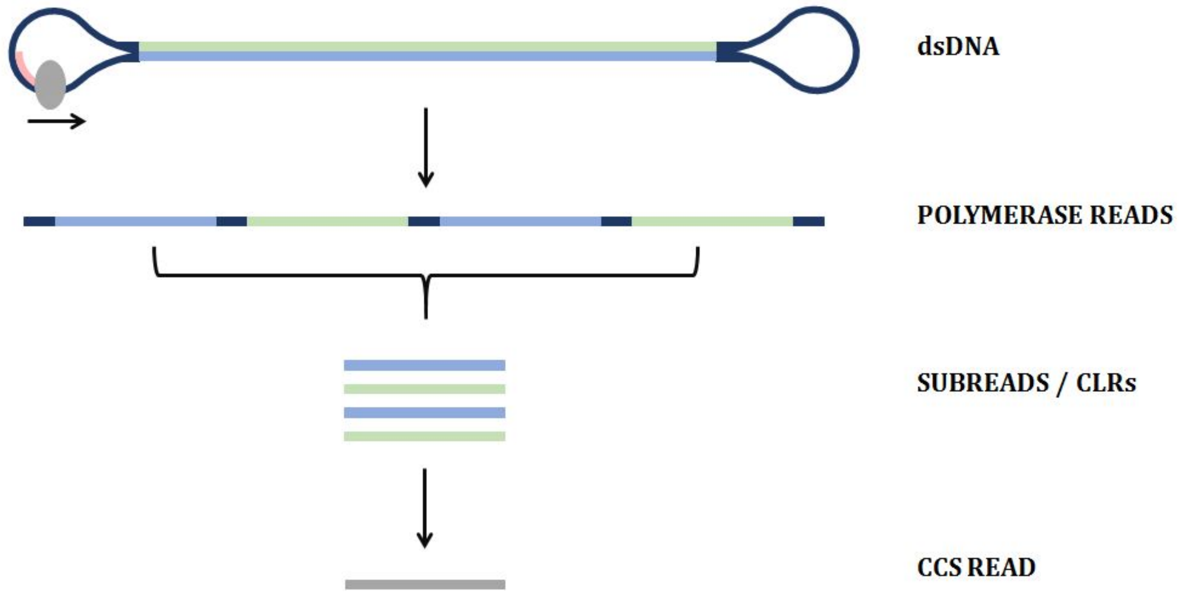

Figure S1. Formation of the circular consensus sequence (CCS) read. In PacBio sequencing a dsDNA molecule is sequenced. To do so, a single stranded loop is ligated to each side of the dsDNA (blue), a primer anneals to this loop (dark blue) and a strand displacement polymerase (grey) then passes the dsDNA. To gain a CCS reads the polymerase needs to pass the dsDNA template twice, the output is a so-called polymerase read, containing subreads interrupted by adapter sequences. The polymerase read is divided into subreads and the consensus of these subreads is a CCS read.

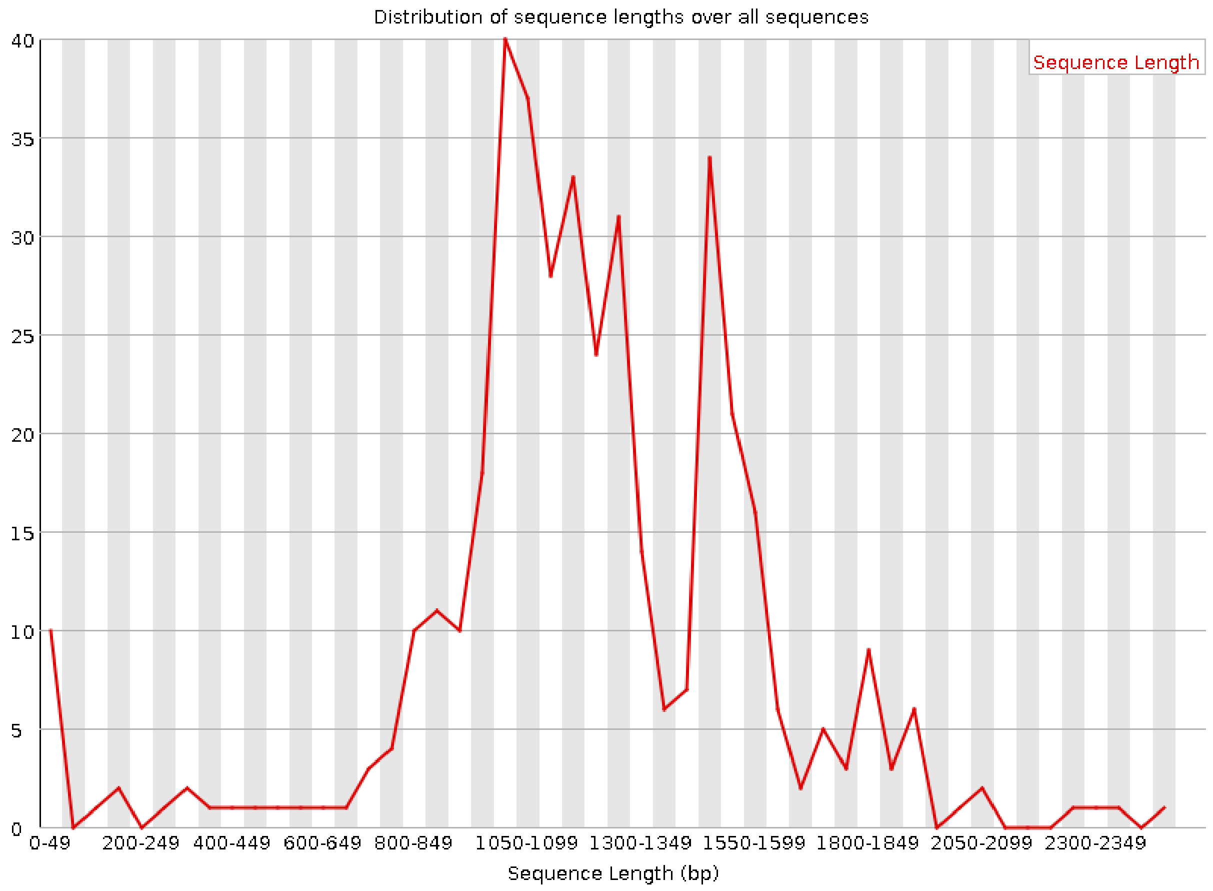

Figure S2. Length distribution of the 411 CCS reads as generated by FastQC**.**

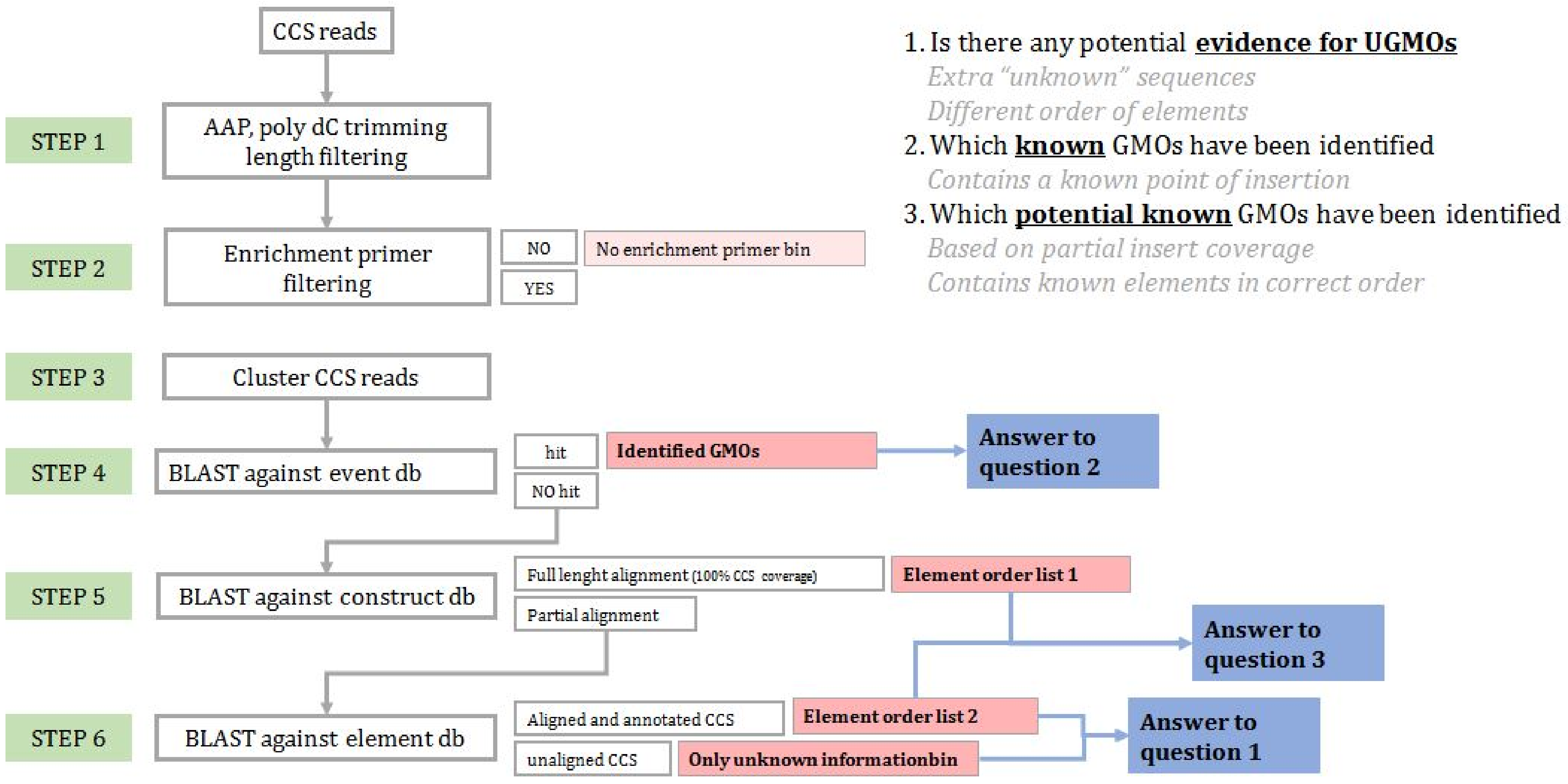

Figure S3. Building the workflow: in depth description. The workflow is divided in to two segments. First three steps prepare the reads for annotation in the following three steps. In linear enrichment (LE) process, a poly dC tail was added to 3' end of ssDNA acting as an annealing site for an abridged anchor primer or AAP, thus two-step trimming of poly dC tail and AAP sequence was performed with Cutadapt 1.8 in **Step 1**. First the AAP sequence was trimmed from 5' and a complement of AAP sequence from 3' prime end, followed by a removal of the poly dC and its complementary poly dG tail from 3' and 5' end,

respectively. For this Cutadapt 1.8 Galaxy wrapper was used, no parameters were changed for trimming of the AAP primers. To trim the poly dC and dG a string of 20 C/Gs was searched for at 3’ and 5’ end respectively, again no parameters of the trimming were changed. Input sequences were in FASTQ

format. Because very short sequences are not informative, as the shortest transgenic element in our element database is 60 bp, a length filtering, using filter FASTQ v1.0.0, was also performed parallel to the second trimming in **Step 1**. In LE a semi-nested PCR (snPCR) was performed using downstream semi-nested primers and upstream semi-nested primers for both 35S promoter and NOS terminator. Therefor CCS reads had one of these sequences (or their complements) present either on 5' end (semi-nested primer) or on 3' end (complement of semi-nested primer). This enabled an enrichment primer filtering in **Step 2**, with the purpose to only keep the sequences enriched for, and minimise the contamination. Sequences not containing semi-nested primers were not discarded completely but rather put in a *no enrichment primer* bin, if they would be needed in final data assessment. Enrichment primer filtering was also done using Cutadapt 1.8 tool, parameters were set to minimum overlap length of 19 nucleotides, which is at least 75% of the primer length (primers are from 21 to 25 nt long). Output options were set to *do not trim adapters* and to show also the untrimmed reads, which in this case means the sequences that do not contain any of the primers. Later sequences were moved to *no enrichment primer* bin. For further steps sequences were first translated from FASTQ to FASTA format using FASTQ to FASTA tool. In **Step 3** the remaining sequences were clustered using cluster OTUs tool. Cluster OTUs uses UPARSE-OTU algorithm, a greedy algorithm where high-abundance reads are more likely to be correct amplicon sequences. Therefor the CCS reads were first dereplicated, with criteria for dereplication being set at prefix, and sorted by abundance. A minimum cluster radius was set at 0.97, corresponding to a minimum of 97% sequence identity. CCS reads representing the clusters were annotated in the next 3 steps. As the Cluster OTUs tool does not recognise +/- orientation of the sequences, a reverse complement of all sequences that have enrichment primers at the 5' end was made using the reverse complement tool, to further decrease the number of the clusters. In **Steps 4** cluster representing CCS (crCCS) reads were blasted against the event database, using blastn in BLAST+ tool. Descriptions of Blast hits were modified to include information on the number of times the sequence in the event database aligns to the CCS read, CCS read ID and the name of the event, and transferred to c*onfirmed events*. In **Step 5** the remaining CCS reads were blasted against CAF database. Hits in CAF database were transferred to *only known information*, with the additional rule that the complete CCS read needed to be fully covered by a database sequence. In the last step, **Step 6**, the remaining sequences were blasted against elements database, and hits in element database were named *unknown parts*, as for CCS reads that did not match any of the criteria, they were named *only unknown information* and needed to be manually further processed. As *Element order Data 1* and *2* were are not in a user-friendly form, they were further processed in an excel template.

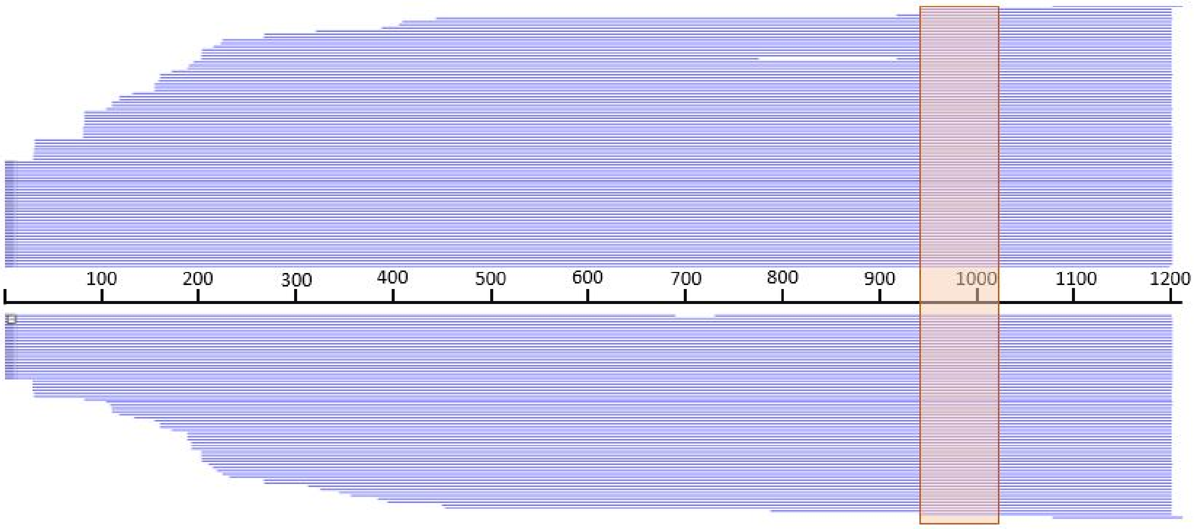

Figure S4. Bowtie2 mapping of CCS reads to RIKILT20151130 sequence. There is an overlap of 75 nucleotides when aligning sequences JQ406879 and AY326434. The overlap lies between nucleotides 943 and 1018 (orange) of the reference sequence RIKILT20151130. CCS reads were first filtered by quality, only those that had a quality score of 33 or more over at least 90% of the sequence were accepted. Using Bowtie2 mapping tool wrapper for Galaxy, with default settings, out of initial 411 sequences, 165 filtered sequences aligned to the reference, of those 133 CCS reads map to the overlap between sequences JQ406879 and AY326434 (orange).

References

1. EUginius - the European GMO reference database. Available at: http://www.euginius.eu/euginius/pages/home.jsf. (Accessed: 30th December 2016)
2. Van De Wiel, C. C. M. *et al.* Pollen-mediated gene flow in maize tested for coexistence of GM and non-GM crops in the Netherlands: effect of isolation distances between fields. *NJAS - Wageningen J. Life Sci.* **56,** 405–423 (2009).
3. Goecks, J., Nekrutenko, A. & Taylor, J. Galaxy: a comprehensive approach for supporting accessible, reproducible, and transparent computational research in the life sciences. *Genome biology* **11,** R86 (2010).
4. Giardine, B., Riemer, C. & Hardison, R. Galaxy: a platform for interactive large-scale genome analysis. *Genome Res.* **15,** 1451–1455 (2005).
5. Blankenberg, D. *et al.* in *Current Protocols in Molecular Biology* **Chapter 19,** Unit 19.10.1-21 (John Wiley & Sons, Inc., 2010).
6. Marin, M. Cutadapt removes adapter sequences from high-throughput sequencing reads. *EMBnet.journal* **17,** 10–12 (2011).
7. Quinlan, A. R. & Hall, I. M. BEDTools: a flexible suite of utilities for comparing genomic features. *Bioinformatics* **26,** 841–842 (2010).
8. Cock, PCock, P. J. A., Chilton, J. M., Grüning, B., Johnson, J. E., & Soranzo, N. (2015). N. B. integrated into G. bioRxiv. C. S. H. L. J. doi:10. 1101/014043ete. J. A., Chilton, J. M., Grüning, B., Johnson, J. E. & Soranzo, N. NCBI BLAST+ integrated into Galaxy. *bioRxiv* 14043 (2015). doi:10.1101/014043
9. Camacho, C. *et al.* BLAST plus : architecture and applications. *BMC Bioinformatics* **10,** (2009).
10. Edgar, R. C. UPARSE: highly accurate OTU sequences from microbial amplicon reads. *Nat Meth* **10,** 996–998 (2013).
11. Edgar, R. C. Search and clustering orders of magnitude faster than BLAST. *Bioinformatics* **26,** 2460–2461 (2010).
