# Supplementary material for: ALF: a strategy for identification of unauthorized GMOs in complex mixtures by a GW-NGS method and dedicated bioinformatics analysis
Source: Sci Rep. 2017 Oct 26;7:14155. doi: 10.1038/s41598-017-14469-8 (PMC5658351; doi:10.1038/s41598-017-14469-8)
Supplement: Supplementary file 2 — Supplementary pipeline [file 41598_2017_14469_MOESM2_ESM.doc]

ALF: a strategy for identification of unauthorized GMOs in complex mixtures by a GW-NGS method and dedicated bioinformatics analysis

Alexandra Bogožalec Košir1,2,‡, Alfred J. Arulandhu3,4,‡, Marleen M. Voorhuijzen3, Hongmei Xiao,5, Rico Hagelaar3, Martijn Staats3, Adalberto Costessi6, Jana Žel1, Esther J. Kok3, Jeroen P. van Dijk3*

1 Department of Biotechnology and Systems Biology, National Institute of Biology, Večna pot 111, SI-1000 Ljubljana, Slovenia

2 Jožef Stefan International Postgraduate School, Jamova 39, SI-1000 Ljubljana, Slovenia

3 RIKILT Wageningen UR, P.O. Box 230, 6700 AE Wageningen, The Netherlands

4 Food Quality and Design Group, Wageningen University and Research, P.O. Box 8129, 6700 EV Wageningen, The Netherlands.

5 College of Food Science and Technology, Nanjing Agricultural University, Jiangsu, 210095, PR China

6 BaseClear, Einsteinweg 5, 2333 CC Leiden, The Netherlands

‡ These authors contributed equally to this study

* Correspondence should be addressed to: Jeroen P. van Dijk

RIKILT Wageningen UR,

P.O. Box 230,

6700 AE Wageningen,

The Netherlands

Tel: +31-(0)6-23918347

Email: jeroen.vandijk@wur.nl

Read me file:

This is a Galaxy project workflow. To import it to Galaxy save the code below in a .txt file. The .txt can be imported in to Galaxy by choosing the upload or import workflow

in the workflow pannel (second tab from the left in Galaxy). Then choose the supplementary pipeline file and click import.

{

"a_galaxy_workflow": "true",

"annotation": "NGS data analysis after linear enrichment",

"format-version": "0.1",

"name": "clustering_v2_rev. comp_3'",

"steps": {

"0": {

"annotation": "QC_AAP trimming",

"id": 0,

"input_connections": {},

"inputs": [],

"name": "Cutadapt",

"outputs": [

{

"name": "report",

"type": "txt"

},

{

"name": "output",

"type": "fastq"

},

{

"name": "paired_output",

"type": "fastq"

},

{

"name": "rest_output",

"type": "fastq"

},

{

"name": "wild_output",

"type": "input"

},

{

"name": "too_short_output",

"type": "fastq"

},

{

"name": "too_long_output",

"type": "fastq"

},

{

"name": "untrimmed_output",

"type": "fastq"

},

{

"name": "untrimmed_paired_output",

"type": "fastq"

},

{

"name": "info_file",

"type": "input"

}

],

"position": {

"left": 199.97222900390625,

"top": 280.52779388427734

},

"post_job_actions": {

"HideDatasetActioninfo_file": {

"action_arguments": {},

"action_type": "HideDatasetAction",

"output_name": "info_file"

},

"HideDatasetActionpaired_output": {

"action_arguments": {},

"action_type": "HideDatasetAction",

"output_name": "paired_output"

},

"HideDatasetActionrest_output": {

"action_arguments": {},

"action_type": "HideDatasetAction",

"output_name": "rest_output"

},

"HideDatasetActiontoo_long_output": {

"action_arguments": {},

"action_type": "HideDatasetAction",

"output_name": "too_long_output"

},

"HideDatasetActiontoo_short_output": {

"action_arguments": {},

"action_type": "HideDatasetAction",

"output_name": "too_short_output"

},

"HideDatasetActionuntrimmed_output": {

"action_arguments": {},

"action_type": "HideDatasetAction",

"output_name": "untrimmed_output"

},

"HideDatasetActionuntrimmed_paired_output": {

"action_arguments": {},

"action_type": "HideDatasetAction",

"output_name": "untrimmed_paired_output"

},

"HideDatasetActionwild_output": {

"action_arguments": {},

"action_type": "HideDatasetAction",

"output_name": "wild_output"

},

"RenameDatasetActionoutput": {

"action_arguments": {

"newname": "AAP_trimed"

},

"action_type": "RenameDatasetAction",

"output_name": "output"

}

},

"tool_errors": null,

"tool_id": "testtoolshed.g2.bx.psu.edu/repos/slegras/cutadapt_1_8/cutadapt/1.8",

"tool_state": "{\"count\": \"\\\"1\\\"\", \"error_rate\": \"\\\"0.1\\\"\", \"match_read_wildcards\": \"\\\"False\\\"\", \"paired_end\": \"{\\\"paired_end_boolean\\\": \\\"False\\\", \\\"__current_case__\\\": 1}\", \"__page__\": 0, \"output_params\": \"{\\\"output_type\\\": \\\"default\\\", \\\"__current_case__\\\": 0}\", \"__rerun_remap_job_id__\": null, \"overlap\": \"\\\"3\\\"\", \"front_adapters\": \"[{\\\"__index__\\\": 0, \\\"front_adapter_source\\\": {\\\"front_adapter\\\": \\\"GGCCACGCGTCGACTAGTAC\\\", \\\"front_adapter_name\\\": \\\"AAP\\\", \\\"__current_case__\\\": 0, \\\"front_adapter_source_list\\\": \\\"user\\\"}}]\", \"input\": \"null\", \"no_indels\": \"\\\"False\\\"\", \"anywhere_adapters\": \"[]\", \"adapters\": \"[{\\\"__index__\\\": 0, \\\"adapter_source\\\": {\\\"adapter\\\": \\\"GTACTAGTCGACGCGTGGCC\\\", \\\"adapter_source_list\\\": \\\"user\\\", \\\"__current_case__\\\": 0, \\\"adapter_name\\\": \\\"compAAP\\\"}}]\", \"read_modification_params\": \"{\\\"read_modification\\\": \\\"none\\\", \\\"__current_case__\\\": 0}\", \"output_filtering_options\": \"{\\\"output_filtering\\\": \\\"default\\\", \\\"__current_case__\\\": 0}\"}",

"tool_version": "1.8",

"type": "tool",

"user_outputs": []

},

"1": {

"annotation": "",

"id": 1,

"input_connections": {

"input": {

"id": 0,

"output_name": "output"

}

},

"inputs": [],

"name": "Cutadapt",

"outputs": [

{

"name": "report",

"type": "txt"

},

{

"name": "output",

"type": "fastq"

},

{

"name": "paired_output",

"type": "fastq"

},

{

"name": "rest_output",

"type": "fastq"

},

{

"name": "wild_output",

"type": "input"

},

{

"name": "too_short_output",

"type": "fastq"

},

{

"name": "too_long_output",

"type": "fastq"

},

{

"name": "untrimmed_output",

"type": "fastq"

},

{

"name": "untrimmed_paired_output",

"type": "fastq"

},

{

"name": "info_file",

"type": "input"

}

],

"position": {

"left": 535.9791870117188,

"top": 274.5208511352539

},

"post_job_actions": {

"HideDatasetActioninfo_file": {

"action_arguments": {},

"action_type": "HideDatasetAction",

"output_name": "info_file"

},

"HideDatasetActionpaired_output": {

"action_arguments": {},

"action_type": "HideDatasetAction",

"output_name": "paired_output"

},

"HideDatasetActionrest_output": {

"action_arguments": {},

"action_type": "HideDatasetAction",

"output_name": "rest_output"

},

"HideDatasetActiontoo_long_output": {

"action_arguments": {},

"action_type": "HideDatasetAction",

"output_name": "too_long_output"

},

"HideDatasetActiontoo_short_output": {

"action_arguments": {},

"action_type": "HideDatasetAction",

"output_name": "too_short_output"

},

"HideDatasetActionuntrimmed_output": {

"action_arguments": {},

"action_type": "HideDatasetAction",

"output_name": "untrimmed_output"

},

"HideDatasetActionuntrimmed_paired_output": {

"action_arguments": {},

"action_type": "HideDatasetAction",

"output_name": "untrimmed_paired_output"

},

"HideDatasetActionwild_output": {

"action_arguments": {},

"action_type": "HideDatasetAction",

"output_name": "wild_output"

},

"RenameDatasetActionoutput": {

"action_arguments": {

"newname": "pC_pG_trimed"

},

"action_type": "RenameDatasetAction",

"output_name": "output"

}

},

"tool_errors": null,

"tool_id": "testtoolshed.g2.bx.psu.edu/repos/slegras/cutadapt_1_8/cutadapt/1.8",

"tool_state": "{\"count\": \"\\\"1\\\"\", \"error_rate\": \"\\\"0.1\\\"\", \"match_read_wildcards\": \"\\\"False\\\"\", \"paired_end\": \"{\\\"paired_end_boolean\\\": \\\"False\\\", \\\"__current_case__\\\": 1}\", \"__page__\": 0, \"output_params\": \"{\\\"output_type\\\": \\\"default\\\", \\\"__current_case__\\\": 0}\", \"__rerun_remap_job_id__\": null, \"overlap\": \"\\\"3\\\"\", \"front_adapters\": \"[{\\\"__index__\\\": 0, \\\"front_adapter_source\\\": {\\\"front_adapter\\\": \\\"GGGGGGGGGGGGGGGGGGGG\\\", \\\"front_adapter_name\\\": \\\"pG_tail\\\", \\\"__current_case__\\\": 0, \\\"front_adapter_source_list\\\": \\\"user\\\"}}]\", \"input\": \"null\", \"no_indels\": \"\\\"False\\\"\", \"anywhere_adapters\": \"[]\", \"adapters\": \"[{\\\"__index__\\\": 0, \\\"adapter_source\\\": {\\\"adapter\\\": \\\"CCCCCCCCCCCCCCCCCCCC\\\", \\\"adapter_source_list\\\": \\\"user\\\", \\\"__current_case__\\\": 0, \\\"adapter_name\\\": \\\"pC_tail\\\"}}]\", \"read_modification_params\": \"{\\\"read_modification\\\": \\\"none\\\", \\\"__current_case__\\\": 0}\", \"output_filtering_options\": \"{\\\"min\\\": \\\"50\\\", \\\"max\\\": \\\"0\\\", \\\"discard_untrimmed\\\": \\\"False\\\", \\\"mask_adapter\\\": \\\"False\\\", \\\"no_trim\\\": \\\"False\\\", \\\"__current_case__\\\": 1, \\\"output_filtering\\\": \\\"filter\\\", \\\"discard\\\": \\\"False\\\"}\"}",

"tool_version": "1.8",

"type": "tool",

"user_outputs": []

},

"2": {

"annotation": "",

"id": 2,

"input_connections": {

"input": {

"id": 1,

"output_name": "output"

}

},

"inputs": [],

"name": "Cutadapt",

"outputs": [

{

"name": "report",

"type": "txt"

},

{

"name": "output",

"type": "fastq"

},

{

"name": "paired_output",

"type": "fastq"

},

{

"name": "rest_output",

"type": "fastq"

},

{

"name": "wild_output",

"type": "input"

},

{

"name": "too_short_output",

"type": "fastq"

},

{

"name": "too_long_output",

"type": "fastq"

},

{

"name": "untrimmed_output",

"type": "fastq"

},

{

"name": "untrimmed_paired_output",

"type": "fastq"

},

{

"name": "info_file",

"type": "input"

}

],

"position": {

"left": 871.2743225097656,

"top": 281.7430725097656

},

"post_job_actions": {

"HideDatasetActioninfo_file": {

"action_arguments": {},

"action_type": "HideDatasetAction",

"output_name": "info_file"

},

"HideDatasetActionpaired_output": {

"action_arguments": {},

"action_type": "HideDatasetAction",

"output_name": "paired_output"

},

"HideDatasetActionrest_output": {

"action_arguments": {},

"action_type": "HideDatasetAction",

"output_name": "rest_output"

},

"HideDatasetActiontoo_long_output": {

"action_arguments": {},

"action_type": "HideDatasetAction",

"output_name": "too_long_output"

},

"HideDatasetActiontoo_short_output": {

"action_arguments": {},

"action_type": "HideDatasetAction",

"output_name": "too_short_output"

},

"HideDatasetActionuntrimmed_paired_output": {

"action_arguments": {},

"action_type": "HideDatasetAction",

"output_name": "untrimmed_paired_output"

},

"HideDatasetActionwild_output": {

"action_arguments": {},

"action_type": "HideDatasetAction",

"output_name": "wild_output"

},

"RenameDatasetActionoutput": {

"action_arguments": {

"newname": "enrichment primer filtered"

},

"action_type": "RenameDatasetAction",

"output_name": "output"

},

"RenameDatasetActionuntrimmed_output": {

"action_arguments": {

"newname": "bin1_no enrichment primer"

},

"action_type": "RenameDatasetAction",

"output_name": "untrimmed_output"

}

},

"tool_errors": null,

"tool_id": "testtoolshed.g2.bx.psu.edu/repos/slegras/cutadapt_1_8/cutadapt/1.8",

"tool_state": "{\"count\": \"\\\"1\\\"\", \"error_rate\": \"\\\"0.1\\\"\", \"match_read_wildcards\": \"\\\"False\\\"\", \"paired_end\": \"{\\\"paired_end_boolean\\\": \\\"False\\\", \\\"__current_case__\\\": 1}\", \"__page__\": 0, \"output_params\": \"{\\\"__current_case__\\\": 1, \\\"wildcard_file\\\": \\\"False\\\", \\\"too_long_file\\\": \\\"False\\\", \\\"too_short_file\\\": \\\"False\\\", \\\"rest_file\\\": \\\"False\\\", \\\"untrimmed_file\\\": \\\"True\\\", \\\"output_type\\\": \\\"additional\\\", \\\"info_file\\\": \\\"False\\\"}\", \"__rerun_remap_job_id__\": null, \"overlap\": \"\\\"15\\\"\", \"front_adapters\": \"[{\\\"__index__\\\": 0, \\\"front_adapter_source\\\": {\\\"front_adapter\\\": \\\"^TATATGATAATCATCGCAAGAC\\\", \\\"front_adapter_name\\\": \\\"tNOS_as3\\\", \\\"__current_case__\\\": 0, \\\"front_adapter_source_list\\\": \\\"user\\\"}}, {\\\"__index__\\\": 1, \\\"front_adapter_source\\\": {\\\"front_adapter\\\": \\\"^CGATAGAAAACAAAATATAGCG\\\", \\\"front_adapter_name\\\": \\\"tNOS_s3\\\", \\\"__current_case__\\\": 0, \\\"front_adapter_source_list\\\": \\\"user\\\"}}, {\\\"__index__\\\": 2, \\\"front_adapter_source\\\": {\\\"front_adapter\\\": \\\"^AGGAAGTTCATTTCATTTGGAGAGG\\\", \\\"front_adapter_name\\\": \\\"p35S_s3\\\", \\\"__current_case__\\\": 0, \\\"front_adapter_source_list\\\": \\\"user\\\"}}, {\\\"__index__\\\": 3, \\\"front_adapter_source\\\": {\\\"front_adapter\\\": \\\"^GGTCTTGCGAAGGATAGTGGG\\\", \\\"front_adapter_name\\\": \\\"p35S_as2\\\", \\\"__current_case__\\\": 0, \\\"front_adapter_source_list\\\": \\\"user\\\"}}]\", \"input\": \"null\", \"no_indels\": \"\\\"False\\\"\", \"anywhere_adapters\": \"[]\", \"adapters\": \"[{\\\"__index__\\\": 1, \\\"adapter_source\\\": {\\\"adapter\\\": \\\"CGCTATATTTTGTTTTCTATCG$\\\", \\\"adapter_source_list\\\": \\\"user\\\", \\\"__current_case__\\\": 0, \\\"adapter_name\\\": \\\"tNOS_as1\\\"}}, {\\\"__index__\\\": 2, \\\"adapter_source\\\": {\\\"adapter\\\": \\\"CCTCTCCAAATGAAATGAACTTCCT$\\\", \\\"adapter_source_list\\\": \\\"user\\\", \\\"__current_case__\\\": 0, \\\"adapter_name\\\": \\\"p35S_as1\\\"}}, {\\\"__index__\\\": 3, \\\"adapter_source\\\": {\\\"adapter\\\": \\\"CCCACTATCCTTCGCAAGACC$\\\", \\\"adapter_source_list\\\": \\\"user\\\", \\\"__current_case__\\\": 0, \\\"adapter_name\\\": \\\"p35S_s2\\\"}}, {\\\"__index__\\\": 4, \\\"adapter_source\\\": {\\\"adapter\\\": \\\"GTCTTGCGATGATTATCATATA$\\\", \\\"adapter_source_list\\\": \\\"user\\\", \\\"__current_case__\\\": 0, \\\"adapter_name\\\": \\\"tNOS_s1\\\"}}]\", \"read_modification_params\": \"{\\\"read_modification\\\": \\\"none\\\", \\\"__current_case__\\\": 0}\", \"output_filtering_options\": \"{\\\"min\\\": \\\"0\\\", \\\"max\\\": \\\"0\\\", \\\"discard_untrimmed\\\": \\\"False\\\", \\\"mask_adapter\\\": \\\"False\\\", \\\"no_trim\\\": \\\"True\\\", \\\"__current_case__\\\": 1, \\\"output_filtering\\\": \\\"filter\\\", \\\"discard\\\": \\\"False\\\"}\"}",

"tool_version": "1.8",

"type": "tool",

"user_outputs": []

},

"3": {

"annotation": "",

"id": 3,

"input_connections": {

"input": {

"id": 2,

"output_name": "output"

}

},

"inputs": [],

"name": "Cutadapt",

"outputs": [

{

"name": "report",

"type": "txt"

},

{

"name": "output",

"type": "fastq"

},

{

"name": "paired_output",

"type": "fastq"

},

{

"name": "rest_output",

"type": "fastq"

},

{

"name": "wild_output",

"type": "input"

},

{

"name": "too_short_output",

"type": "fastq"

},

{

"name": "too_long_output",

"type": "fastq"

},

{

"name": "untrimmed_output",

"type": "fastq"

},

{

"name": "untrimmed_paired_output",

"type": "fastq"

},

{

"name": "info_file",

"type": "input"

}

],

"position": {

"left": 1197.2118835449219,

"top": 278.7396011352539

},

"post_job_actions": {

"HideDatasetActioninfo_file": {

"action_arguments": {},

"action_type": "HideDatasetAction",

"output_name": "info_file"

},

"HideDatasetActionpaired_output": {

"action_arguments": {},

"action_type": "HideDatasetAction",

"output_name": "paired_output"

},

"HideDatasetActionrest_output": {

"action_arguments": {},

"action_type": "HideDatasetAction",

"output_name": "rest_output"

},

"HideDatasetActiontoo_long_output": {

"action_arguments": {},

"action_type": "HideDatasetAction",

"output_name": "too_long_output"

},

"HideDatasetActiontoo_short_output": {

"action_arguments": {},

"action_type": "HideDatasetAction",

"output_name": "too_short_output"

},

"HideDatasetActionuntrimmed_paired_output": {

"action_arguments": {},

"action_type": "HideDatasetAction",

"output_name": "untrimmed_paired_output"

},

"HideDatasetActionwild_output": {

"action_arguments": {},

"action_type": "HideDatasetAction",

"output_name": "wild_output"

},

"RenameDatasetActionoutput": {

"action_arguments": {

"newname": "5'_primer"

},

"action_type": "RenameDatasetAction",

"output_name": "output"

},

"RenameDatasetActionuntrimmed_output": {

"action_arguments": {

"newname": "3'_primer"

},

"action_type": "RenameDatasetAction",

"output_name": "untrimmed_output"

}

},

"tool_errors": null,

"tool_id": "testtoolshed.g2.bx.psu.edu/repos/slegras/cutadapt_1_8/cutadapt/1.8",

"tool_state": "{\"count\": \"\\\"1\\\"\", \"error_rate\": \"\\\"0.1\\\"\", \"match_read_wildcards\": \"\\\"False\\\"\", \"paired_end\": \"{\\\"paired_end_boolean\\\": \\\"False\\\", \\\"__current_case__\\\": 1}\", \"__page__\": 0, \"output_params\": \"{\\\"__current_case__\\\": 1, \\\"wildcard_file\\\": \\\"False\\\", \\\"too_long_file\\\": \\\"False\\\", \\\"too_short_file\\\": \\\"False\\\", \\\"rest_file\\\": \\\"False\\\", \\\"untrimmed_file\\\": \\\"True\\\", \\\"output_type\\\": \\\"additional\\\", \\\"info_file\\\": \\\"False\\\"}\", \"__rerun_remap_job_id__\": null, \"overlap\": \"\\\"15\\\"\", \"front_adapters\": \"[]\", \"input\": \"null\", \"no_indels\": \"\\\"False\\\"\", \"anywhere_adapters\": \"[]\", \"adapters\": \"[{\\\"__index__\\\": 0, \\\"adapter_source\\\": {\\\"adapter\\\": \\\"CCTCTCCAAATGAAATGAACTTCCT$\\\", \\\"adapter_source_list\\\": \\\"user\\\", \\\"__current_case__\\\": 0, \\\"adapter_name\\\": \\\"35S_as1\\\"}}, {\\\"__index__\\\": 1, \\\"adapter_source\\\": {\\\"adapter\\\": \\\"CCCACTATCCTTCGCAAGACC$\\\", \\\"adapter_source_list\\\": \\\"user\\\", \\\"__current_case__\\\": 0, \\\"adapter_name\\\": \\\"35S_s2\\\"}}, {\\\"__index__\\\": 2, \\\"adapter_source\\\": {\\\"adapter\\\": \\\"GTCTTGCGATGATTATCATATA$\\\", \\\"adapter_source_list\\\": \\\"user\\\", \\\"__current_case__\\\": 0, \\\"adapter_name\\\": \\\"tNOS_s1\\\"}}, {\\\"__index__\\\": 3, \\\"adapter_source\\\": {\\\"adapter\\\": \\\"CGCTATATTTTGTTTTCTATCG$\\\", \\\"adapter_source_list\\\": \\\"user\\\", \\\"__current_case__\\\": 0, \\\"adapter_name\\\": \\\"tNOS_as1\\\"}}]\", \"read_modification_params\": \"{\\\"read_modification\\\": \\\"none\\\", \\\"__current_case__\\\": 0}\", \"output_filtering_options\": \"{\\\"min\\\": \\\"0\\\", \\\"max\\\": \\\"0\\\", \\\"discard_untrimmed\\\": \\\"False\\\", \\\"mask_adapter\\\": \\\"False\\\", \\\"no_trim\\\": \\\"True\\\", \\\"__current_case__\\\": 1, \\\"output_filtering\\\": \\\"filter\\\", \\\"discard\\\": \\\"False\\\"}\"}",

"tool_version": "1.8",

"type": "tool",

"user_outputs": []

},

"4": {

"annotation": "",

"id": 4,

"input_connections": {

"input_file": {

"id": 3,

"output_name": "output"

}

},

"inputs": [],

"name": "FASTQ to FASTA",

"outputs": [

{

"name": "output_file",

"type": "fasta"

}

],

"position": {

"left": 1551.9167785644531,

"top": 288.94793701171875

},

"post_job_actions": {

"RenameDatasetActionoutput_file": {

"action_arguments": {

"newname": "enrichment primer filterd CCS"

},

"action_type": "RenameDatasetAction",

"output_name": "output_file"

}

},

"tool_errors": null,

"tool_id": "toolshed.g2.bx.psu.edu/repos/devteam/fastqtofasta/fastq_to_fasta_python/1.0.0",

"tool_state": "{\"__page__\": 0, \"__rerun_remap_job_id__\": null, \"input_file\": \"null\"}",

"tool_version": "1.0.0",

"type": "tool",

"user_outputs": []

},

"5": {

"annotation": "",

"id": 5,

"input_connections": {

"input_file": {

"id": 3,

"output_name": "untrimmed_output"

}

},

"inputs": [],

"name": "FASTQ to FASTA",

"outputs": [

{

"name": "output_file",

"type": "fasta"

}

],

"position": {

"left": 1549.7812805175781,

"top": 651.4305877685547

},

"post_job_actions": {},

"tool_errors": null,

"tool_id": "toolshed.g2.bx.psu.edu/repos/devteam/fastqtofasta/fastq_to_fasta_python/1.0.0",

"tool_state": "{\"__page__\": 0, \"__rerun_remap_job_id__\": null, \"input_file\": \"null\"}",

"tool_version": "1.0.0",

"type": "tool",

"user_outputs": []

},

"6": {

"annotation": "",

"id": 6,

"input_connections": {

"input": {

"id": 4,

"output_name": "output_file"

}

},

"inputs": [],

"name": "reverse complement",

"outputs": [

{

"name": "output",

"type": "input"

}

],

"position": {

"left": 1803.8959045410156,

"top": 275.54515075683594

},

"post_job_actions": {

"RenameDatasetActionoutput": {

"action_arguments": {

"newname": "5'_rev_comp"

},

"action_type": "RenameDatasetAction",

"output_name": "output"

}

},

"tool_errors": null,

"tool_id": "toolshed.g2.bx.psu.edu/repos/xuebing/reverse_complement/revcompl/1.0.0",

"tool_state": "{\"input\": \"null\", \"fasta\": \"\\\"True\\\"\", \"__rerun_remap_job_id__\": null, \"rna\": \"\\\"False\\\"\", \"__page__\": 0}",

"tool_version": "1.0.0",

"type": "tool",

"user_outputs": []

},

"7": {

"annotation": "",

"id": 7,

"input_connections": {

"input": {

"id": 5,

"output_name": "output_file"

}

},

"inputs": [],

"name": "FASTA-to-Tabular",

"outputs": [

{

"name": "output",

"type": "tabular"

}

],

"position": {

"left": 1802.9062805175781,

"top": 532.5590667724609

},

"post_job_actions": {

"RenameDatasetActionoutput": {

"action_arguments": {

"newname": "3'_tab"

},

"action_type": "RenameDatasetAction",

"output_name": "output"

}

},

"tool_errors": null,

"tool_id": "toolshed.g2.bx.psu.edu/repos/devteam/fasta_to_tabular/fasta2tab/1.1.0",

"tool_state": "{\"input\": \"null\", \"keep_first\": \"\\\"0\\\"\", \"__rerun_remap_job_id__\": null, \"descr_columns\": \"\\\"1\\\"\", \"__page__\": 0}",

"tool_version": "1.1.0",

"type": "tool",

"user_outputs": []

},

"8": {

"annotation": "",

"id": 8,

"input_connections": {

"input": {

"id": 6,

"output_name": "output"

}

},

"inputs": [],

"name": "FASTA-to-Tabular",

"outputs": [

{

"name": "output",

"type": "tabular"

}

],

"position": {

"left": 1800.9271545410156,

"top": 391.5868225097656

},

"post_job_actions": {

"RenameDatasetActionoutput": {

"action_arguments": {

"newname": "5'_rev_comp_tab"

},

"action_type": "RenameDatasetAction",

"output_name": "output"

}

},

"tool_errors": null,

"tool_id": "toolshed.g2.bx.psu.edu/repos/devteam/fasta_to_tabular/fasta2tab/1.1.0",

"tool_state": "{\"input\": \"null\", \"keep_first\": \"\\\"0\\\"\", \"__rerun_remap_job_id__\": null, \"descr_columns\": \"\\\"1\\\"\", \"__page__\": 0}",

"tool_version": "1.1.0",

"type": "tool",

"user_outputs": []

},

"9": {

"annotation": "",

"id": 9,

"input_connections": {

"input1": {

"id": 8,

"output_name": "output"

},

"queries_0|input2": {

"id": 7,

"output_name": "output"

}

},

"inputs": [],

"name": "Concatenate datasets",

"outputs": [

{

"name": "out_file1",

"type": "input"

}

],

"position": {

"left": 2098.496612548828,

"top": 489.67710876464844

},

"post_job_actions": {},

"tool_errors": null,

"tool_id": "cat1",

"tool_state": "{\"__page__\": 0, \"__rerun_remap_job_id__\": null, \"input1\": \"null\", \"queries\": \"[{\\\"input2\\\": null, \\\"__index__\\\": 0}]\"}",

"tool_version": "1.0.0",

"type": "tool",

"user_outputs": []

},

"10": {

"annotation": "",

"id": 10,

"input_connections": {

"input": {

"id": 9,

"output_name": "out_file1"

}

},

"inputs": [],

"name": "Tabular-to-FASTA",

"outputs": [

{

"name": "output",

"type": "fasta"

}

],

"position": {

"left": 2360.0939025878906,

"top": 487.75001525878906

},

"post_job_actions": {

"RenameDatasetActionoutput": {

"action_arguments": {

"newname": "3'_5'_rev_comp_combined"

},

"action_type": "RenameDatasetAction",

"output_name": "output"

}

},

"tool_errors": null,

"tool_id": "toolshed.g2.bx.psu.edu/repos/devteam/tabular_to_fasta/tab2fasta/1.1.0",

"tool_state": "{\"title_col\": \"{\\\"__class__\\\": \\\"UnvalidatedValue\\\", \\\"value\\\": [\\\"1\\\"]}\", \"input\": \"null\", \"__page__\": 0, \"__rerun_remap_job_id__\": null, \"seq_col\": \"{\\\"__class__\\\": \\\"UnvalidatedValue\\\", \\\"value\\\": \\\"2\\\"}\"}",

"tool_version": "1.1.0",

"type": "tool",

"user_outputs": []

},

"11": {

"annotation": "",

"id": 11,

"input_connections": {

"input": {

"id": 10,

"output_name": "output"

}

},

"inputs": [],

"name": "Derep_prefix",

"outputs": [

{

"name": "output",

"type": "fasta"

}

],

"position": {

"left": 2661.9515686035156,

"top": 469.1042022705078

},

"post_job_actions": {},

"tool_errors": null,

"tool_id": "Derep_prefix",

"tool_state": "{\"input\": \"null\", \"__rerun_remap_job_id__\": null, \"__page__\": 0}",

"tool_version": "1.0.0M",

"type": "tool",

"user_outputs": []

},

"12": {

"annotation": "",

"id": 12,

"input_connections": {

"input": {

"id": 11,

"output_name": "output"

}

},

"inputs": [],

"name": "sortbysize",

"outputs": [

{

"name": "output",

"type": "fasta"

}

],

"position": {

"left": 2662.0382385253906,

"top": 610.1458587646484

},

"post_job_actions": {},

"tool_errors": null,

"tool_id": "sortbysize",

"tool_state": "{\"__page__\": 0, \"input\": \"null\", \"__rerun_remap_job_id__\": null, \"size\": \"\\\"1\\\"\"}",

"tool_version": "v1.0.0",

"type": "tool",

"user_outputs": []

},

"13": {

"annotation": "",

"id": 13,

"input_connections": {

"input": {

"id": 12,

"output_name": "output"

}

},

"inputs": [],

"name": "Cluster OTU",

"outputs": [

{

"name": "output",

"type": "fasta"

}

],

"position": {

"left": 2665.9794006347656,

"top": 756.1007232666016

},

"post_job_actions": {

"RenameDatasetActionoutput": {

"action_arguments": {

"newname": "clustered_CCS"

},

"action_type": "RenameDatasetAction",

"output_name": "output"

}

},

"tool_errors": null,

"tool_id": "cluster_otu",

"tool_state": "{\"input\": \"null\", \"__rerun_remap_job_id__\": null, \"radius\": \"\\\"3\\\"\", \"__page__\": 0}",

"tool_version": "1.0.0M",

"type": "tool",

"user_outputs": []

},

"14": {

"annotation": "",

"id": 14,

"input_connections": {

"input": {

"id": 13,

"output_name": "output"

}

},

"inputs": [],

"name": "FASTA-to-Tabular",

"outputs": [

{

"name": "output",

"type": "tabular"

}

],

"position": {

"left": 2660.8231506347656,

"top": 875.7014312744141

},

"post_job_actions": {},

"tool_errors": null,

"tool_id": "toolshed.g2.bx.psu.edu/repos/devteam/fasta_to_tabular/fasta2tab/1.1.0",

"tool_state": "{\"input\": \"null\", \"keep_first\": \"\\\"0\\\"\", \"__rerun_remap_job_id__\": null, \"descr_columns\": \"\\\"1\\\"\", \"__page__\": 0}",

"tool_version": "1.1.0",

"type": "tool",

"user_outputs": []

},

"15": {

"annotation": "",

"id": 15,

"input_connections": {

"input": {

"id": 14,

"output_name": "output"

}

},

"inputs": [],

"name": "Convert",

"outputs": [

{

"name": "out_file1",

"type": "tabular"

}

],

"position": {

"left": 2902.6805725097656,

"top": 745.5625152587891

},

"post_job_actions": {},

"tool_errors": null,

"tool_id": "Convert characters1",

"tool_state": "{\"input\": \"null\", \"__rerun_remap_job_id__\": null, \"convert_from\": \"\\\"Sc\\\"\", \"__page__\": 0}",

"tool_version": "1.0.0",

"type": "tool",

"user_outputs": []

},

"16": {

"annotation": "",

"id": 16,

"input_connections": {

"input": {

"id": 15,

"output_name": "out_file1"

}

},

"inputs": [],

"name": "Tabular-to-FASTA",

"outputs": [

{

"name": "output",

"type": "fasta"

}

],

"position": {

"left": 2907.8717346191406,

"top": 889.7639312744141

},

"post_job_actions": {

"RenameDatasetActionoutput": {

"action_arguments": {

"newname": "CCS_clustered_renamed"

},

"action_type": "RenameDatasetAction",

"output_name": "output"

}

},

"tool_errors": null,

"tool_id": "toolshed.g2.bx.psu.edu/repos/devteam/tabular_to_fasta/tab2fasta/1.1.0",

"tool_state": "{\"title_col\": \"{\\\"__class__\\\": \\\"UnvalidatedValue\\\", \\\"value\\\": [\\\"1\\\"]}\", \"input\": \"null\", \"__page__\": 0, \"__rerun_remap_job_id__\": null, \"seq_col\": \"{\\\"__class__\\\": \\\"UnvalidatedValue\\\", \\\"value\\\": \\\"4\\\"}\"}",

"tool_version": "1.1.0",

"type": "tool",

"user_outputs": []

},

"17": {

"annotation": "",

"id": 17,

"input_connections": {

"query": {

"id": 16,

"output_name": "output"

}

},

"inputs": [],

"name": "NCBI BLAST+ blastn",

"outputs": [

{

"name": "output1",

"type": "tabular"

}

],

"position": {

"left": 3130.7535705566406,

"top": 618.0972442626953

},

"post_job_actions": {},

"tool_errors": null,

"tool_id": "toolshed.g2.bx.psu.edu/repos/devteam/ncbi_blast_plus/ncbi_blastn_wrapper/0.1.00",

"tool_state": "{\"evalue_cutoff\": \"\\\"0.001\\\"\", \"__page__\": 0, \"adv_opts\": \"{\\\"adv_opts_selector\\\": \\\"basic\\\", \\\"__current_case__\\\": 0}\", \"__rerun_remap_job_id__\": null, \"blast_type\": \"\\\"megablast\\\"\", \"db_opts\": \"{\\\"db_opts_selector\\\": \\\"db\\\", \\\"subject\\\": \\\"\\\", \\\"histdb\\\": \\\"\\\", \\\"__current_case__\\\": 0, \\\"database\\\": \\\"events.fasta\\\"}\", \"output\": \"{\\\"out_format\\\": \\\"ext\\\", \\\"__current_case__\\\": 1}\", \"query\": \"null\"}",

"tool_version": "0.1.00",

"type": "tool",

"user_outputs": []

},

"18": {

"annotation": "",

"id": 18,

"input_connections": {

"input": {

"id": 17,

"output_name": "output1"

}

},

"inputs": [],

"name": "Filter",

"outputs": [

{

"name": "out_file1",

"type": "input"

}

],

"position": {

"left": 3457.2120666503906,

"top": 610.0764312744141

},

"post_job_actions": {

"RenameDatasetActionout_file1": {

"action_arguments": {

"newname": "BLASTevent_hit"

},

"action_type": "RenameDatasetAction",

"output_name": "out_file1"

}

},

"tool_errors": null,

"tool_id": "Filter1",

"tool_state": "{\"input\": \"null\", \"__rerun_remap_job_id__\": null, \"header_lines\": \"\\\"0\\\"\", \"cond\": \"\\\"c4>=(0.97*c24) and c4<=(1.03*c24) and c3>95\\\"\", \"__page__\": 0}",

"tool_version": "1.1.0",

"type": "tool",

"user_outputs": []

},

"19": {

"annotation": "",

"id": 19,

"input_connections": {

"id_opts|input_tabular": {

"id": 18,

"output_name": "out_file1"

},

"input_file": {

"id": 16,

"output_name": "output"

}

},

"inputs": [],

"name": "Filter sequences by ID",

"outputs": [

{

"name": "output_pos",

"type": "fasta"

},

{

"name": "output_neg",

"type": "fasta"

}

],

"position": {

"left": 3728.2189025878906,

"top": 688.0972442626953

},

"post_job_actions": {

"RenameDatasetActionoutput_neg": {

"action_arguments": {

"newname": "BLASTevent_NO_hit"

},

"action_type": "RenameDatasetAction",

"output_name": "output_neg"

},

"RenameDatasetActionoutput_pos": {

"action_arguments": {

"newname": "bin2_BLASTevent_hit"

},

"action_type": "RenameDatasetAction",

"output_name": "output_pos"

}

},

"tool_errors": null,

"tool_id": "testtoolshed.g2.bx.psu.edu/repos/peterjc/seq_filter_by_id/seq_filter_by_id/0.2.2",

"tool_state": "{\"__page__\": 0, \"output_choice_cond\": \"{\\\"output_choice\\\": \\\"both\\\", \\\"__current_case__\\\": 0}\", \"input_file\": \"null\", \"adv_opts\": \"{\\\"strip_suffix\\\": \\\"False\\\", \\\"adv_opts_selector\\\": \\\"advanced\\\", \\\"__current_case__\\\": 1}\", \"__rerun_remap_job_id__\": null, \"id_opts\": \"{\\\"id_opts_selector\\\": \\\"tabular\\\", \\\"columns\\\": {\\\"__class__\\\": \\\"UnvalidatedValue\\\", \\\"value\\\": [\\\"1\\\"]}, \\\"__current_case__\\\": 0, \\\"input_tabular\\\": null}\"}",

"tool_version": "0.2.2",

"type": "tool",

"user_outputs": []

},

"20": {

"annotation": "",

"id": 20,

"input_connections": {

"input": {

"id": 18,

"output_name": "out_file1"

}

},

"inputs": [],

"name": "Cut",

"outputs": [

{

"name": "out_file1",

"type": "tabular"

}

],

"position": {

"left": 3782.3334045410156,

"top": 501.2048797607422

},

"post_job_actions": {},

"tool_errors": null,

"tool_id": "toolshed.g2.bx.psu.edu/repos/devteam/cut_columns/Cut1/1.0.1",

"tool_state": "{\"columnList\": \"\\\"c1,c2\\\"\", \"input\": \"null\", \"delimiter\": \"\\\"T\\\"\", \"__rerun_remap_job_id__\": null, \"__page__\": 0}",

"tool_version": "1.0.1",

"type": "tool",

"user_outputs": []

},

"21": {

"annotation": "",

"id": 21,

"input_connections": {

"query": {

"id": 19,

"output_name": "output_neg"

}

},

"inputs": [],

"name": "NCBI BLAST+ blastn",

"outputs": [

{

"name": "output1",

"type": "tabular"

}

],

"position": {

"left": 4010.8751525878906,

"top": 1055.232681274414

},

"post_job_actions": {},

"tool_errors": null,

"tool_id": "toolshed.g2.bx.psu.edu/repos/devteam/ncbi_blast_plus/ncbi_blastn_wrapper/0.1.00",

"tool_state": "{\"evalue_cutoff\": \"\\\"0.001\\\"\", \"__page__\": 0, \"adv_opts\": \"{\\\"adv_opts_selector\\\": \\\"basic\\\", \\\"__current_case__\\\": 0}\", \"__rerun_remap_job_id__\": null, \"blast_type\": \"\\\"megablast\\\"\", \"db_opts\": \"{\\\"db_opts_selector\\\": \\\"db\\\", \\\"subject\\\": \\\"\\\", \\\"histdb\\\": \\\"\\\", \\\"__current_case__\\\": 0, \\\"database\\\": \\\"GMO_constructs\\\"}\", \"output\": \"{\\\"out_format\\\": \\\"ext\\\", \\\"__current_case__\\\": 1}\", \"query\": \"null\"}",

"tool_version": "0.1.00",

"type": "tool",

"user_outputs": []

},

"22": {

"annotation": "",

"id": 22,

"input_connections": {

"input": {

"id": 20,

"output_name": "out_file1"

}

},

"inputs": [],

"name": "Count",

"outputs": [

{

"name": "out_file1",

"type": "tabular"

}

],

"position": {

"left": 4012.2154846191406,

"top": 502.09376525878906

},

"post_job_actions": {

"RenameDatasetActionout_file1": {

"action_arguments": {

"newname": "identified_GMO"

},

"action_type": "RenameDatasetAction",

"output_name": "out_file1"

}

},

"tool_errors": null,

"tool_id": "Count1",

"tool_state": "{\"column\": \"{\\\"__class__\\\": \\\"UnvalidatedValue\\\", \\\"value\\\": [\\\"1\\\", \\\"2\\\"]}\", \"input\": \"null\", \"__rerun_remap_job_id__\": null, \"delim\": \"\\\"T\\\"\", \"__page__\": 0}",

"tool_version": "1.0.0",

"type": "tool",

"user_outputs": []

},

"23": {

"annotation": "",

"id": 23,

"input_connections": {

"input": {

"id": 21,

"output_name": "output1"

}

},

"inputs": [],

"name": "Filter",

"outputs": [

{

"name": "out_file1",

"type": "input"

}

],

"position": {

"left": 4392.281402587891,

"top": 648.6528167724609

},

"post_job_actions": {

"RenameDatasetActionout_file1": {

"action_arguments": {

"newname": "BLAST_construct_hit"

},

"action_type": "RenameDatasetAction",

"output_name": "out_file1"

}

},

"tool_errors": null,

"tool_id": "Filter1",

"tool_state": "{\"input\": \"null\", \"__rerun_remap_job_id__\": null, \"header_lines\": \"\\\"0\\\"\", \"cond\": \"\\\"c4==c23\\\"\", \"__page__\": 0}",

"tool_version": "1.1.0",

"type": "tool",

"user_outputs": []

},

"24": {

"annotation": "",

"id": 24,

"input_connections": {

"id_opts|input_tabular": {

"id": 21,

"output_name": "output1"

},

"input_file": {

"id": 19,

"output_name": "output_neg"

}

},

"inputs": [],

"name": "Filter sequences by ID",

"outputs": [

{

"name": "output_pos",

"type": "fasta"

},

{

"name": "output_neg",

"type": "fasta"

}

],

"position": {

"left": 4347.333648681641,

"top": 1250.7014923095703

},

"post_job_actions": {

"HideDatasetActionoutput_pos": {

"action_arguments": {},

"action_type": "HideDatasetAction",

"output_name": "output_pos"

}

},

"tool_errors": null,

"tool_id": "testtoolshed.g2.bx.psu.edu/repos/peterjc/seq_filter_by_id/seq_filter_by_id/0.2.2",

"tool_state": "{\"__page__\": 0, \"output_choice_cond\": \"{\\\"output_choice\\\": \\\"neg\\\", \\\"__current_case__\\\": 2}\", \"input_file\": \"null\", \"adv_opts\": \"{\\\"adv_opts_selector\\\": \\\"basic\\\", \\\"__current_case__\\\": 0}\", \"__rerun_remap_job_id__\": null, \"id_opts\": \"{\\\"id_opts_selector\\\": \\\"tabular\\\", \\\"columns\\\": {\\\"__class__\\\": \\\"UnvalidatedValue\\\", \\\"value\\\": [\\\"1\\\"]}, \\\"__current_case__\\\": 0, \\\"input_tabular\\\": null}\"}",

"tool_version": "0.2.2",

"type": "tool",

"user_outputs": []

},

"25": {

"annotation": "",

"id": 25,

"input_connections": {

"id_opts|input_tabular": {

"id": 23,

"output_name": "out_file1"

},

"input_file": {

"id": 19,

"output_name": "output_neg"

}

},

"inputs": [],

"name": "Filter sequences by ID",

"outputs": [

{

"name": "output_pos",

"type": "fasta"

},

{

"name": "output_neg",

"type": "fasta"

}

],

"position": {

"left": 4664.677154541016,

"top": 1046.621597290039

},

"post_job_actions": {

"HideDatasetActionoutput_pos": {

"action_arguments": {},

"action_type": "HideDatasetAction",

"output_name": "output_pos"

}

},

"tool_errors": null,

"tool_id": "testtoolshed.g2.bx.psu.edu/repos/peterjc/seq_filter_by_id/seq_filter_by_id/0.2.2",

"tool_state": "{\"__page__\": 0, \"output_choice_cond\": \"{\\\"output_choice\\\": \\\"both\\\", \\\"__current_case__\\\": 0}\", \"input_file\": \"null\", \"adv_opts\": \"{\\\"adv_opts_selector\\\": \\\"basic\\\", \\\"__current_case__\\\": 0}\", \"__rerun_remap_job_id__\": null, \"id_opts\": \"{\\\"id_opts_selector\\\": \\\"tabular\\\", \\\"columns\\\": {\\\"__class__\\\": \\\"UnvalidatedValue\\\", \\\"value\\\": [\\\"1\\\"]}, \\\"__current_case__\\\": 0, \\\"input_tabular\\\": null}\"}",

"tool_version": "0.2.2",

"type": "tool",

"user_outputs": []

},

"26": {

"annotation": "",

"id": 26,

"input_connections": {

"input": {

"id": 23,

"output_name": "out_file1"

}

},

"inputs": [],

"name": "Cut",

"outputs": [

{

"name": "out_file1",

"type": "tabular"

}

],

"position": {

"left": 4723.895904541016,

"top": 799.8507232666016

},

"post_job_actions": {

"ChangeDatatypeActionout_file1": {

"action_arguments": {

"newtype": "bed"

},

"action_type": "ChangeDatatypeAction",

"output_name": "out_file1"

},

"RenameDatasetActionout_file1": {

"action_arguments": {

"newname": "BLAST_construct_hit_BED"

},

"action_type": "RenameDatasetAction",

"output_name": "out_file1"

}

},

"tool_errors": null,

"tool_id": "toolshed.g2.bx.psu.edu/repos/devteam/cut_columns/Cut1/1.0.1",

"tool_state": "{\"columnList\": \"\\\"c2, c9, c10, c1, c25\\\"\", \"input\": \"null\", \"delimiter\": \"\\\"T\\\"\", \"__rerun_remap_job_id__\": null, \"__page__\": 0}",

"tool_version": "1.0.1",

"type": "tool",

"user_outputs": []

},

"27": {

"annotation": "",

"id": 27,

"input_connections": {

"input": {

"id": 24,

"output_name": "output_neg"

}

},

"inputs": [],

"name": "FASTA-to-Tabular",

"outputs": [

{

"name": "output",

"type": "tabular"

}

],

"position": {

"left": 4735.128814697266,

"top": 1277.8542022705078

},

"post_job_actions": {},

"tool_errors": null,

"tool_id": "toolshed.g2.bx.psu.edu/repos/devteam/fasta_to_tabular/fasta2tab/1.1.0",

"tool_state": "{\"input\": \"null\", \"keep_first\": \"\\\"0\\\"\", \"__rerun_remap_job_id__\": null, \"descr_columns\": \"\\\"1\\\"\", \"__page__\": 0}",

"tool_version": "1.1.0",

"type": "tool",

"user_outputs": []

},

"28": {

"annotation": "",

"id": 28,

"input_connections": {

"input": {

"id": 25,

"output_name": "output_neg"

}

},

"inputs": [],

"name": "FASTA-to-Tabular",

"outputs": [

{

"name": "output",

"type": "tabular"

}

],

"position": {

"left": 5087.368072509766,

"top": 977.1076812744141

},

"post_job_actions": {},

"tool_errors": null,

"tool_id": "toolshed.g2.bx.psu.edu/repos/devteam/fasta_to_tabular/fasta2tab/1.1.0",

"tool_state": "{\"input\": \"null\", \"keep_first\": \"\\\"0\\\"\", \"__rerun_remap_job_id__\": null, \"descr_columns\": \"\\\"1\\\"\", \"__page__\": 0}",

"tool_version": "1.1.0",

"type": "tool",

"user_outputs": []

},

"29": {

"annotation": "",

"id": 29,

"input_connections": {

"input": {

"id": 26,

"output_name": "out_file1"

}

},

"inputs": [],

"name": "Filter",

"outputs": [

{

"name": "out_file1",

"type": "input"

}

],

"position": {

"left": 5131.430572509766,

"top": 646.4132232666016

},

"post_job_actions": {

"RenameDatasetActionout_file1": {

"action_arguments": {

"newname": "OK"

},

"action_type": "RenameDatasetAction",

"output_name": "out_file1"

}

},

"tool_errors": null,

"tool_id": "Filter1",

"tool_state": "{\"input\": \"null\", \"__rerun_remap_job_id__\": null, \"header_lines\": \"\\\"0\\\"\", \"cond\": \"\\\"c2<c3\\\"\", \"__page__\": 0}",

"tool_version": "1.1.0",

"type": "tool",

"user_outputs": []

},

"30": {

"annotation": "",

"id": 30,

"input_connections": {

"input": {

"id": 26,

"output_name": "out_file1"

}

},

"inputs": [],

"name": "Filter",

"outputs": [

{

"name": "out_file1",

"type": "input"

}

],

"position": {

"left": 5135.441314697266,

"top": 814.4340972900391

},

"post_job_actions": {

"RenameDatasetActionout_file1": {

"action_arguments": {

"newname": "turn"

},

"action_type": "RenameDatasetAction",

"output_name": "out_file1"

}

},

"tool_errors": null,

"tool_id": "Filter1",

"tool_state": "{\"input\": \"null\", \"__rerun_remap_job_id__\": null, \"header_lines\": \"\\\"0\\\"\", \"cond\": \"\\\"c2>c3\\\"\", \"__page__\": 0}",

"tool_version": "1.1.0",

"type": "tool",

"user_outputs": []

},

"31": {

"annotation": "",

"id": 31,

"input_connections": {

"input1": {

"id": 27,

"output_name": "output"

},

"queries_0|input2": {

"id": 28,

"output_name": "output"

}

},

"inputs": [],

"name": "Concatenate datasets",

"outputs": [

{

"name": "out_file1",

"type": "input"

}

],

"position": {

"left": 5053.687896728516,

"top": 1270.9097442626953

},

"post_job_actions": {},

"tool_errors": null,

"tool_id": "cat1",

"tool_state": "{\"__page__\": 0, \"__rerun_remap_job_id__\": null, \"input1\": \"null\", \"queries\": \"[{\\\"input2\\\": null, \\\"__index__\\\": 0}]\"}",

"tool_version": "1.0.0",

"type": "tool",

"user_outputs": []

},

"32": {

"annotation": "",

"id": 32,

"input_connections": {

"input": {

"id": 30,

"output_name": "out_file1"

}

},

"inputs": [],

"name": "Cut",

"outputs": [

{

"name": "out_file1",

"type": "tabular"

}

],

"position": {

"left": 5394.677154541016,

"top": 821.6562652587891

},

"post_job_actions": {

"RenameDatasetActionout_file1": {

"action_arguments": {

"newname": "turned"

},

"action_type": "RenameDatasetAction",

"output_name": "out_file1"

}

},

"tool_errors": null,

"tool_id": "toolshed.g2.bx.psu.edu/repos/devteam/cut_columns/Cut1/1.0.1",

"tool_state": "{\"columnList\": \"\\\"c1, c3, c2, c4, c5\\\"\", \"input\": \"null\", \"delimiter\": \"\\\"T\\\"\", \"__rerun_remap_job_id__\": null, \"__page__\": 0}",

"tool_version": "1.0.1",

"type": "tool",

"user_outputs": []

},

"33": {

"annotation": "",

"id": 33,

"input_connections": {

"input": {

"id": 31,

"output_name": "out_file1"

}

},

"inputs": [],

"name": "Tabular-to-FASTA",

"outputs": [

{

"name": "output",

"type": "fasta"

}

],

"position": {

"left": 5331.204986572266,

"top": 1281.9340362548828

},

"post_job_actions": {

"RenameDatasetActionoutput": {

"action_arguments": {

"newname": "input_BLASTelement"

},

"action_type": "RenameDatasetAction",

"output_name": "output"

}

},

"tool_errors": null,

"tool_id": "toolshed.g2.bx.psu.edu/repos/devteam/tabular_to_fasta/tab2fasta/1.1.0",

"tool_state": "{\"title_col\": \"{\\\"__class__\\\": \\\"UnvalidatedValue\\\", \\\"value\\\": [\\\"1\\\"]}\", \"input\": \"null\", \"__page__\": 0, \"__rerun_remap_job_id__\": null, \"seq_col\": \"{\\\"__class__\\\": \\\"UnvalidatedValue\\\", \\\"value\\\": \\\"2\\\"}\"}",

"tool_version": "1.1.0",

"type": "tool",

"user_outputs": []

},

"34": {

"annotation": "",

"id": 34,

"input_connections": {

"input1": {

"id": 29,

"output_name": "out_file1"

},

"queries_0|input2": {

"id": 32,

"output_name": "out_file1"

}

},

"inputs": [],

"name": "Concatenate datasets",

"outputs": [

{

"name": "out_file1",

"type": "input"

}

],

"position": {

"left": 5774.156646728516,

"top": 716.6389312744141

},

"post_job_actions": {

"ChangeDatatypeActionout_file1": {

"action_arguments": {

"newtype": "bed"

},

"action_type": "ChangeDatatypeAction",

"output_name": "out_file1"

},

"RenameDatasetActionout_file1": {

"action_arguments": {

"newname": "BLAST_construct_hit_BED_fixed"

},

"action_type": "RenameDatasetAction",

"output_name": "out_file1"

}

},

"tool_errors": null,

"tool_id": "cat1",

"tool_state": "{\"__page__\": 0, \"__rerun_remap_job_id__\": null, \"input1\": \"null\", \"queries\": \"[{\\\"input2\\\": null, \\\"__index__\\\": 0}]\"}",

"tool_version": "1.0.0",

"type": "tool",

"user_outputs": []

},

"35": {

"annotation": "",

"id": 35,

"input_connections": {

"query": {

"id": 33,

"output_name": "output"

}

},

"inputs": [],

"name": "NCBI BLAST+ blastn",

"outputs": [

{

"name": "output1",

"type": "tabular"

}

],

"position": {

"left": 5538.774810791016,

"top": 1634.9722442626953

},

"post_job_actions": {},

"tool_errors": null,

"tool_id": "toolshed.g2.bx.psu.edu/repos/devteam/ncbi_blast_plus/ncbi_blastn_wrapper/0.1.00",

"tool_state": "{\"evalue_cutoff\": \"\\\"0.001\\\"\", \"__page__\": 0, \"adv_opts\": \"{\\\"adv_opts_selector\\\": \\\"basic\\\", \\\"__current_case__\\\": 0}\", \"__rerun_remap_job_id__\": null, \"blast_type\": \"\\\"megablast\\\"\", \"db_opts\": \"{\\\"db_opts_selector\\\": \\\"file\\\", \\\"subject\\\": null, \\\"histdb\\\": \\\"\\\", \\\"__current_case__\\\": 2, \\\"database\\\": \\\"\\\"}\", \"output\": \"{\\\"out_format\\\": \\\"ext\\\", \\\"__current_case__\\\": 1}\", \"query\": \"null\"}",

"tool_version": "0.1.00",

"type": "tool",

"user_outputs": []

},

"36": {

"annotation": "",

"id": 36,

"input_connections": {

"inputB": {

"id": 34,

"output_name": "out_file1"

}

},

"inputs": [],

"name": "Intersect interval files",

"outputs": [

{

"name": "output",

"type": "bed"

}

],

"position": {

"left": 6075.736236572266,

"top": 697.7326812744141

},

"post_job_actions": {},

"tool_errors": null,

"tool_id": "toolshed.g2.bx.psu.edu/repos/iuc/bedtools/bedtools_intersectbed/2.22.0",

"tool_state": "{\"count\": \"\\\"False\\\"\", \"__page__\": 0, \"reciprocal\": \"\\\"False\\\"\", \"overlap_mode\": \"\\\"-wo\\\"\", \"invert\": \"\\\"False\\\"\", \"inputB\": \"null\", \"header\": \"\\\"False\\\"\", \"inputA\": \"null\", \"split\": \"\\\"False\\\"\", \"fraction\": \"\\\"\\\"\", \"__rerun_remap_job_id__\": null, \"strand\": \"\\\"\\\"\", \"once\": \"\\\"False\\\"\"}",

"tool_version": "2.22.0",

"type": "tool",

"user_outputs": []

},

"37": {

"annotation": "",

"id": 37,

"input_connections": {

"id_opts|input_tabular": {

"id": 35,

"output_name": "output1"

},

"input_file": {

"id": 33,

"output_name": "output"

}

},

"inputs": [],

"name": "Filter sequences by ID",

"outputs": [

{

"name": "output_pos",

"type": "fasta"

},

{

"name": "output_neg",

"type": "fasta"

}

],

"position": {

"left": 5807.750396728516,

"top": 1923.5139923095703

},

"post_job_actions": {

"HideDatasetActionoutput_pos": {

"action_arguments": {},

"action_type": "HideDatasetAction",

"output_name": "output_pos"

}

},

"tool_errors": null,

"tool_id": "testtoolshed.g2.bx.psu.edu/repos/peterjc/seq_filter_by_id/seq_filter_by_id/0.2.2",

"tool_state": "{\"__page__\": 0, \"output_choice_cond\": \"{\\\"output_choice\\\": \\\"both\\\", \\\"__current_case__\\\": 0}\", \"input_file\": \"null\", \"adv_opts\": \"{\\\"adv_opts_selector\\\": \\\"basic\\\", \\\"__current_case__\\\": 0}\", \"__rerun_remap_job_id__\": null, \"id_opts\": \"{\\\"id_opts_selector\\\": \\\"tabular\\\", \\\"columns\\\": {\\\"__class__\\\": \\\"UnvalidatedValue\\\", \\\"value\\\": [\\\"1\\\"]}, \\\"__current_case__\\\": 0, \\\"input_tabular\\\": null}\"}",

"tool_version": "0.2.2",

"type": "tool",

"user_outputs": []

},

"38": {

"annotation": "",

"id": 38,

"input_connections": {

"input": {

"id": 35,

"output_name": "output1"

}

},

"inputs": [],

"name": "Sort",

"outputs": [

{

"name": "out_file1",

"type": "input"

}

],

"position": {

"left": 5933.462310791016,

"top": 1628.9654083251953

},

"post_job_actions": {

"RenameDatasetActionout_file1": {

"action_arguments": {

"newname": "BLAST_element_sorted"

},

"action_type": "RenameDatasetAction",

"output_name": "out_file1"

}

},

"tool_errors": null,

"tool_id": "sort1",

"tool_state": "{\"__page__\": 0, \"style\": \"\\\"alpha\\\"\", \"column\": \"{\\\"__class__\\\": \\\"UnvalidatedValue\\\", \\\"value\\\": \\\"1\\\"}\", \"__rerun_remap_job_id__\": null, \"order\": \"\\\"DESC\\\"\", \"input\": \"null\", \"column_set\": \"[{\\\"other_order\\\": \\\"ASC\\\", \\\"__index__\\\": 0, \\\"other_column\\\": {\\\"__class__\\\": \\\"UnvalidatedValue\\\", \\\"value\\\": \\\"7\\\"}, \\\"other_style\\\": \\\"num\\\"}]\"}",

"tool_version": "1.0.3",

"type": "tool",

"user_outputs": []

},

"39": {

"annotation": "",

"id": 39,

"input_connections": {

"input": {

"id": 36,

"output_name": "output"

}

},

"inputs": [],

"name": "Sort",

"outputs": [

{

"name": "out_file1",

"type": "input"

}

],

"position": {

"left": 6418.028228759766,

"top": 800.0069732666016

},

"post_job_actions": {},

"tool_errors": null,

"tool_id": "sort1",

"tool_state": "{\"__page__\": 0, \"style\": \"\\\"alpha\\\"\", \"column\": \"{\\\"__class__\\\": \\\"UnvalidatedValue\\\", \\\"value\\\": \\\"13\\\"}\", \"__rerun_remap_job_id__\": null, \"order\": \"\\\"DESC\\\"\", \"input\": \"null\", \"column_set\": \"[]\"}",

"tool_version": "1.0.3",

"type": "tool",

"user_outputs": []

},

"40": {

"annotation": "",

"id": 40,

"input_connections": {

"input": {

"id": 37,

"output_name": "output_neg"

}

},

"inputs": [],

"name": "FASTA-to-Tabular",

"outputs": [

{

"name": "output",

"type": "tabular"

}

],

"position": {

"left": 6026.864654541016,

"top": 2201.8125762939453

},

"post_job_actions": {

"HideDatasetActionoutput": {

"action_arguments": {},

"action_type": "HideDatasetAction",

"output_name": "output"

},

"RenameDatasetActionoutput": {

"action_arguments": {

"newname": "potential_UGMO_manual check required_list"

},

"action_type": "RenameDatasetAction",

"output_name": "output"

}

},

"tool_errors": null,

"tool_id": "toolshed.g2.bx.psu.edu/repos/devteam/fasta_to_tabular/fasta2tab/1.1.0",

"tool_state": "{\"input\": \"null\", \"keep_first\": \"\\\"0\\\"\", \"__rerun_remap_job_id__\": null, \"descr_columns\": \"\\\"1\\\"\", \"__page__\": 0}",

"tool_version": "1.1.0",

"type": "tool",

"user_outputs": []

},

"41": {

"annotation": "",

"id": 41,

"input_connections": {

"input": {

"id": 37,

"output_name": "output_neg"

}

},

"inputs": [],

"name": "Derep_prefix",

"outputs": [

{

"name": "output",

"type": "fasta"

}

],

"position": {

"left": 6151.639068603516,

"top": 2062.020950317383

},

"post_job_actions": {

"RenameDatasetActionoutput": {

"action_arguments": {

"newname": "potential_UGMO_manual check required"

},

"action_type": "RenameDatasetAction",

"output_name": "output"

}

},

"tool_errors": null,

"tool_id": "Derep_prefix",

"tool_state": "{\"input\": \"null\", \"__rerun_remap_job_id__\": null, \"__page__\": 0}",

"tool_version": "1.0.0M",

"type": "tool",

"user_outputs": []

},

"42": {

"annotation": "",

"id": 42,

"input_connections": {

"input": {

"id": 38,

"output_name": "out_file1"

}

},

"inputs": [],

"name": "Cut",

"outputs": [

{

"name": "out_file1",

"type": "tabular"

}

],

"position": {

"left": 6193.427154541016,

"top": 1626.9341583251953

},

"post_job_actions": {

"ChangeDatatypeActionout_file1": {

"action_arguments": {

"newtype": "bed"

},

"action_type": "ChangeDatatypeAction",

"output_name": "out_file1"

},

"RenameDatasetActionout_file1": {

"action_arguments": {

"newname": "BLAST_element_output"

},

"action_type": "RenameDatasetAction",

"output_name": "out_file1"

}

},

"tool_errors": null,

"tool_id": "toolshed.g2.bx.psu.edu/repos/devteam/cut_columns/Cut1/1.0.1",

"tool_state": "{\"columnList\": \"\\\"c1, c23, c2, c24, c7, c8, c9, c10, c4\\\"\", \"input\": \"null\", \"delimiter\": \"\\\"T\\\"\", \"__rerun_remap_job_id__\": null, \"__page__\": 0}",

"tool_version": "1.0.1",

"type": "tool",

"user_outputs": []

},

"43": {

"annotation": "",

"id": 43,

"input_connections": {

"input": {

"id": 39,

"output_name": "out_file1"

}

},

"inputs": [],

"name": "Cut",

"outputs": [

{

"name": "out_file1",

"type": "tabular"

}

],

"position": {

"left": 6745.041900634766,

"top": 720.4583892822266

},

"post_job_actions": {},

"tool_errors": null,

"tool_id": "toolshed.g2.bx.psu.edu/repos/devteam/cut_columns/Cut1/1.0.1",

"tool_state": "{\"columnList\": \"\\\"c13, c11, c12, c14, c1, c4, c5, c9, c15\\\"\", \"input\": \"null\", \"delimiter\": \"\\\"T\\\"\", \"__rerun_remap_job_id__\": null, \"__page__\": 0}",

"tool_version": "1.0.1",

"type": "tool",

"user_outputs": []

},

"44": {

"annotation": "",

"id": 44,

"input_connections": {

"input": {

"id": 40,

"output_name": "output"

}

},

"inputs": [],

"name": "Cut",

"outputs": [

{

"name": "out_file1",

"type": "tabular"

}

],

"position": {

"left": 6261.031646728516,

"top": 2204.989700317383

},

"post_job_actions": {

"RenameDatasetActionout_file1": {

"action_arguments": {

"newname": "potential_UGMO_manual check required_list"

},

"action_type": "RenameDatasetAction",

"output_name": "out_file1"

}

},

"tool_errors": null,

"tool_id": "toolshed.g2.bx.psu.edu/repos/devteam/cut_columns/Cut1/1.0.1",

"tool_state": "{\"columnList\": \"\\\"c1\\\"\", \"input\": \"null\", \"delimiter\": \"\\\"T\\\"\", \"__rerun_remap_job_id__\": null, \"__page__\": 0}",

"tool_version": "1.0.1",

"type": "tool",

"user_outputs": []

},

"45": {

"annotation": "groupe certain columns",

"id": 45,

"input_connections": {

"infile": {

"id": 42,

"output_name": "out_file1"

}

},

"inputs": [],

"name": "Text reformatting",

"outputs": [

{

"name": "outfile",

"type": "txt"

}

],

"position": {

"left": 6449.434478759766,

"top": 1646.7952423095703

},

"post_job_actions": {

"HideDatasetActionoutfile": {

"action_arguments": {},

"action_type": "HideDatasetAction",

"output_name": "outfile"

}

},

"tool_errors": null,

"tool_id": "toolshed.g2.bx.psu.edu/repos/bgruening/text_processing/tp_awk_tool/1.0.0",

"tool_state": "{\"__page__\": 0, \"__rerun_remap_job_id__\": null, \"code\": \"\\\"{b=$1\\\\\\\"_\\\\\\\"$2}\\\\n{c=$3\\\\\\\" \\\\\\\"$4\\\\\\\" \\\\\\\"$5\\\\\\\" \\\\\\\"$6\\\\\\\" \\\\\\\"$7\\\\\\\" \\\\\\\"$8}\\\\n\\\\n{print b, c}\\\"\", \"infile\": \"null\"}",

"tool_version": "1.0.0",

"type": "tool",

"user_outputs": []

},

"46": {

"annotation": "groupe certain columns",

"id": 46,

"input_connections": {

"infile": {

"id": 43,

"output_name": "out_file1"

}

},

"inputs": [],

"name": "Text reformatting",

"outputs": [

{

"name": "outfile",

"type": "txt"

}

],

"position": {

"left": 6759.538482666016,

"top": 854.4687652587891

},

"post_job_actions": {

"HideDatasetActionoutfile": {

"action_arguments": {},

"action_type": "HideDatasetAction",

"output_name": "outfile"

}

},

"tool_errors": null,

"tool_id": "toolshed.g2.bx.psu.edu/repos/bgruening/text_processing/tp_awk_tool/1.0.0",

"tool_state": "{\"__page__\": 0, \"__rerun_remap_job_id__\": null, \"code\": \"\\\"{b=$1\\\\\\\" \\\\\\\"$2\\\\\\\" \\\\\\\"$3\\\\\\\" \\\\\\\"$4\\\\\\\" \\\\\\\"$5}\\\\n{c=$6\\\\\\\" \\\\\\\"$7\\\\\\\" \\\\\\\"$8}\\\\n{d=$9}\\\\n{print b, c, d}\\\"\", \"infile\": \"null\"}",

"tool_version": "1.0.0",

"type": "tool",

"user_outputs": []

},

"47": {

"annotation": "change the delimiter to tab, make every colum a record",

"id": 47,

"input_connections": {

"infile": {

"id": 45,

"output_name": "outfile"

}

},

"inputs": [],

"name": "Text reformatting",

"outputs": [

{

"name": "outfile",

"type": "txt"

}

],

"position": {

"left": 6487.403228759766,

"top": 1764.7813262939453

},

"post_job_actions": {

"HideDatasetActionoutfile": {

"action_arguments": {},

"action_type": "HideDatasetAction",

"output_name": "outfile"

}

},

"tool_errors": null,

"tool_id": "toolshed.g2.bx.psu.edu/repos/bgruening/text_processing/tp_awk_tool/1.0.0",

"tool_state": "{\"__page__\": 0, \"__rerun_remap_job_id__\": null, \"code\": \"\\\"BEGIN{\\\\nRS=\\\\\\\"\\\\\\\\t\\\\\\\"\\\\n}\\\\n{\\\\nprint $0\\\\n}\\\"\", \"infile\": \"null\"}",

"tool_version": "1.0.0",

"type": "tool",

"user_outputs": []

},

"48": {

"annotation": "change the delimiter to tab, make every colum a record",

"id": 48,

"input_connections": {

"infile": {

"id": 46,

"output_name": "outfile"

}

},

"inputs": [],

"name": "Text reformatting",

"outputs": [

{

"name": "outfile",

"type": "txt"

}

],

"position": {

"left": 6761.587310791016,

"top": 956.5347442626953

},

"post_job_actions": {

"HideDatasetActionoutfile": {

"action_arguments": {},

"action_type": "HideDatasetAction",

"output_name": "outfile"

}

},

"tool_errors": null,

"tool_id": "toolshed.g2.bx.psu.edu/repos/bgruening/text_processing/tp_awk_tool/1.0.0",

"tool_state": "{\"__page__\": 0, \"__rerun_remap_job_id__\": null, \"code\": \"\\\"BEGIN{\\\\nRS=\\\\\\\"\\\\\\\\t\\\\\\\"\\\\n}\\\\n{\\\\nprint $0\\\\n}\\\"\", \"infile\": \"null\"}",

"tool_version": "1.0.0",

"type": "tool",

"user_outputs": []

},

"49": {

"annotation": "duplicates",

"id": 49,

"input_connections": {

"infile": {

"id": 47,

"output_name": "outfile"

}

},

"inputs": [],

"name": "Text reformatting",

"outputs": [

{

"name": "outfile",

"type": "txt"

}

],

"position": {

"left": 6559.451568603516,

"top": 1872.8195343017578

},

"post_job_actions": {

"HideDatasetActionoutfile": {

"action_arguments": {},

"action_type": "HideDatasetAction",

"output_name": "outfile"

}

},

"tool_errors": null,

"tool_id": "toolshed.g2.bx.psu.edu/repos/bgruening/text_processing/tp_awk_tool/1.0.0",

"tool_state": "{\"__page__\": 0, \"__rerun_remap_job_id__\": null, \"code\": \"\\\"{ if (a[$1]++ == 0) print $0 }\\\"\", \"infile\": \"null\"}",

"tool_version": "1.0.0",

"type": "tool",

"user_outputs": []

},

"50": {

"annotation": "duplicates",

"id": 50,

"input_connections": {

"infile": {

"id": 48,

"output_name": "outfile"

}

},

"inputs": [],

"name": "Text reformatting",

"outputs": [

{

"name": "outfile",

"type": "txt"

}

],

"position": {

"left": 6764.625396728516,

"top": 1063.5659942626953

},

"post_job_actions": {

"HideDatasetActionoutfile": {

"action_arguments": {},

"action_type": "HideDatasetAction",

"output_name": "outfile"

}

},

"tool_errors": null,

"tool_id": "toolshed.g2.bx.psu.edu/repos/bgruening/text_processing/tp_awk_tool/1.0.0",

"tool_state": "{\"__page__\": 0, \"__rerun_remap_job_id__\": null, \"code\": \"\\\"{ if (a[$1]++ == 0) print $0; }\\\"\", \"infile\": \"null\"}",

"tool_version": "1.0.0",

"type": "tool",

"user_outputs": []

},

"51": {

"annotation": "insert a line before every new ccs",

"id": 51,

"input_connections": {

"infile": {

"id": 49,

"output_name": "outfile"

}

},

"inputs": [],

"name": "Text transformation",

"outputs": [

{

"name": "output",

"type": "txt"

}

],

"position": {

"left": 6671.639068603516,

"top": 1988.9827423095703

},

"post_job_actions": {

"HideDatasetActionoutput": {

"action_arguments": {},

"action_type": "HideDatasetAction",

"output_name": "output"

}

},

"tool_errors": null,

"tool_id": "toolshed.g2.bx.psu.edu/repos/bgruening/text_processing/tp_sed_tool/1.0.0",

"tool_state": "{\"adv_opts\": \"{\\\"adv_opts_selector\\\": \\\"basic\\\", \\\"__current_case__\\\": 0}\", \"__page__\": 0, \"__rerun_remap_job_id__\": null, \"code\": \"\\\"/m[0-9]/i \\\\\\\\\\\\\\\\\\\"\", \"infile\": \"null\"}",

"tool_version": "1.0.0",

"type": "tool",

"user_outputs": []

},

"52": {

"annotation": "insert a line before every new ccs",

"id": 52,

"input_connections": {

"infile": {

"id": 50,

"output_name": "outfile"

}

},

"inputs": [],

"name": "Text transformation",

"outputs": [

{

"name": "output",

"type": "txt"

}

],

"position": {

"left": 6767.975982666016,

"top": 1174.3646392822266

},

"post_job_actions": {

"HideDatasetActionoutput": {

"action_arguments": {},

"action_type": "HideDatasetAction",

"output_name": "output"

}

},

"tool_errors": null,

"tool_id": "toolshed.g2.bx.psu.edu/repos/bgruening/text_processing/tp_sed_tool/1.0.0",

"tool_state": "{\"adv_opts\": \"{\\\"adv_opts_selector\\\": \\\"basic\\\", \\\"__current_case__\\\": 0}\", \"__page__\": 0, \"__rerun_remap_job_id__\": null, \"code\": \"\\\"/m[0-9]/i \\\\\\\\\\\\\\\\\\\"\", \"infile\": \"null\"}",

"tool_version": "1.0.0",

"type": "tool",

"user_outputs": []

},

"53": {

"annotation": "multiline to tab",

"id": 53,

"input_connections": {

"infile": {

"id": 51,

"output_name": "output"

}

},

"inputs": [],

"name": "Text reformatting",

"outputs": [

{

"name": "outfile",

"type": "txt"

}

],

"position": {

"left": 6827.576568603516,

"top": 2103.9480743408203

},

"post_job_actions": {

"HideDatasetActionoutfile": {

"action_arguments": {},

"action_type": "HideDatasetAction",

"output_name": "outfile"

}

},

"tool_errors": null,

"tool_id": "toolshed.g2.bx.psu.edu/repos/bgruening/text_processing/tp_awk_tool/1.0.0",

"tool_state": "{\"__page__\": 0, \"__rerun_remap_job_id__\": null, \"code\": \"\\\"BEGIN { \\\\n FS=\\\\\\\"\\\\\\\\n\\\\\\\" \\\\n RS=\\\\\\\"\\\\\\\" \\\\n ORS=\\\\\\\"\\\\\\\" \\\\n} \\\\n \\\\n{ \\\\n x=1 \\\\n while ( x<NF ) { \\\\n print $x \\\\\\\" \\\\\\\" \\\\n x++ \\\\n } \\\\n print $NF \\\\\\\"\\\\\\\\n\\\\\\\" \\\\n}\\\"\", \"infile\": \"null\"}",

"tool_version": "1.0.0",

"type": "tool",

"user_outputs": []

},

"54": {

"annotation": "multiline to tab",

"id": 54,

"input_connections": {

"infile": {

"id": 52,

"output_name": "output"

}

},

"inputs": [],

"name": "Text reformatting",

"outputs": [

{

"name": "outfile",

"type": "txt"

}

],

"position": {

"left": 6771.621978759766,

"top": 1278.5659942626953

},

"post_job_actions": {

"HideDatasetActionoutfile": {

"action_arguments": {},

"action_type": "HideDatasetAction",

"output_name": "outfile"

}

},

"tool_errors": null,

"tool_id": "toolshed.g2.bx.psu.edu/repos/bgruening/text_processing/tp_awk_tool/1.0.0",

"tool_state": "{\"__page__\": 0, \"__rerun_remap_job_id__\": null, \"code\": \"\\\"BEGIN { \\\\n FS=\\\\\\\"\\\\\\\\n\\\\\\\" \\\\n RS=\\\\\\\"\\\\\\\" \\\\n ORS=\\\\\\\"\\\\\\\" \\\\n} \\\\n \\\\n{ \\\\n x=1 \\\\n while ( x<NF ) { \\\\n print $x \\\\\\\" \\\\\\\" \\\\n x++ \\\\n } \\\\n print $NF \\\\\\\"\\\\\\\\n\\\\\\\" \\\\n}\\\"\", \"infile\": \"null\"}",

"tool_version": "1.0.0",

"type": "tool",

"user_outputs": []

},

"55": {

"annotation": "whitespace to tab",

"id": 55,

"input_connections": {

"input": {

"id": 53,

"output_name": "outfile"

}

},

"inputs": [],

"name": "Convert",

"outputs": [

{

"name": "out_file1",

"type": "tabular"

}

],

"position": {

"left": 7041.656646728516,

"top": 2104.0174102783203

},

"post_job_actions": {

"RenameDatasetActionout_file1": {

"action_arguments": {

"newname": "element_order_list_2"

},

"action_type": "RenameDatasetAction",

"output_name": "out_file1"

}

},

"tool_errors": null,

"tool_id": "Convert characters1",

"tool_state": "{\"input\": \"null\", \"__rerun_remap_job_id__\": null, \"convert_from\": \"\\\"s\\\"\", \"__page__\": 0}",

"tool_version": "1.0.0",

"type": "tool",

"user_outputs": []

},

"56": {

"annotation": "",

"id": 56,

"input_connections": {

"input": {

"id": 54,

"output_name": "outfile"

}

},

"inputs": [],

"name": "Convert",

"outputs": [

{

"name": "out_file1",

"type": "tabular"

}

],

"position": {

"left": 6769.573150634766,

"top": 1392.9931182861328

},

"post_job_actions": {

"RenameDatasetActionout_file1": {

"action_arguments": {

"newname": "element_order_list_1"

},

"action_type": "RenameDatasetAction",

"output_name": "out_file1"

}

},

"tool_errors": null,

"tool_id": "Convert characters1",

"tool_state": "{\"input\": \"null\", \"__rerun_remap_job_id__\": null, \"convert_from\": \"\\\"s\\\"\", \"__page__\": 0}",

"tool_version": "1.0.0",

"type": "tool",

"user_outputs": []

}

}

}
